# Drivers of stunting reduction in Peru: a country case study

**DOI:** 10.1093/ajcn/nqaa164

**Published:** 2020-08-29

**Authors:** Luis Huicho, Elisa Vidal-Cárdenas, Nadia Akseer, Samanpreet Brar, Kaitlin Conway, Muhammad Islam, Elisa Juarez, Aviva I Rappaport, Hana Tasic, Tyler Vaivada, Jannah Wigle, Zulfiqar A Bhutta

**Affiliations:** Research Center for Integral and Sustainable Development, Cayetano Heredia University, Lima, Peru; Maternal and Child Health Research Center, Cayetano Heredia University, Lima, Peru; School of Medicine, Cayetano Heredia University, Lima, Peru; Research Center for Integral and Sustainable Development, Cayetano Heredia University, Lima, Peru; Maternal and Child Health Research Center, Cayetano Heredia University, Lima, Peru; Centre for Global Child Health, Hospital for Sick Children, Toronto, Ontario, Canada; Dalla Lana School of Public Health, University of Toronto, Toronto, Ontario, Canada; Centre for Global Child Health, Hospital for Sick Children, Toronto, Ontario, Canada; Centre for Global Child Health, Hospital for Sick Children, Toronto, Ontario, Canada; Centre for Global Child Health, Hospital for Sick Children, Toronto, Ontario, Canada; Center for the Promotion and Defense of Sexual and Reproductive Rights (PROMSEX), Lima, Peru; Centre for Global Child Health, Hospital for Sick Children, Toronto, Ontario, Canada; Centre for Global Child Health, Hospital for Sick Children, Toronto, Ontario, Canada; Centre for Global Child Health, Hospital for Sick Children, Toronto, Ontario, Canada; Centre for Global Child Health, Hospital for Sick Children, Toronto, Ontario, Canada; Dalla Lana School of Public Health, University of Toronto, Toronto, Ontario, Canada; Centre for Global Child Health, Hospital for Sick Children, Toronto, Ontario, Canada; Dalla Lana School of Public Health, University of Toronto, Toronto, Ontario, Canada; Center of Excellence in Women and Child Health, The Aga Khan University, Karachi, Pakistan

**Keywords:** stunting, linear growth, children, nutrition, exemplar, Peru, Latin America, mixed methods

## Abstract

**Background:**

Peru reduced its under-5 child stunting prevalence notably from 31.3% in 2000 to 13.1% in 2016.

**Objectives:**

We aimed to study factors and key enablers of child stunting reduction in Peru from 2000–2016.

**Methods:**

Demographic and Health Surveys were used to conduct descriptive analyses [height-for-age *z* scores (HAZ) means and distributions, equity analysis, predicted child growth curves through polynomial regressions] and advanced regression analyses. An ecological (at department level) multilevel regression analysis was conducted to identify the major predictors of stunting decline from 2000 to 2016, and Oaxaca–Blinder decomposition was conducted to identify the relative contribution of each factor to child HAZ change. A systematic literature review, policy and program analysis, and interviews with relevant stakeholders were conducted to understand key drivers of stunting decline in Peru.

**Results:**

The distribution of HAZ scores showed a slight rightward shift from 2000 to 2007/2008, and a greater shift from 2007/2008 to 2016. Stunting reduction was higher in the lowest wealth quintile, in rural areas, and among children with the least educated mothers. Decomposing predicted changes showed that the most important factors were increased maternal BMI and maternal height, improved maternal and newborn health care, increased parental education, migration to urban areas, and reduced fertility. Key drivers included the advocacy role of civil society and political leadership around poverty and stunting reduction since the early 2000s. Key enablers included the economic growth and the consolidation of democracy since the early 2000s, and the acknowledgement that stunting reduction needs much more than food supplementation.

**Conclusions:**

Peru reduced child stunting owing to improved socioeconomic determinants, sustained implementation of out-of-health-sector and within-health-sector changes, and implementation of health interventions. These efforts were driven through a multisectoral approach, strong civil society advocacy, and keen political leadership. Peru's experience offers useful lessons on how to tackle the problem of stunting under differing scenarios, with the participation of multiple sectors.

## Introduction

**FIGURE 1:**
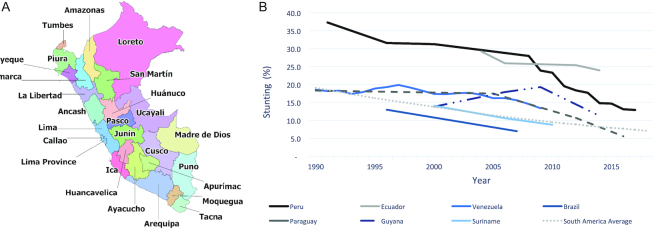
Map of Peru and regional under-5 stunting prevalence. (A) Map of Peru. (B) The prevalence of under-5 stunting in selected South American countries, 1990–2018. Source: Joint Malnutrition Estimates (4).

Many countries made commitments toward achieving the Millennium Development Goals, and prominent among them were those aimed at reducing maternal and child mortality and malnutrition (1). Low- and middle-income countries in diverse regions of the world have achieved significant reductions in their under-5 stunting prevalence, and Peru (**Figure 1**A) (2) has been one of these exemplary countries. Child stunting is defined as a height-for-age *z* score (HAZ) that is more than 2 SDs below the global median and is used as a marker of chronic childhood undernutrition. In the early 1990s, Peru had a high prevalence of stunting, >35%, which remained generally stagnant for over a decade. However, between 2008 and 2016, the stunting prevalence fell to 15% (Figure 1B). Peru not only outperformed its South American counterparts (Figure 1B) but also the global average decline from 32.5% in 2000 to 21.9% in 2018 (3, 4).

Peru is an upper middle-income country that had a population of 31.8 million in 2016, with 21% living in rural areas (5). Indigenous groups comprise more than one-third of the population (5). Since the 2000s, Peru has gained political stability and democracy, after the end of a period of social unrest from the 1980s to the early 1990s (6, 7). High rates of migration from rural to urban areas during the instability and conflict period resulted in a reduction of the rural population to only 28% by 2010 (8). Peru has made substantial economic gains in recent years, reduced inequalities, and improved adult and adolescent fertility rates. Concurrently, it increased women's literacy and access to improved water and sanitation facilities, yet urban–rural disparities have persisted. Trends in these various contextual characteristics of Peru between 2000 and 2016 are presented in **Supplemental Appendix 1**.

Various studies have explored the determinants of stunting reduction in Peru, but most of them involved only national-level analyses. Moreover, they used either qualitative or quantitative approaches, but not both combined (8–14). Only 1 of them has been done at a subnational level (14). Some of the strongest drivers of stunting reduction identified through the literature review and confirmed through quantitative subnational analyses include poverty alleviation and economic growth (8, 10, 15–17), health spending (10, 18–22), population literacy (8, 23), decreasing fertility rates (6, 24–26), utilization of maternal care services (27–29), and access to improved water (30) and hygienic conditions (8), among other factors, hinting at a multifactorial stunting success story (14). Results of the full systematic literature review are presented in **Panel 1** and **Supplemental Appendix 2** (8–12, 14–20, 31–97).

Panel 1 **Systematic literature review of stunting determinants**

Our literature review found that among basic causes of child stunting reduction in Peru, various factors were found to be strongly related including the rapid economic growth (8–10, 18, 93, 97), social welfare programs (8, 10, 15–17, 19, 31), rurality of residence (32–42), altitude (39–42), strong political commitment (8, 10–12, 43), Integrated Management of Childhood Illness (IMCI), and vaccine coverage (22). Peru's economy grew rapidly between 2002 and 2010 (8, 18), and economic growth was estimated to account for upwards of half of national stunting reduction (9). Stunting decline and economic growth may not be directly related, because there exists a gap in time between growth in the economy and stunting reduction (8), although there is an indirect relation through social welfare programs (15). Although many studies found that the poverty-reduction programs Juntos and CRECER had a positive association with stunting reduction (8, 10, 16, 17, 19, 31), some found no association (14, 43, 45). Data suggest that the health insurance program SIS improved poor Peruvians’ access to health care (10, 18), whereas Juntos increased their utilization of services (20). Stunting decline was observed in both rural and urban areas of the country (37, 38). Public spending directed at rural areas increased poor families’ access to health care for their children (19, 21). IMCI implementation at community level and vaccine coverage were correlated with HAZ decline from 1996 to 2000 although a causal correlation could not be ascertained (22), and exploration of health care access showed there was room for improvement (44, 47, 48).

The literature indicates that underlying causes of stunting decline in Peru were improvements in water, hygiene, and sanitation (8, 30, 46, 49), maternal education (23, 36, 42, 50–59, 61, 97), food availability combined with family income (49, 62), and food security (60, 63–65). Untreated drinking water and poor hygiene and sanitation have been found to be associated with HAZ and stunting in 3 studies (30, 46, 49), although 1 study on increased access to safe water found a reduction in diarrhea, but no HAZ difference (66). Both formal schooling (22, 36, 46, 50–58, 97) and alternative education programs teaching caregiving practices positively affected HAZ (23, 59). More calories per capita were available in Peru during 2005–2011 than during 1996–2005 (8). Although food availability has been linked to changes in HAZ (62), family income is also important, as it relates to dietary intake (49). Microloans and Juntos have been shown to improve food security (20, 67), and higher household consumption has been linked to height gain in children (60, 63–65).

Immediate causes of stunting in Peru include disease, infection (68–74), poor dietary intake (36), lack of breastfeeding (76, 77), poor infant and young child feeding (77), low birth weight (78–81, 93), maternal age, and maternal height (52, 80, 82). Infection and diarrhea have been found to be associated with HAZ in numerous studies (68–74), whereas 1 found no association (83). Data show that poor dietary intake is associated with stunting in Peru (36), although neither prenatal supplementation of zinc, nor infant zinc supplementation (84–87), nor multiple micronutrient supplementation have been shown to affect HAZ (88–91). A positive association was found, however, between stunting and age (52, 57, 75, 81, 86, 92, 94–97).

In this study, we aimed to identify the diverse drivers behind Peru's reduction in the prevalence of child stunting using a mixed-methods approach.

We conducted a systematic in-depth assessment of the determinants of stunting decline in Peru, specifically *1*) national- (macro), *2*) community-, household-, *3*) individual-level factors, and *4*) relevant nutrition-specific and nutrition-sensitive interventions/innovations/policies/strategies. We limited our time frame from 2000 to 2016, with a focus on important transitional periods (2000–2007, 2008–2016). We made the decision to split our study into these 2 periods because the pace of stunting decline during the first period was very slow, whereas it was substantially quicker during the second period. Furthermore, we aimed to quantitatively examine determinants of stunting reduction in Peru through a secondary analysis of existing Demographic and Health Survey (DHS) data and to decompose long-term stunting change into relative contribution from key drivers. We also aimed to generate a systematic landscape of the major stunting-relevant policies and programs in Peru, with a focus on both nutrition-specific and nutrition-sensitive initiatives. Finally, we aimed to understand national stakeholder perspectives on Peru's nutrition evolution (focused on progress in stunting reduction) and the major contributing factors behind it.

## Methods

### Study design

We conducted a mixed-methods study that applied several complementary approaches to inform the study objectives including a systematic literature review, a retrospective quantitative analysis, a policy and program analysis from 2000 to 2016, and qualitative data collection and analyses. An adapted conceptual framework was designed based on the UNICEF Nutrition Framework and the Lancet Nutrition framework to guide all analyses (98, 99). For further details on the framework we refer the reader to the stunting methods article in this supplement.

Ethical review was not deemed necessary for the qualitative component, because participants were asked about the discussion topics in the course of, or as an extension of, their work. Ethics approval for the broader stunting case study was obtained through the Research Ethics Board at the Hospital for Sick Children (SickKids), in Toronto, Canada.

### Systematic literature review

We conducted a systematic search of peer-reviewed and gray literature, to synthesize information on contextual factors, interventions, policies, strategies, programs, and initiatives that may have contributed to reductions in child stunting in Peru over time. We searched 15 online databases and several gray literature sources including government and UN websites (Supplemental Appendix 2). We searched for articles published between 1990 and 2017 with no language restriction. Three broad categories of search terms were used: the disease outcome (“stunting”), the population (“child”), and the country of interest (“Peru”). Keywords representing these terms were combined with Boolean operators, adapted with appropriate syntax, and executed in multiple databases. An example of a search syntax is as follows: *1*) stunting: “stunting” or “linear growth” or “linear growth stunting” or “HAZ” or “height” or “height-for-age” or “LAZ” or “length” or “length-for-age”; *2*) child: “child” or “infant”; *3*) Peru: “Peru”; *4*) 1 AND 2 AND 3. Initial database searches returned 1120 records, which were reduced to 547 after deduplication. Applying the screening criteria to titles and abstracts left 134 records (Supplemental Appendix 2). For more detailed methodology on the literature review, see Supplemental Appendix 2.

### Quantitative methods

#### Data sources

Peru's series of DHSs (2000–2016) were the primary quantitative data sets used in this study. The DHSs are nationally representative household surveys that provide data for a wide range of monitoring and impact evaluation indicators in the areas of population, health, and nutrition (100). Details on DHS methodology and content areas are available elsewhere (100). Peru had a DHS round in 2000, 2004–2006, 2007–2008, and annually from 2009 onward. In this study, we used the 2000, 2007/2008, and 2016 survey rounds for analysis; **Table 1** provides the available under-5 y sample sizes for anthropometry data.

**TABLE 1:** Sample size by survey year based on valid child anthropometric data^1^

	Year of DHS survey		
Age group	2000	2007/2008	2016
<6 mo	994	889	1372
6–23 mo	3251	3037	5915
≥24 mo	4603	4363	9712
<36 mo	6018	5600	10,949
<5 y	8848	8289	16,999

1 Based on index child data.

#### Outcomes and predictors

**FIGURE 2:**
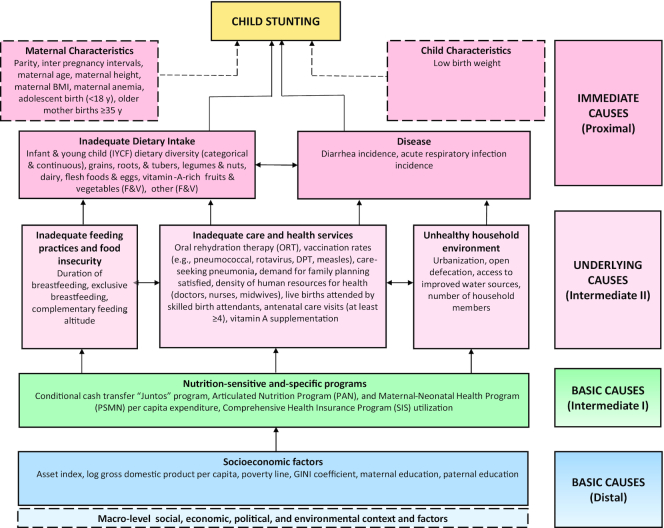
Conceptual framework showing distal, intermediate, and proximal determinants of stunting. Framework represents all variables that were identified and available for quantitative analysis.

The main study outcomes were child HAZ and stunting prevalence (HAZ < −2SD), and these were estimated using WHO child growth standards (101). Covariables were selected in line with **Figure 2** as available in DHS surveys (individual/household variables) and other national household surveys (Encuesta Nacional de Hogares), census data, and data from specific programs (ecological variables at district level). Potential determinants were grouped into hierarchical levels as distal, intermediate, and proximal factors to align with “basic causes,” “underlying causes,” and “immediate causes” in Figure 2.

#### Statistical analysis

For the quantitative component, we determined the distribution of HAZ in children under-5 from 2000 to 2016 (Kernel density plots). We predicted child growth curves (predicted HAZ), which were estimated from smoothed local polynomial regressions and plotted against child age (102, 103). We then determined the under-5 stunting prevalence by departments and by different equity dimensions (wealth quintile, maternal educational level, area of residence, child sex) over time. We also obtained the variation of the slope index of inequality (SII) and of the concentration index (CIX) from 2000 to 2016, to measure absolute and relative socioeconomic inequalities, respectively (104). We conducted a linear mixed-effect regression analysis using department-level changes in stunting from 2000 to 2016 to understand the major predictors of stunting decline in Peru at the subnational level. Finally, we performed a Oaxaca–Blinder decomposition analysis to identify the relative contribution of each factor to HAZ change in Peru from 2000 to 2016. Full methods details are included in **Supplemental Appendix 3** and the methods article in this series (Akseer et al.). All analyses were conducted with Stata version 14.0 (StataCorp LLC) and accounted for survey design and weighting.

### Policy and program review

We conducted a policy and program analysis for the period 1975–2015 through an iterative discussion approach, based on a timeline of key nutrition-specific and nutrition-sensitive policies and programs. A suggested timeline was proposed by the Peru study principal investigator and research team members through a desk-review, which was shared with members during group discussions and in-depth interviews to obtain their corroboration and insight.

### Qualitative methods

The qualitative component of the study involved a group discussion and in-depth interviews conducted in Lima, Peru. The aim was to understand national stakeholder perspectives on Peru's nutrition evolution focused on progress in stunting reduction, and the major contributing factors behind it, and to access key sources of data related to nutrition-specific and -sensitive initiatives implemented in Peru. Through purposive sampling methods, we selected a group of 6 individuals from governmental and nongovernmental organizations, academia, and bilateral and multilateral organizations. These individuals were involved in the design and implementation of policies, programs, and interventions that may have influenced under-5 stunting reduction from 2000 to 2016 in Peru, and were in a unique position to discuss the implementation of relevant policies and programs. During the group discussion session, these experts identified key factors underlying the stunting reduction. We also conducted in-depth interviews with 10 individuals from different organizations, with the same objective of identifying drivers and enablers behind Peru's progress in stunting reduction. Interviewees were selected based on their familiarity with policies and programs implemented in Peru that may have influenced stunting reduction. The interviews were conducted after the focus group discussion, allowing us to explore aspects that were highlighted during the group discussion in further detail. **Supplemental Appendix 4** details the full qualitative data collection and analysis methods.

## Results

### Descriptive analyses

#### HAZ kernel density plots and Victora curves

**FIGURE 3:**
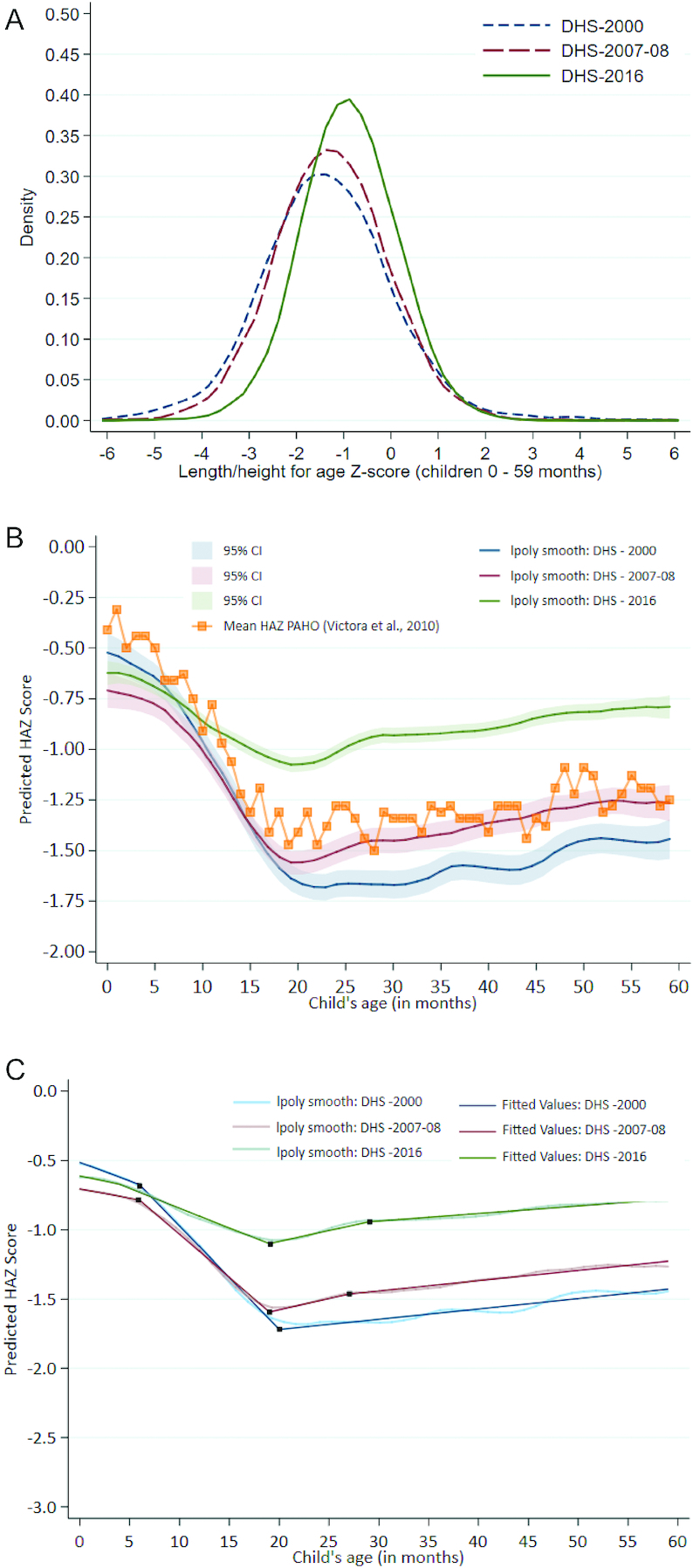
HAZ distribution and growth faltering among children <5 in Peru (A) Kernel density plot for HAZ distribution in children <5 y from 2000 to 2016. (B) Victora curve using data from the 2000, 2007/2008, and 2016 surveys among children <5 y, including PAHO mean HAZ curve (103). (C) Victora curve using data from the 2000, 2007/2008, and 2016 surveys among children <5 y with linear splines. DHS, Demographic and Health Survey; HAZ, height-for-age *z* score; PAHO, Latin America and the Caribbean.

The distribution of HAZ scores had a slight parallel rightward shift from 2000 to 2007/2008, and a much greater shift accompanied by narrowing of the distribution (higher kurtosis) from 2007/2008 to 2016 (**Figure 3**A). Corroborating national stunting trend estimates, HAZ improvements were much more pronounced in the latter time period. The higher peaked distribution in 2016 suggests nutritional gains across the entire population with many children clustering around a common mean HAZ. Overall, the mean HAZ improved from −1.24 (2000) to −1.21 (2007/2008) to −0.84 (2016).

Regarding children's HAZ trajectory between the low stunting decline period (2000–2007/2008) and the steep decline period (2007/2008–2016), Peruvian infants were generally born smaller than international reference populations (HAZ = −0.5); they then experienced rapid postnatal growth faltering from ∼6–24 mo, reaching a nadir at 24 mo. After the peak drop at 24 mo, HAZ reached a plateau from 24 to 59 mo (Figure 3B). In 2007/2008, mean HAZ at birth dropped further into the negatives (HAZ = −0.7). The HAZ birth disadvantage in 2007/2008 disappeared by ∼10 mo until 20 mo when the child growth trajectory again overlapped with that of year 2000. Despite these descriptive trends, 95% CIs of HAZ overlapped from ∼0–24 mo between 2000 and 2007/2008, suggesting that observed differences were not statistically different in these 2 y. Interestingly, however, HAZs of children aged 24–59 mo in 2007/2008 were significantly higher than those of the same cohort in 2000. By 2016, mean child HAZ at birth improved to about −0.6 but remained worse than the −0.5 HAZ in 2000 (although not statistically different). Piecewise linear splines overlaid on the Victora growth curves revealed that the rate of decline in HAZ during the growth faltering period began to slow down over time (Figure 3C, **Supplemental Appendix 5**). In 2000, HAZ was decreasing at a rate of 0.08 SD/mo (95% CI: −0.07, −0.08 SD/mo) between 6 and 20 mo. By 2016, the rate of growth faltering was reduced to 0.03 SD/mo (95% CI: −0.03, −0.03 SD/mo) between 4 and 19 mo (Supplemental Appendix 5).

#### Equity analysis

**FIGURE 4:**
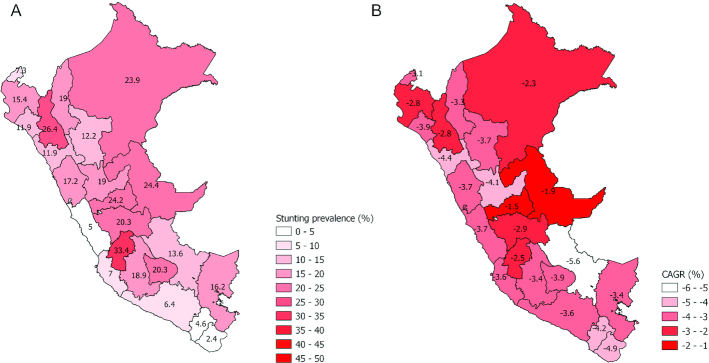
Under-5 stunting estimates by department in Peru. (A) Stunting estimates for children under-5 in Peru, 2000. (B) Stunting estimates for children under-5: compound annual growth rate from 2000 to 2016.

Geospatially, stunting reduction varies dramatically across regions of Peru; generally, a higher stunting prevalence is noted in the Central and Northern (West and East) regions (**Figure 4**). Notable within-department variation also exists; Supplemental Appendix 5 presents the corresponding full maps.

**FIGURE 5:**
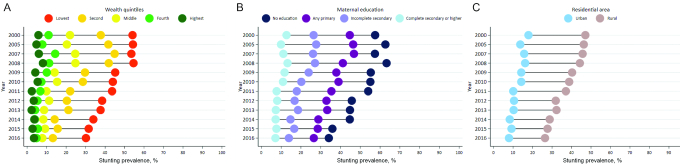
Stunting prevalence disaggregated by socioeconomic and geographic dimensions. (A) Stunting prevalence by wealth quintile, 2000–2016. (B) Stunting prevalence by maternal education, 2000–2016. (C) Stunting prevalence by residential area, 2000–2016. Estimates are based on original Peru Demographic and Health Survey rounds from 2000 to 2016.

Figure 4B shows the compound annual growth rate (CAGR) from 2000 to 2016 for each department. Cusco had the highest CAGR, followed by Huancavelica, Huanuco, and Puno. Conversely, those with the lowest CAGR were Tacna and Tumbes. Of note, Cusco, Huancavelica, Huanuco, and Puno are predominantly Andean regions, whereas Tacna and Lima are coastal ones and predominantly urban. We disaggregated national stunting prevalence into subnational populations and examined reductions in inequalities over time by topography/terrain, wealth quintile, maternal education, urban compared with rural residence, and child sex (**Figures 5**A–C, Supplemental Appendix 5).

Stunting levels are highest in the Amazon and Andes regions and lowest in Lima metropolitan. The prevalence of stunting in Lima and the Andes was 12% and 41%, respectively, in 2000 (Supplemental Appendix 5). By 2016, stunting was reduced to 5% in Lima and to 18% in the Andes. In terms of stunting by wealth quintiles, disparities favoring the rich are pervasive in Peru. Across wealth quintiles, the poorest consistently have the highest levels of stunting, with the prevalence of stunting between the richest and poorest ranging from 8% and 54% in 2000, to 3% and 30% in 2016, respectively (Figure 5A). Nonetheless, children in the lowest and second lowest wealth quintiles have managed to reduce stunting by 24%-points and 25%-points, respectively, from 2000 to 2016, indicating important improvements in this population.

Over time, there was a decrease in the number of children who had mothers without any education (Figure 5B). A dose–response relationship was noted between maternal education and stunting, whereby stunting prevalence was highest among uneducated mothers. Gaps reduced only marginally from 2000 to 2016. In fact, a closing of gaps between the highest prevalence (no education) and lowest prevalence (mothers with higher education) is apparent only after 2011. In 2016, 7% of children from mothers with higher education were stunted, whereas 34% of children from uneducated mothers were stunted.

Stunting prevalence in rural areas was 47% in 2000, 46% in 2007, and 26% in 2016; in urban areas it was 18%, 16%, and 8%, respectively (Figure 5C). The urban–rural gap for under-5 stunting prevalence was 29%-points in 2000 and reduced to 18%-points in 2016. Whereas the absolute gap between urban and rural areas decreased from 2000 to 2016, the relative gap (pro-urban) increased from 2.6 to 3.3.

The difference in stunting prevalence between boys and girls is small in Peru although boys consistently have higher rates of stunting. The largest difference between the sexes was in 2005 (∼7%-points), and the gap narrowed to 2%-points by 2015 and 2016 (Supplemental Appendix 5).

In terms of absolute inequalities, a gradual linear trend in SII is noted from 2000 to 2016, that is, under-5 stunting was more prevalent in the poor in 2000, but became more similar between rich and poor in 2016 (Supplemental Appendix 5). However, the poorest still have on average a 33%-point greater prevalence of stunting than the richest.

As for relative inequalities (CIX), the relative ratio of under-5 stunting was 9.3 times higher in the poorest populations in 2000 and this reduced only slightly to 8.6 times in 2016 (Supplemental Appendix 5).

#### Multivariable analyses

**FIGURE 6:**
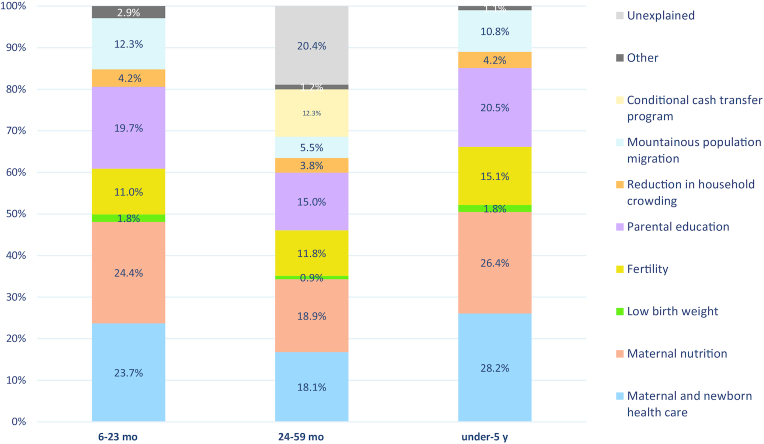
Decomposing predicted changes in HAZ (i.e., percentage contribution of determinant domains) for children under-5 from 2000 to 2016. The <6 mo age category results are not presented owing to small sample sizes and minimal HAZ changes in this population causing unstable parameters. Indicators included maternal and newborn health care (skilled birth attendant and antenatal care ≥4), low birth weight, parental education (maternal and paternal education), mountainous population migration (altitude), maternal nutrition (maternal BMI and height), fertility (parity and interpregnancy interval), reduction in household crowding (number of household members), and conditional cash transfer program (Juntos). “Other” category includes child age, sex, and region for 6- to 23-mo and 24- to 59-mo age groups in addition to economic improvement (wealth index) (0.3%) and childhood vaccines (measles) (0.4%) for the 6- to 23-mo age group, and maternal age (older age pregnancy) (0.5%) and reduction in diarrhea incidence (0.6%) for children under-5. Parental education breakdown: for children 6–23 mo: maternal: 17.5%, paternal: 2.2%; children 24–59 mo: maternal: 13.1%, paternal: 1.9%; and children under-5: maternal: 17.8%, paternal: 2.7%. HAZ, height-for-age *z* score.

**Figure 6** shows the results of decomposing predicted changes in HAZ, i.e., the relative contribution of determinant domains, for different age groups for the period 2000–2016. For children aged 6–23 mo, the most important factors were maternal nutrition (24.4%; proxied by maternal BMI and height), maternal and newborn health care (23.7%), parental education (19.7%), mountainous population migration (12.3%), and fertility (11%). For children aged 24–59 mo the main influencing factors included maternal nutrition (18.9%), maternal and newborn health care (18.1%), parental education (15%), the conditional cash transfer program Juntos (12.3%), and fertility (11.8%), whereas unexplained factors accounted for 20.4% of the whole contribution. For children under-5, the most influential factors included maternal and newborn health care (28.2%), maternal nutrition (26.4%), parental education (20.5%), fertility (15.1%), and migration (10.8%).

Supplemental Appendix 5 shows the decomposition of predicted changes in HAZ, in terms of relative ranking of determinant domains, for children under-5, children between 6 and 23 mo, and children between 24 and 59 mo from 2000 to 2016.

Most distal-, intermediate-, and proximal-level predictors improved over time in Peru (Supplemental Appendix 5). Supplemental Appendix 5 shows in detail the results of the multilevel mixed-effects linear regression. For the period 2000–2016, after adjusting for time and covariables, factors that significantly influenced the reduction of under-5 stunting prevalence included reduction in the percentage of households under the poverty line, increased maternal schooling, younger maternal age, access to the conditional cash transfer program (Juntos), access to the Comprehensive Health Insurance System (SIS), higher Articulated Nutrition Program (PAN) expenditure, increased skilled birth attendant (SBA) use, higher compositive coverage index (CCI), higher pneumococcal vaccine coverage, increased urbanization, reduced open defecation, and reduced acute respiratory infections (ARIs). For the period 2000–2007, time- and covariable-adjusted factors that significantly influenced stunting reduction included higher gross domestic product per capita, lower percentage of households under the poverty line, higher maternal schooling, higher density of human resources for health, urbanization, reduced open defecation, and younger mother's age. CCI was also statistically significantly associated with stunting, but not in the expected direction. For the period 2008–2016, when adjusting for time and confounders, a reduction of households living under the poverty line, increased SBA, increased care seeking for pneumonia, increased urbanization, reduction of ARIs, and reduced parity were significantly associated with stunting reduction over time.

### Policy and program review

**FIGURE 7:**
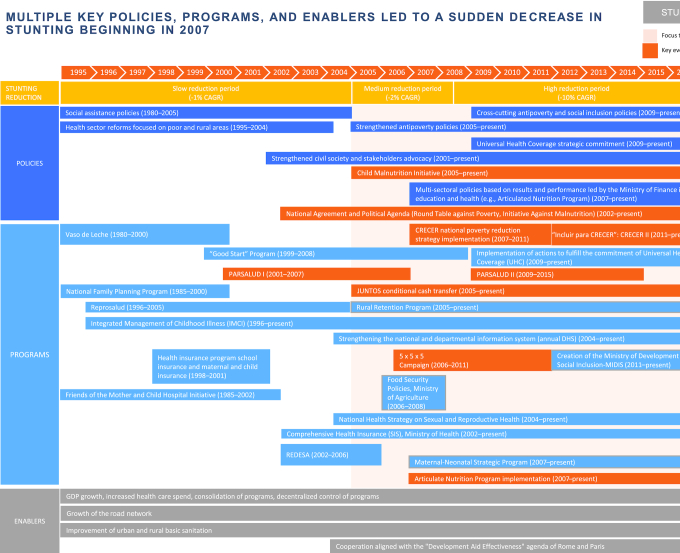
Overview of laws, policies, programs, and enablers between 1995 and 2016 in Peru. CAGR, compound annual growth rate; DHS, Demographic and Health Survey; UHC, Universal Health Coverage.

We present an overview of the laws, policies, programs, and enablers between 1995 and 2016 in Peru in **Figure 7** and **Supplemental Appendix 6**. During the 1980s and the 1990s, feeding programs dominated the efforts to improve child nutrition, such as the Comedores Populares/“Community Kitchens” and Vaso de Leche/“Glass of Milk.” Health reform policies (PARSALUD I and II, the Basic Health For All program, and the Local Committees for Health Administration/CLAS program) were implemented from 1995 to 2004, to strengthen the health system and increase access to essential health services for the most vulnerable populations.

From 2001 onward, as part of the consolidation of democracy, a strong civil society advocacy process occurred through the Roundtable Against Poverty in 2001, the National Agreement in 2002, and the Child Malnutrition Initiative in 2006. These led to specific commitments by political parties to set child stunting reduction as a top priority and to allocate a specific budget line for nutrition. The implementation of multisectoral policies and programs was also initiated. This included the SIS (2002), the conditional cash transfer program (Juntos, 2005), the results-based budgeting programs (e.g., PAN, 2007), and the creation of the Ministry of Development and Social Inclusion to coordinate across sectors.

### Qualitative inquiry results

**Panel 2** provides summary results of the qualitative component of the study and the full detailed qualitative analysis is in **Supplemental Appendix 7**. In addition to the aforementioned improvements in various social determinants of health that occurred in a sustained manner since the early 2000s, stakeholders felt that the paradigm shift from stunting being a feeding problem to one that required preventative and multisectoral action, the strong civil society advocacy, political leadership at all levels with under-5 stunting reduction positioned as a top national political priority, and increased financing of reproductive, maternal, newborn, and child health (RMNCH) were key drivers of stunting reduction from the mid-2000s to 2016.

Panel 2 **Qualitative inquiry results**

Stakeholders stressed the importance of improvements in social determinants, poverty reduction and social safety net programs, commitment to the Millennium Development Goals, focus on preventative nutrition frameworks, and improvements in fertility markers (Supplemental Appendix 7), among other factors. One main factor pointed out was the change in paradigm of the determinants of stunting, from a mere feeding problem that had dominated the design and implementation of programs to fight stunting, to a multisectoral problem that needed to be addressed in an integrated way by different stakeholders and sectors:

“You stop looking at stunting as a feeding problem, and you start considering it as a multifactorial problem, although you don't forget that infections have a role [in the origin of stunting]. Then you start linking the different interventions.” (Participant 8. Technical Advisor, Ministry of Finance and Economy)

Another key driver emphasized was the role of strong civil society advocacy, which paved the way to the consequent political leadership at all levels, with under-5 stunting reduction positioned as a top national political priority, accompanied by increased financing of RMNCH:

“I think that a central factor was the continued dialogue with the political parties, initially through the National Agreement. That occurred at about 2004, 2005. Besides, one of the priority themes within the National Agreement was to work on the rights of children.” (Participant 6. Member, Roundtable Against Poverty)

“There were specific goals [for municipalities to achieve], such as ID for all children and citizens. Contests involving local governments were conducted, and the winner municipalities received the Municipal Seal, along with a President photograph and a plaque.” (Participant 2. Independent Consultant. Former Member, MIDIS)

“The civil society advocacy on childhood issue was very important… they talked directly to the Minister of Economy and Finances … Then we worked together all the process [of implementing policies and programs] with all civil society members.” (Participant 2. Independent Consultant. Former Member, MIDIS)

“Political action was key. The President [of the Republic] himself signed a commitment [to reduce stunting] and made sure to turn it into action, mainly through implementation of the results-based budgeting programs that guaranteed financing, and by convening regional governments.” (Participant 6. Former UNICEF Consultant)

## Discussion

### Summary

Peru achieved a remarkable reduction in the prevalence of stunting among children <5 y of age, from 31.3% in 2000 to 13.1% in 2016, with the greatest reductions occurring during the period of 2008–2016. The gains observed were greatest in the poorest wealth quintile and in rural areas. Consistent with existing literature (105), our mixed-methods comprehensive case study of factors contributing to stunting decline in Peru suggests that a multiplicity of factors focused on improving socioeconomic indicators, reducing inequalities, poverty reduction/social protection, better and affordable access to health care (especially maternal and child health interventions), and broader political and societal enablers were key drivers.

### Strengths and limitations

To our knowledge, our effort is the first aimed at disentangling the role of different factors of the stunting causal chain in Peru through a combination of methodological approaches including a multilevel regression analysis and a decomposition analysis, and encompassing the analysis of an extended period of time at national and subnational levels, by using aggregate and individual data.

The strengths of our study include the use of quantitative approaches to identify the main drivers of stunting using both individual and aggregate data, along with a complementary qualitative component that obtained the insight of individuals familiar with the design and implementation of key policies and programs deliberately addressed to tackle the multidimensionality of stunting. In addition, we took advantage of a conceptual framework that illuminated the identification of the main predictors used in our analyses. This was further strengthened by a unique information system characterizing Peru, which has accumulated an impressive amount of data through DHS surveys carried out regularly over the past 4 decades and which are representative at the national and departmental levels.

We must acknowledge some limitations of our study. First, survey data did not capture aspects related to the quality of maternal, neonatal, and child health care, which is crucial to understand the full potential of different factors that can influence the nutritional status of children. Fortunately, Peru has been incorporating these indicators gradually in their periodic surveys. Second, further disaggregated data are needed to disentangle the role of different factors in stunting reduction more effectively, particularly those operating where implementation of interventions occurs. Such data should be obtained on a routine basis at the local level, and should include administrative data, as well as managerial and governance information, and information related to implementation, monitoring, and evaluation. Third, we could not obtain information on certain variables related more to individual child factors such as prematurity and gestational age, which would have provided information on the status of intrauterine growth in our study population.

### Existing evidence

Our results confirm findings of other studies in low- and middle-income countries, which also showed the need to implement a combination of interventions requiring coordination of different sectors, to achieve a measurable impact on stunting (2, 14, 106–110). Previous studies aimed at identifying drivers of stunting reduction in Peru were either qualitative (10) or quantitative only (16, 111–113).

Our quantitative analyses showed that reduction of poverty, health system strengthening, improved maternal nutrition, higher parental education, reduced fertility, and increased migration to lowlands were strong determinants of stunting reduction.

Our qualitative and policy research found that advocacy and accountability of the civil society were also key factors: particularly, in strengthening political leadership and government's adoption of poverty and stunting reduction as top priorities since the early 2000s. Key enablers that paved the way for increased awareness and political initiatives included the end of the social violence that hit the country in the 1980s and early 1990s; the continued economic growth enjoyed by the country since the late 1990s; the democratic consolidation during the early 2000s; the substantial reduction of under-5 and infant mortality that allowed further focus on childhood nutritional status; and the change that occurred in the conceptual paradigm of the determinants of stunting, from solely a feeding problem, to a crosscutting problem to be addressed by coordinated action by different stakeholders and sectors.

Our analysis of growth trends also suggested that much of the gain in stunting reduction may have occurred via improved nutritional determinants of children younger than 24 mo (particularly 10–24 mo), resulting in gains that then carried over with their usual trajectory to children aged 24–59 mo. Higher stunting prevalence among young children (<24 mo) further emphasizes the importance of interventions focused on the preconception and pregnancy periods, as well as in the early postnatal period.

Regarding the role of the specific antipoverty strategies like Juntos (2005), our analysis found a significant impact on stunting reduction. Previously, 2 cross-sectional studies based on DHS data did not find that Juntos affected child HAZ (113), whereas 1 found a negative association with severe stunting (16). Another longitudinal study found that Juntos participation was associated with increases in HAZ among boys aged 7–8 y who participated for ≥2 y (28).

### Remaining challenges and future research

Peru has to overcome important remaining challenges to consolidate the impressive progress achieved thus far. The overall under-5 stunting prevalence is still higher than those of neighboring countries such as Brazil and Chile. With the rural Amazon and Andes still showing unacceptably high prevalence levels, Peru has to consider tailoring interventions to the particular needs of each of these unique settings. Closing the stunting gap between rural and urban areas, between the richest and the poorest, and between the least and most educated groups is the most formidable challenge for Peru and remains an urgent moral imperative.

We signal a research agenda particularly important for the Peruvian context and for other middle-income countries alike. First, there is the need of further in-depth explorations on the reasons for differing performance at the departmental level in terms of implementation coverage and adequacy of programs and interventions and in terms of the magnitude of stunting reduction. Second, cost-effectiveness analyses of various programs are needed to understand the return on investment. Third, cost-effectiveness analyses of customized interventions for the neediest groups should be undertaken. Fourth, additional research on how Peru's policy agenda differed from those of neighboring countries, particularly those that lagged in stunting prevalence, may reveal useful insights for understanding gaps. Finally, there is the need to conduct implementation research on creative context-specific initiatives that take into account social, economic, geographic, and cultural characteristics of populations that are lagging behind.

### Conclusion

In conclusion, the Peru case is an illustrative example of a country that was able to rapidly reduce its under-5 stunting prevalence after a stagnant period. This is attributed to a combination of diverse factors ranging from effective leadership and strong civil society advocacy to targeting vulnerable populations during the implementation of evidence-based RMNCH interventions. The capacity to take advantage of a positive economic environment characterized by sustained economic growth, the impulse of programmatic initiatives including effective and sustained political leadership, the active participation of civil society in the design and implementation of policies and programs, and the emphasis on adequate accountability mechanisms at all levels, along with the sustained and equitable implementation of out-of-health-sector and within-health-sector evidence-based interventions, constitute valuable lessons derived from the Peruvian experience, which should be taken into account by countries of different regions as part of their efforts to combat child stunting.

## Supplementary Material

nqaa164_Supplemental_File

## Notes

Supported by a Gates Ventures grant to the Centre for Global Child Health (to ZAB) and through a subcontract to Fundación Cayetano Heredia in Peru.

The funder had no role in the design, implementation, analysis, or interpretation of the data.

Supplemental Appendices 1–7 are available from the “Supplementary data” link in the online posting of the article and from the same link in the online table of contents at https://academic.oup.com/ajcn/.

Data described in the article, code book, and analytic code will be made available upon request pending application and approval.

Authors SB to JW organized alphabetically.

Published in a supplement to *The American Journal of Clinical Nutrition*. The Guest Editor for this supplement was Mark Manary, and reports no conflicts of interest. The Supplement Coordinator for the supplement publication was Nadia Akseer, Gates Ventures/Hospital for Sick Children, Toronto, Canada. Supplement Coordinator disclosure: no conflicts of interest. The Stunting Exemplars research Principal Investigator was Zulfiqar A Bhutta, Hospital for Sick Children, Toronto, Canada. Principal Investigator disclosure: no conflicts of interest. Publication costs for this supplement were defrayed in part by the payment of page charges by Gates Ventures. The opinions expressed in this publication are those of the authors and are not attributable to the sponsors or the publisher, Editor, or Editorial Board of *The American Journal of Clinical Nutrition*.

Abbreviations used: ARI, acute respiratory infection; CAGR, compound annual growth rate; CCI, composite coverage index; CIX, concentration index; DHS, Demographic and Health Survey; HAZ, height-for-age *z* score; IMCI, Integrated Management of Childhood Illness; Juntos, conditional cash transfer program; MIDIS, Ministry of Development and Social Inclusion; PAN, Articulated Nutrition Program; RMNCH, reproductive, maternal, newborn, and child health; SBA, skilled birth attendant; SII, slope index of inequality; SIS, Comprehensive Health Insurance System.
