# Corrigendum to: Drivers of stunting reduction in Peru: a country case study. Am J Clin Nutr 2020;112:816S–829S

**DOI:** 10.1093/ajcn/nqaa356

**Published:** 2020-12-10

Corrigendum to: Drivers of stunting reduction in Peru: a country case study. Am J Clin Nutr 2020;112:816S–829S.

In the originally published version of this article, there was an error in the author, Aviva I Rappaport's full name. This has now been corrected online.

