# Supplementary material for: Drivers of stunting reduction in Peru: a country case study
Source: Am J Clin Nutr. 2020 Aug 29;112(Suppl 2):816S–829S. doi: 10.1093/ajcn/nqaa164 (PMC7487430; doi:10.1093/ajcn/nqaa164)
Supplement: nqaa164_Supplemental_File [file nqaa164_supplemental_file.docx]

Drivers of stunting reduction in Peru: a country case study

Authors: Luis Huicho, Elisa Vidal -Cárdenas, Nadia Akseer, Samanpreet Brar, Kaitlin Conway, Muhammad Islam, Elisa Juarez, Aviva Rappaport, Hana Tasic, Tyler Vaivada, Jannah Wigle, Dr. Zulfiqar Bhutta

**Online Supplementary Material**

**List of Appendices**

[**Supplementary Appendix 1:** Descriptive Analysis of Contextual Factors 4](#_Toc40953298)

[**Supplementary Appendix Figure 1A:** Trends in GDP per capita, poverty and urbanization, Peru, 1990-2016 4](#_Toc40953299)

[**Supplementary Appendix Figure 1B:** Trends in key contextual indicators, 1990-2017 4](#_Toc40953300)

[**Supplementary Appendix 2:** Systematic Literature review 5](#_Toc40953301)

[**Supplementary Appendix Figure 2**: Literature review flow diagram 6](#_Toc40953302)

[**Supplementary Appendix 3:** Multivariable Analyses Methods 10](#_Toc40953303)

[**Supplementary Appendix 4:** Qualitative Data Collection and Analyses Methods 13](#_Toc40953304)

[**Supplementary Appendix Figure 3:** Framework proposed by Huicho et al for the determinants of stunting reduction 13](#_Toc40953305)

[**Supplementary Appendix Table 1:** List of participants and their affiliation that were included in the FGD 14](#_Toc40953306)

[**Supplementary Appendix Table 2:** List of participants and their affiliation that were included in the in-depth interviews 15](#_Toc40953307)

[**Supplementary Appendix 5:** Quantitative Results 17](#_Toc40953308)

[**Supplementary Appendix Figure 4A**: Spline analysis of inflection points of change in the slope of HAZ, 2000 17](#_Toc40953309)

[**Supplementary Appendix Figure 4B**: Spline analysis of inflection points of change in the slope of HAZ, 2007/08 17](#_Toc40953310)

[**Supplementary Appendix Figure 4C**: Spline analysis of inflection points of change in the slope of HAZ, 2016 18](#_Toc40953311)

[**Supplementary Appendix Figure 5**: Stunting estimates for children under-5 years old from 2005-2016 19](#_Toc40953312)

[**Supplementary Appendix Figure 6A**: Change in absolute SII by year in Peru 20](#_Toc40953313)

[**Supplementary Appendix Figure 6B**: Change in relative CIX by year in Peru 20](#_Toc40953314)

[**Supplementary Appendix Figure 7A**: Stunting prevalence by gender, 1992/93 – 2017 21](#_Toc40953315)

[**Supplementary Appendix Figure 7B**: Stunting prevalence by geographic región, 2000 – 2016 21](#_Toc40953316)

[**Supplementary Appendix Table 3:** Descriptive trends in stunting determinants in children <5 years 2000, 2007/08, 2016 22](#_Toc40953317)

[**Supplementary Appendix Table 4:** Decomposition analysis for children <5 months from 2000-2016 27](#_Toc40953318)

[**Supplementary Appendix Figure 8:** Decomposing predicted changes in HAZ among children <5 (i.e. relative ranking of product coefficients for determinant domains) from 2000-2016 27](#_Toc40953319)

[**Supplementary Appendix Table 5:** Decomposition analysis for children 6-23 months from 2000-2016 28](#_Toc40953320)

[**Supplementary Appendix Figure 9:** Decomposing predicted changes in HAZ among children 6-23 months (i.e. relative ranking of product coefficients for determinant domains) from 2000-2016 28](#_Toc40953321)

[**Supplementary Appendix Table 6:** Decomposition analysis for children 24-59 months from 2000-2016 29](#_Toc40953322)

[**Supplementary Appendix Figure 10:** Decomposing predicted changes in HAZ among children 24-59 months (i.e. relative ranking of product coefficients for determinant domains) from 2000-2016 29](#_Toc40953323)

[**Supplementary Appendix Table 7:** Decomposition analysis for children <6 months from 2000-2016 30](#_Toc40953324)

[**Supplementary Appendix Figure 11:** Decomposing predicted changes in HAZ among children <6 months (i.e. relative ranking of product coefficients for determinant domains) from 2000-2016 30](#_Toc40953325)

[**Supplementary Appendix Table 8:** Hierarchical linear regression models for children <6 months using individual DHS datasets, period 2000-2016 31](#_Toc40953326)

[**Supplementary Appendix Table 9:** Hierarchical linear regression models for children 6 – 23 months using individual DHS datasets, period 2000-2016 34](#_Toc40953327)

[**Supplementary Appendix Table 10:** Hierarchical linear regression models for children 24 – 59 months using individual DHS datasets, period 2000-2016 39](#_Toc40953328)

[**Supplementary Appendix Table 11:** Multilevel linear models for under-5 stunting prevalence (2 years’ time-lag), period 2000-2016 43](#_Toc40953329)

[**Supplementary Appendix Table 12:** Multilevel linear models for under-5 stunting prevalence (2 years’ time-lag), period 2000-2007 47](#_Toc40953330)

[**Supplementary Appendix Table 13:** Multilevel linear models for under-5 stunting prevalence (2 years’ time-lag), period 2008-2016 51](#_Toc40953331)

[**Supplementary Appendix 6:** Programs and Policies 55](#_Toc40953332)

[**Supplementary Appendix Table 14:** Description of Acts/Law/Regulations, Policies, and Programs from 1995-2016 55](#_Toc40953333)

[**Supplementary Appendix 7:** Qualitative Results 67](#_Toc40953334)

[**Supplementary Appendix References** 77](#_Toc40953335)

# **Supplementary Appendix 1:** Descriptive Analysis of Contextual Factors

## **Supplementary Appendix Figure 1A:** Trends in GDP per capita, poverty and urbanization, Peru, 1990-2016

Source: [1]

## **Supplementary Appendix Figure 1B:** Trends in key contextual indicators, 1990-2017

Source: [1,2]

# **Supplementary Appendix 2:** Systematic Literature review

**Methods**

A systematic search of published peer-reviewed and grey literature related in Peru was undertaken in order to synthesize information on contextual factors, national and subnational interventions, policies, strategies, programs, and initiatives that may have theoretically contributed to reductions in child stunting in Peru over time. Three broad categories of search terms were used: the disease outcome (“stunting”), the population (“child”), and the country of interest (“Peru”). Keywords representing these terms were combined with Boolean operators, adapted with appropriate syntax, and executed in multiple databases. An example of a search syntax is provided below:

1. *Stunting*: "stunting" or "linear growth" or "linear growth stunting" or "HAZ" or "height" or "height-for-age" or "LAZ" or "length" or "length-for-age”
2. *Child*: “child” or “infant”
3. *Peru:* “Peru”
4. 1 AND 2 AND 3

The search for indexed literature was conducted in 15 online databases: MEDLINE, Embase, AMED, CAB Abstracts, CINAHL, Cochrane CENTRAL, Campbell Collaboration, EPPI Centre Trials Register (TRoPHI), 3ie, JOLIS, African Journals Online, WHOLIS, LILACS, Scopus, and Web of Science. Additional searches for grey literature were conducted using Google, a hand search of reference lists of relevant reviews, and direct searching organizational websites, including: the national, regional and headquarter websites for UNICEF, WHO, UNDP, World Bank Group Open Knowledge Repository, Global Alliance for Improved Nutrition, Nutrition International, Nestle Nutrition Institute, International Food Policy Research Institute, and the Government of Peru Ministry of Health (MINSA). The exported set of records were de-duplicated and screened for relevance. Records were included if they met all of the following inclusion criterio and were not restricted by language:

1. included an under-5 population in Peru
2. published between 1990-2017
3. examined one or more of the determinants of chronic undernutrition (e.g. determinants, risk factors, policies, programs, interventions, or initiatives)
4. examined effects on child growth or a reduction in stunting

Initial database searches returned 1120 records, which was reduced to 547 after de-deduplication. Applying the screening criteria to titles and abstracts left 134 records, which were categorized for the purposes of thematic exploration and mapping. Those studies with quantitative analyses are divided into national and subnational levels. At the national level, of primary interest were analyses of determinants of trends or change over time in stunting prevalence. Other studies at the national level examined cross-sectional associations with stunting prevalence at a single point in time. The remaining quantitative studies were conducted at assorted subnational levels, some examining point prevalence, and some following cohorts of children longitudinally. We also categorized qualitative analyses, as well as studies detailing the effects of specific interventions (e.g. randomized controlled trials, or evaluations of programs (e.g. before-after studies). See Figure 2 for a flow diagram outlining the components and progression of the review.

Relevant literature was iteratively synthesized and summarized to inform our research questions and to contrast our findings with existing evidence.

## **Supplementary Appendix Figure 2**: Literature review flow diagram

**

**Full Literature Review**

Factors that have contributed to a reduction in stunting in Peru are presented in relation to an adapted version of UNICEF’s conceptual framework for malnutrition [3]. Originally, policies and programs were aimed at targeting the immediate causes of malnutrition to reduce the country’s high stunting prevalence. These immediate causes, and associated “band-aid” solutions are easier to address than the underlying, and basic causes of stunting at a national level. However, they were unsuccessful partially because they are difficult to implement while targeting the most vulnerable populations [4,5]. After years of ineffective programs, and stagnant stunting rates a roundtable for poverty reduction was created to address the more distal causes of stunting.

The aim of this literature review is to summarize published literature from programs, policies, and intervention studies that targeted basic, underlying, and immediate causes of chronic malnutrition in Peru.

**Basic causes**

Peru experienced rapid economic growth between 2002 and 2010 [6,7]. Galasso and Wagstaff suggest that economic growth accounted for up to 50% of the reduction in stunting nationally [8]. According to the National Institute of Statistics the correlation between poverty and chronic malnutrition is 0.4, this correlation is even lower in rural areas. Similarly, household wealth was positively associated with HAZ [9]. Therefore, rapid economic growth played a role in the reduction of stunting in Peru, but was not the sole contributor [10,11].

Acosta argues that the success in stunting reduction is not a direct result of economic growth because off the disparities in time periods between growth and stunting reduction [6]. Rather, Acosta attributes the reduction in stunting to political efforts, specifically social welfare programs [12].

One of these social welfare programs were JUNTOS, aimed at poverty reduction. The impact of JUNTOS on stunting reduction was evaluated in 6 studies. A total of 3 reported no association [13–15], 2 had a positive association [6,16], and 1 showed reduced early rates of stunting, but had no overall impact at older ages [17]. Many researchers suggest that more time is needed to see the positive impact of JUNTOS on stunting. Another social welfare program was CRECER, a poverty reduction strategy, that targeted the poorest communities [10]. The greatest reductions in stunting were seen in districts that were prioritized by the CRECER program districts [10,18,19]. Despite this, other researchers think that social welfare programs were ineffective and did not contribute to the reduction of stunting in Peru [20].

Differences in stunting prevalence exist between urban and rural areas in Peru [21–25]. Data suggests that the decline in stunting in rural areas was not as steep and started later than it did in urban areas [26,27]. Comparably, smaller children live at higher altitudes in these rural areas (2500 – 3500 m) [28–30]. Studies have found that after controlling for family and child characteristics, there was a significant non-linear relationship between altitude and HAZ [31].

Data suggests that the reduction in stunting is because of strong political commitment [6,10,15,32,33]. For example, the formation of the CMI, and the Poverty Reduction Roundtable put child malnutrition on the national political agenda [10]. In addition, the health insurance program, SIS, increased the accessibility of health care for poor Peruvians [7,10]. JUNTOS also increased the utilization of health services in impoverished Peruvians [34]. Public spending in rural areas increased the probability of poor families accessing health care HAZ in children living in these areas [19,35].

IMCI and Vaccine coverage was significantly correlated with a change in HAZ from 1996 – 2000 [36]. Despite this, there were issues with health center care access. Peru had poor prenatal care attendances compared to other Latin American countries [37]. In addition, many health center staff identified barriers to providing good quality health care, such as insufficient resources, poor infrastructure and poor consistency of message delivery [38,39].

Therefore, the reduction in stunting in Peru is a combination of economic growth, strong political commitment, and social welfare programs targeting poverty reduction in remote areas.

**Underlying causes**

Data from Peru depicts the negative impact of drinking untreated water, poor hygiene and sanitation on HAZ [40,41]. Similarly, children with access to improved water had a lower risk of stunting at 1 and 5 years of age [42]. Contrary, a study that evaluated a home based intervention package to improve safe water access found the intervention group had fewer incidents of diarrhea than the control group, but there was no difference in HAZ [43]. Perhaps the follow up length in this study was too short to see changes in HAZ. Nationally representative data in Peru show improvements in 2005 - 2011 for access to safe water, sanitation and hygiene [6]. Therefore, improved access to water may have played a minor role in the reduction in stunting over this time [6].

The rate of female secondary education enrollment improved in 2005-2011 [6]. The impact of maternal education on HAZ was evaluated in 16 studies. Higher maternal education, or any education was significantly associated with HAZ 14 studies [11,25,30,36,40,44–52], while 2 studies showed no association [53,54]. Caregiver schooling was also associated with HAZ in Peru [55]. Similarly, maternal illiteracy was a risk factor for stunting [56]. The literacy rate in Peru improved between 1993 and 2007 [57]. Therefore, female secondary education may have a minor role in the reduction in stunting over time [6]. This association may be explained by the significant interaction between child feeding practices and maternal education p=0.06 [58].

Two studies evaluated the impact of maternal community organization membership on HAZ. One study found that membership impacts a child’s HAZ in the same way formal education does [59], while another study found no impact [60]. Stunting was not associated with maternal common mental disorders at 1 or 5 years of age after adjusting for possible confounding variables [61,62].

Alternative education programs have a positive impact on chronic malnutrition in Peru. For example, a nutrition specific intervention that targeted improving education on caregiving practices among mothers, improved their feeding practices and the frequency of health care visits and subsequently, HAZ [63,64]. IMCI education among mothers had a positive impact on knowledge, but did not change child’s anthropometry at follow up [65].

The period of 2005 – 2011, had more calories available per capita compared to 1996 – 2005 [6]. Food availability had been linked to changes in HAZ over time in a longitudinal study conducted in urban Peru [66]. However, availability alone will not impact HAZ. Family income influences nutritional status of the children; this mechanism is modified by improved dietary intake [41]. Data from a microcredit study depicts a 15% decrease in food insecurity scores with every loan [67]. Completion of more loan cycles was associated with an increase in mean read meat consumption [67]. The Awajun indigenous people purchased less than 1% of their food, and this population has a high prevalence of stunting [68,69]. Similarly, there is evidence indicating the JUNTOS has a positive impact of consumption of both food and non-food items [34]. Higher household consumption and expenditure is linked to height gain in children between 1 and 5 years [5,55,70,71]. Households that are chronically food insecure had children with significantly lower HAZ compared to households that were food secure [72].

**Immediate causes**

An immediate causes of stunting in Peru is disease and infection. A total of 9 studies evaluated the impact of infection and diarrhea on HAZ. A total of 8 studies found an association between infection and/or diarrhea and HAZ [73–79], while only 1 study had no association [80]. Further, diarrhea, enteroaggregative *Escherichia coli*, STH, and early infancy *Helicobacter pylori* infection were associated with shorter stature [73–79], while Giardia was not [80]. Trials were conducted in Peru to determine the impact of de-worming medications on HAZ in children <5 years. Interventions targeted at reducing parasitic infection in women postpartum and for infants under-2 years of age had no impact on HAZ at 6 months, or at 6 months post medication, respectively[79,81].

Poor dietary intake is another immediate cause of malnutrition. Data from Peru suggests that poor diet is associated with stunting [25]. Changing behavior, specifically dietary intake is challenging, particularly when resources are poor such as is in the Awajun women of Peru [82]. As such, interventions in Peru have been evaluated to improve diet quality, particularly micronutrient intake. Neither pre-natal zinc supplementation or zinc supplements in infants had an impact on HAZ [83–86]. Similarly, multiple micronutrient supplements had no impact on HAZ in studies of different doses and durations [87–90]. Similarly, oligofructose-supplemented infant cereal with and without zinc had no impact on HAZ [91].

There are positive associations between breastfeeding and growth [54,92]. The rate of exclusive breastfeeding increased from 53% in 2000 to 72% in 2013, IYCF practices improved during this time as well [27,93,94]. Increased breastfeeding frequency was negatively associated with growth when children had poor intake of complementary foods and a high prevalence of diarrhea [95,96]. Breastfeeding was positively associated with linear growth in infants with poor intake of complementary foods, specifically, animal foods [97]. Similarly, linear growth was positively associated with intake of complementary foods, specifically, animal foods [97].

Other immediate causes of stunting include low birth weight, maternal height, maternal BMI, and older age. Low birth weight was negatively associated with HAZ [9,24,56,98–100]. Maternal height and maternal BMI were associated with infant height [45,58,99]. A total of 10 studies reported a positive association between stunting and age [11,45,51,54,61,85,96,100–102]. After adjusting for confounding variables, 1 study reported that birth spacing of less than or equal to 24 months was associated with stunting in 1996, 2000, 2010, and 2014 [103].

# **Supplementary Appendix 3:** Multivariable Analyses Methods

We undertook two sets of hierarchical multivariable analyses that are discussed in detail below. Using complementary approaches, each of these analyses attempts to answer the same research question i.e. what are the main predictors of change in child linear growth in Peru during critical time periods? The linear mixed effect regression is an ecological analyses with department-year data and % stunting prevalence as the outcome. However, though it effectively analyzes the entire time series of estimates from 2000-2016, inferences are susceptible to the ecological fallacy. The Oaxaca-Blinder decomposition is based on individual-level data, and thus has larger sample sizes/higher statistical power of estimates, but relies only on DHS survey rounds conducted in 2000, 2007/08 and 2016. Findings from the decomposition, consequently, “ignore” in-between survey rounds and thus any intermittent fluctuations in the predictors. As has been suggested in previous decomposition analyses, we operationalize child HAZ as the linear growth outcome due to its greater statistical efficiency relative to the dichotomous child stunting variable. Each of the two below multivariable methods pose their own strengths and limitations – however, as sensitivity analyses, study inferences should be anchored in both and congruent findings between the methods strengthen the key messages.

**Linear Mixed Effect Regression**

We carried out data analysis for all three time periods. We consider a two-year time lag between predictors and outcome, to allow a reasonable period of time before exploring the effects of the different factors on stunting. It is worth mentioning that, despite Period 1 and 2 having 8 and 9 years of data, respectively, because our analysis considered a two-year time lag between predictors and outcome, the final analysis for each period encompasses 8 years and 7 years, respectively. Therefore, the number of our units of analysis (i.e. department-year) for each time period is 192 (period 1) and 168 (period 2).

We conducted stepwise multilevel mixed-effects linear regression model with department-year as our unit of analysis. This first modelling set was done at departmental level, because the individual modelling, despite theoretical advantages, is strongly driven by individual characteristics, specially poverty. This is not so well measured in surveys and ends up giving way to residual confounding, so that any determinant that also marks poverty ends up as a predictor of stunting. This is the case, for instance, of the percent of population covered by the SIS program, where poorer areas have a higher percentage, and the program ends up a risk factor for stunting, not a preventive factor.

We first ran bivariate correlations between different predictor variables and the outcome of interest at departmental level to examine crude associations. Next, we used a simple variance multilevel model with one variance term for the measures at each time point (level 1) and one variance term for the department level (level 2). This model takes into account the fixed effects of the predictors (structured in our conceptual framework) and also the fixed effects of time. The random effects take into account the variability within departments, over time, as well as between departments. The general formulation of this model is *yij  = ∑ k = 0p βkxk  +  uj  +  eij*, where *yij* is the prevalence of stunting in time *i*, department *j*, the *βs* are the fixed effects for the predictors and *uj* and *eij* are the variability terms between and within departments, respectively.

A hierarchical modelling approach using distal, two intermediate (level 1 and 2), and proximal level variables was executed as suggested by Victora 1997 [104]. Variables within each level were selected from our general conceptual framework as defined in Figure 5. For each level of our conceptual model, starting with the distal level, we first run crude mixed-effects linear regressions, with stunting and one predictor at a time, with year as a fixed term (i.e. variable included in the model irrespective of the selection criteria). We next select those variables with *p*-value ≤ 0.20, irrespective of their direction to move forward for multivariable modeling. Selected variables are entered into backward stepwise elimination modeling within their respective levels and those with *p*-values ≤0.10 are retained.

To obtain the relative contribution across our conceptual framework levels, as well as within each respective level, we performed post-estimation estimations of ‘variance explained’. To estimate the relative contribution across each level, the percentage of residual variance explained by the covariables was calculated as ([residual variance component of null model - residual variance covariable model]/[residual variance null model]), reflecting the proportion of the total variability explained by the covariable model. To estimate the effect size of individual variables within each level, Cohen’s F^2^ (based on R^2^ values of different versions of the multivariable, mixed-effects regression model) was calculated as ([R^2^ of the full model – R^2^ of the restricted model]/[1 - R^2^ of the full model]), with the restricted model excluding the variable to be evaluated (without the effect of interest) and with the random intercept variance constrained to be the same as the full model. Cohen’s F^2^ is an informative, standardized measure (with respect to the total variance) of effect size that allows the evaluation and comparison of local effect sizes between variables within the model as a whole, quantifying the effect as small (F^2^ ≥ 0.02), medium (F^2^ ≥ 0.15), or large (F^2^ ≥ 0.35). All analyses were conducted with Stata 13.1 (Stata Corp., College Station, TX).

**Oaxaca-Blinder Decomposition**

We also undertook the commonly used Oaxaca-Blinder decomposition methods [105] to assess nutritional change over time in Peru. These methods based on individual-level data have increased statistical power and have been widely used to assess nutrition determinants over time in low and middle income settings [106–109].

We analyzed individual-level data from three rounds of Peru’s DHSs: 2000, 2007/08, and 2016. Our analysis focused on the index mother-child pair from each DHS round. Defined as the youngest child of the youngest mother in each household, selection of an index pair simplifies the model and interpretation, and is common practice in advanced analysis of DHS datasets. The total number of index pairs available from each survey were n=8842, n=8278, n=16998 for DHS 2000, 2007/08 and 2016, respectively. A flow chart outlining sample size breakdown during the index pair selection process is presented in Appendix 6. Given that the dietary needs/practices and growth trajectories of children in the first 1000 days of life vary notably from children beyond 2 years of age, it has been suggested that these two cohorts be analyzed separately to unmask true effects of environmental conditions and other factors on undernutrition. We conducted analyses for the entire under-5 child population, and stratified child age groups including <6 months, 6-23 months, and 24+ months as has been proposed in recent literature [110].

We used the continuous formulation of HAZ (as opposed to categorical stunting) as the dependent outcome to strengthen statistical power of the analyses. Linear least square regression models -accounting for survey design and weights - were used to assess associations between $\boldsymbol{y}_{\boldsymbol{i,t}}$, our outcome variable measured for a child *i* at time *t*, a vector of time-varying determinants (**X**), time-invariant child age and sex control variables (**C**), and a survey round time variable (**T**) to capture any trend effects. Collectively, with the standard error term, the model is expressed in Equation 1.

$\boldsymbol{Y}_{\boldsymbol{i,t}}=\boldsymbol{\beta}\boldsymbol{X}_{i,k}+\boldsymbol{C}_{i}+\boldsymbol{T} + \varepsilon_{i,t}$ [Equation 1]

Relevant individual and household level determinants in the DHS dataset were supplemented with department-level variables from the ecological dataset. When similar variables were available in both the ecological and DHS datasets, the latter were selected for analysis. The conceptual framework and corresponding list of covariables, their data sources, and definitions used in decomposition assessment are available in our larger report. Applying the conceptual framework, we used a similar hierarchical modelling approach (as described above) whereby we examined the distal, intermediate (level 1 and 2), and proximal level determinants of HAZ [25].

Equation 1 was applied to derive β coefficients for determinants in 2 time periods (DHS 2007 – DHS 2016, and DHS 2000 – DHS 2016). To explain the relative contribution of each covariable over time to HAZ change, we used the Oaxaca- Blinder decomposition under the assumption that the β coefficients are the same across the two populations and the error term has the mean zero. Using the estimated parameters from Equation 1 and the (weighted) means of explanatory variables in the two time points, we applied Equation 2 (e.g. for years 2000 to 2016) to obtain the predicted change in HAZ due to the change in each determinant [26].

$$\Delta\bar{Y}_{i,t}=\beta\left( \bar{X}_{2016}-\bar{X}_{2000} \right)$$

The product coefficients for individual determinants were subsequently ranked to identify the relative contribution of each factor to HAZ change. Like determinants were also grouped into broader domains for interpretation. We examined variance inflation factors (VIF) to assess multicollinearity between variables whereby a VIF > 3 was considered suspect of high inter-variable correlation. For model building, a p-value <0.20 was considered statistically important and variables with p< 0.10 were retained in the final hierarchical multivariable models. All analyses were carried out in Stata version 14.0.

# **Supplementary Appendix 4:** Qualitative Data Collection and Analyses Methods

**Qualitative Inquiry Process**

To understand national stakeholder perspectives on Peru’s nutrition evolution (focused on progress in stunting) and the major contributing factors behind it – we conducted a focus group discussion (FGD) followed by a series of in-depth interviews with key stakeholders. Detail on our methods and results can be found in the complete **qualitative report** submitted as a supplement to this summary evaluation. Below and throughout this summary report, we provide a synopsis of key methods and results.

Semi-structured discussion/interview guides were used for the focus group discussion and interviews and were based on the framework proposed by Huicho et al. [7] (Figure 3). This framework is akin to the UNICEF nutrition determinants framework and the one presented in the Lancet nutrition series – both which were used to draft our adapted study conceptual framework – but highlights more broadly the contextual, policy and program, and service quality/availability factors. Given the nature of policy and programmatic information to be collected from prospective participants, this slightly modified framework was adopted to focus insight on broad contextual drivers and sector-related changes in Peru (e.g. in health sector vs others) that participants could readily relate to.


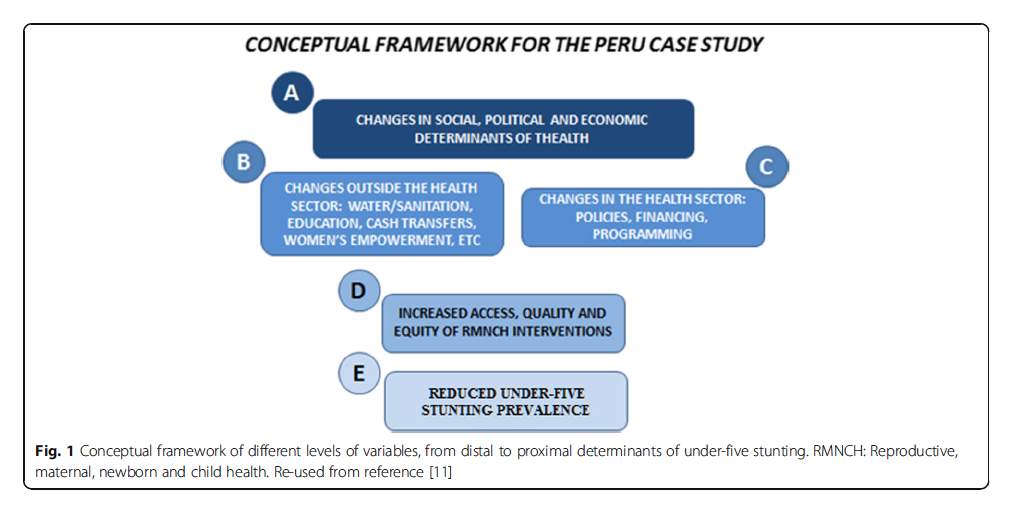


## **Supplementary Appendix Figure 3:** Framework proposed by Huicho et al for the determinants of stunting reduction

**Data Collection Methods**

Both activities were conducted from August 14 to September 30, 2017. The FGD was performed first, followed by the in-depth interviews, to identify through the group discussions specific points needing further insight through individual interviews, so as to get a more balanced perspective of all participants. We aimed not to repeat participants in each of the activities.

**Focus Group Discussions**

The group discussion was conducted in a neutral setting and lasted approximately 100 minutes. The FGD participants were selected among recognized representatives of institutions considered key in the efforts to reduce child stunting in Peru. Six participants were selected and attended this activity, which was conducted by two members of Peru research team (Elisa Juárez and Elisa Vidal). They were current or former representatives from different stakeholders, key in the implementation of policies programs deemed relevant in the efforts for stunting reduction, including MINSA, Ministry of finance and economy (MoF), Ministry of Development and Social Inclusion (MIDIS), The World Bank, RoundTable Against Poverty, CARE Peru), the Pan American Health Organization, and UNICEF. A full list of participants and their affiliation for the FGD are included in Table 1 below.

## **Supplementary Appendix Table 1:** List of participants and their affiliation that were included in the FGD

| Participant | Position & affiliation |
| --- | --- |
| **Focus group discussion participants** | |
| Participant 1 | Member of the technical team, Direction of Health Promotion of the Ministry of Health, Strategy of Healthy Feeding and Nutrition. |
| Participant 2 | Medical doctor, specialist in nutrition and public. Institution: General Direction of Monitoring and Evaluation, Ministry of Development and Social Inclusion. |
| Participant 3 | Sociologist. Member, Technical Team of the Roundtable of Fight Against Poverty. |
| Participant 4 | Medical doctor, specialist in public health and in monitoring and evaluation. Direction of Monitoring and Evaluation, Ministry of Development and Social Inclusion |
| Participant 5 | Nurse, specialist in public health. Coordinator, Nutrition and Child Development, MIDIS. |
| Participant 6 | Pediatrician, former technical officer of UNICEF and advisor in child health and nutrition for the Ministry of Health. Currently, independent advisor to the United Nations World Food Program. |

In-Depth Interviews

The in-depth interviews were conducted after the FGD, allowing us to explore in further detail aspects that were highlighted during the group discussion. We conducted 10 interviews to key informants, selected on the basis of their experience in areas or institutions that were instrumental in the design and implementation of interventions to reduce child stunting in Peru, including the MoF, the Ministry of Health, the MIDIS; the National Institute of Statistics and Computing; UNICEF, the Roundtable Against Poverty, the Pan American Health Organization, CARE Peru, The World Bank. Given time and logistic constraints, most of the individual interviews were conducted through interactive Skype sessions, except for one face-to-face interview. A full list of participants and their affiliation for the in-depth interviews are included in Table 2.

## **Supplementary Appendix Table 2:** List of participants and their affiliation that were included in the in-depth interviews

| Participant | Position & affiliation |
| --- | --- |
| **In-depth interviews participants** | |
| Participant 1 | Member of Faculty of Universidad Peruana Cayetano Heredia since 1990. Technical officer at the Ministry of Economy and Finances since 2007. Specialist in the implementation of Results-Based Budgeting Programs. |
| Participant 2 | Medical doctor and public health expert. Former member of technical teams at the Ministry of Economy and Finances, Ministry of Health, and Ministry of Development and Social Inclusion. Former Vice-Minister of Policies and Social Evaluation at MIDIS. |
| Participant 3 | Former technical officer at the Ministry of Health. Key member of the team that developed the causal conceptual framework for child stunting. Former member of technical team of the Ministry of Economy and Finances that implemented the Results-Based Budgeting Programs (*Presupuesto por Resultados, PPR*). Experience in design and implementation of two PPR programs: the Articulated Nutrition Program and the Strategic Maternal-Neonatal Program. |
| Participant 4 | Specialist in Public Nutrition. Member of the Ministry of Economy and Finances. Former technical member of the National Institute of Statistics and Computing (*Instituto Nacional de Estadística e Informática, INEI*). Former member of UNICEF, specifically involved in aspects related to child stunting. |
| Participant 5 | Pediatrician. Former technical officer of UNICEF and advisor in child health and nutrition for the Ministry of Health. Currently, independent advisor to the United Nations World Food Program.^1^ |
| Participant 6 | Anthropologist. Expert in human rights and policy sciences. Member of the Roundtable Against Poverty, where they coordinate social themes, particularly those related to maternal, neonatal and child health and nutrition. |
| Participant 7 | Medical doctor and public health specialist, eexperience in primary health care. Formerly member of the International Cooperation Office at the Ministry of Health. From 2007 onwards technical officer at the Pan American Health Organization, where they participate in initiatives related to nutrition, such as the launch of the Pan American Alliance Against Malnutrition |
| Participant 8 | Medical doctor. Specialist in Health Administration, social policies, public health and finances. Member of the Ministry of Economy and Finances since 2009, coordinating budgetary support, particularly to programs related to childhood and maternal and child health. From 2009 to 2012 provided support to regional governments and to the Ministry of Health in the costing process of products related to the Articulated Nutrition Program and the Strategic Maternal-Neonatal Program. Former member of the health reform program PARSALUD II. Program of Complementary Feeding for High Risk Groups (*Programa de Complementación Alimentaria para Grupos en Mayor Riesgo, Programa de Distribución de Papillas*). |
| Participant 9 | Economist, Master in Education and Social Work. Current National Director of Care. Member of CARE for 27 years. Coordinator of the Child Malnutrition Initiative (*Iniciativa contra la Desnutrición Infantil, IDI*) since its launch in 2006. |
| Participant 10 | National specialist of Social Protection of the World Bank in Peru. In charge of supervision and technical follow-up during implementation of programs focused on closing the gaps of stunting. |

*Notes:* ^1^This participant was also part of the focus group

**Qualitative Data Analysis**

We performed a deductive analysis, using a data discharge matrix where we incorporated the information considered relevant for answering our research question. The analysis of the FGD was performed by two coding persons simultaneously. This allowed the exchange of ideas on the information obtained and the codes to employ. The analysis of the in-depth interviews was performed by one of the coding persons. The main objective of this task was to explore in further detail the role of each institution previously identified as key in the implementation of efforts against stunting, as well as to explore in further detail facilitating factors and barriers in the efforts of stunting reduction. The analysis also tried to identify the remaining challenges that need to be taken into consideration in future efforts against stunting. A third person (the PI of the Peru team) checked the in-depth interviews and the FGD transcriptions to refine the above described analyses.

# **Supplementary Appendix 5:** Quantitative Results

## **Supplementary Appendix Figure 4A**: Spline analysis of inflection points of change in the slope of HAZ, 2000


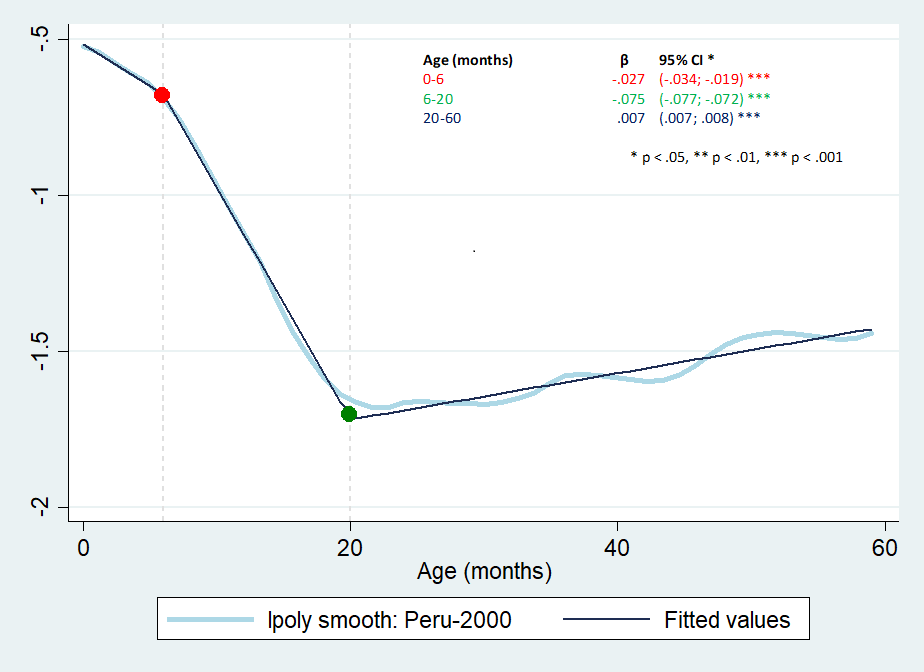


## **Supplementary Appendix Figure 4B**: Spline analysis of inflection points of change in the slope of HAZ, 2007/08


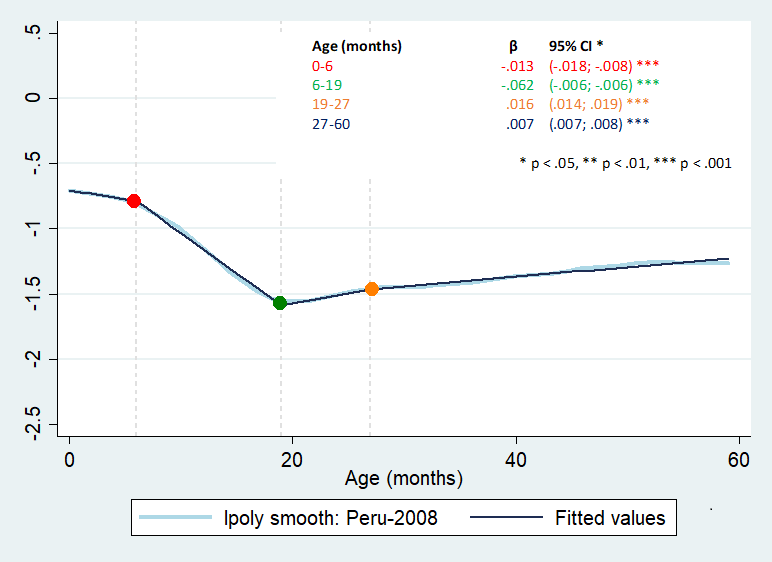


## **Supplementary Appendix Figure 4C**: Spline analysis of inflection points of change in the slope of HAZ, 2016


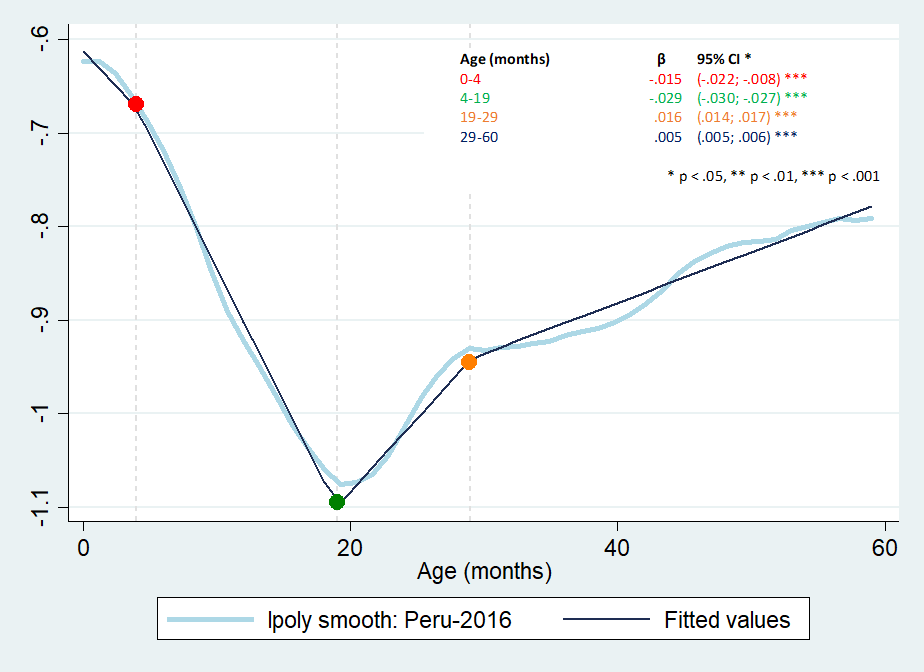


## **Supplementary Appendix Figure 5**: Stunting estimates for children under-5 years old from 2005-2016


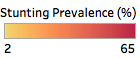

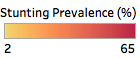

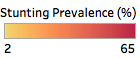

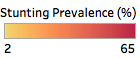

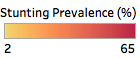

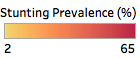

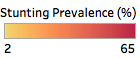

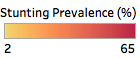

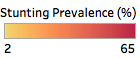

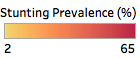

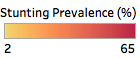

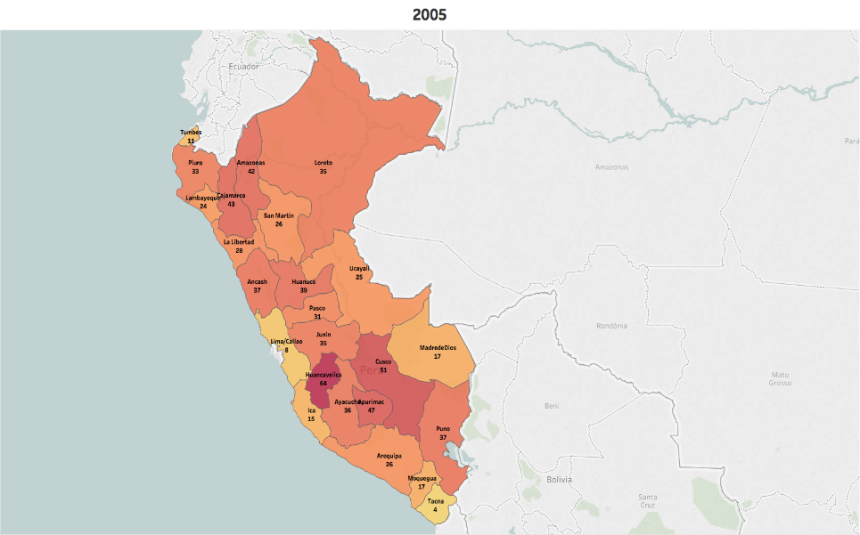

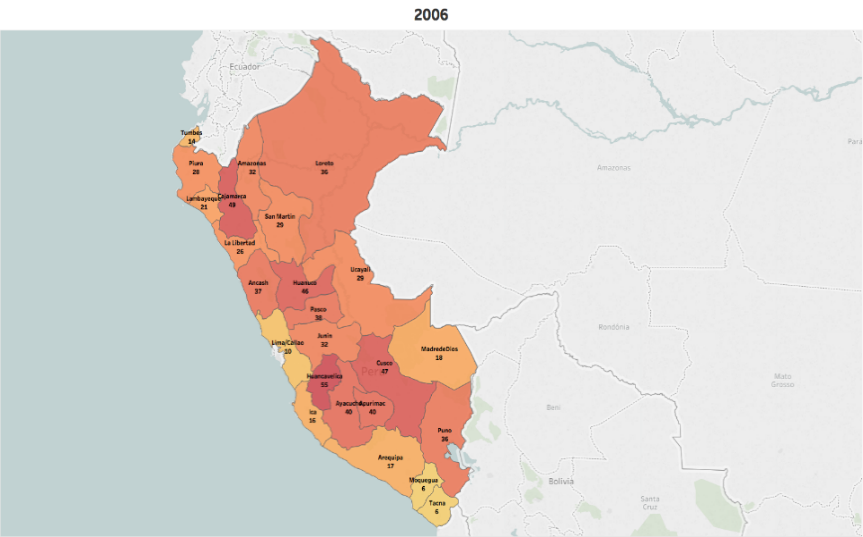

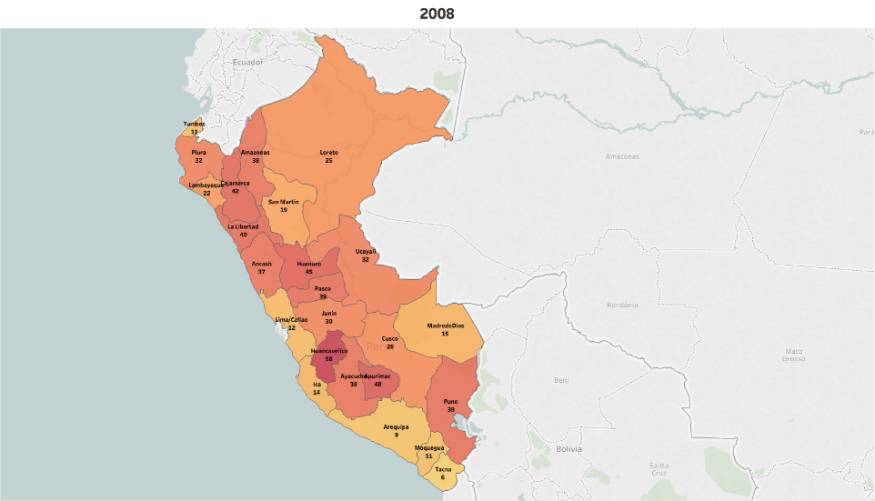

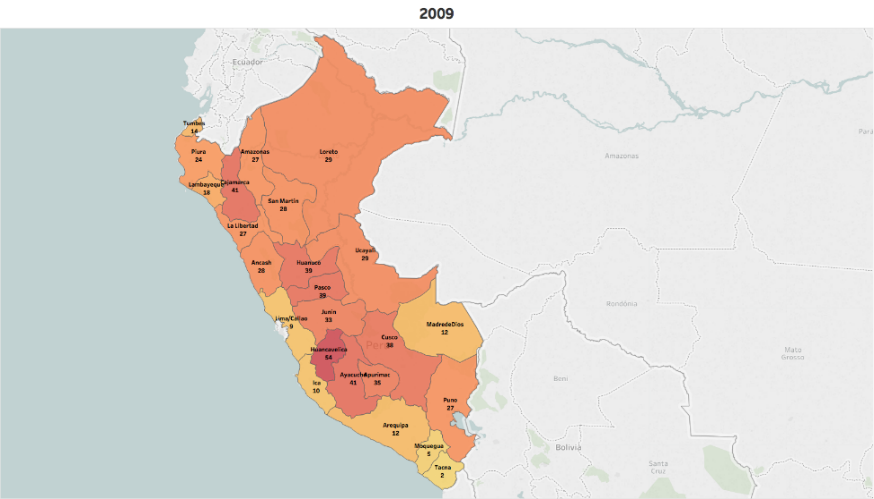

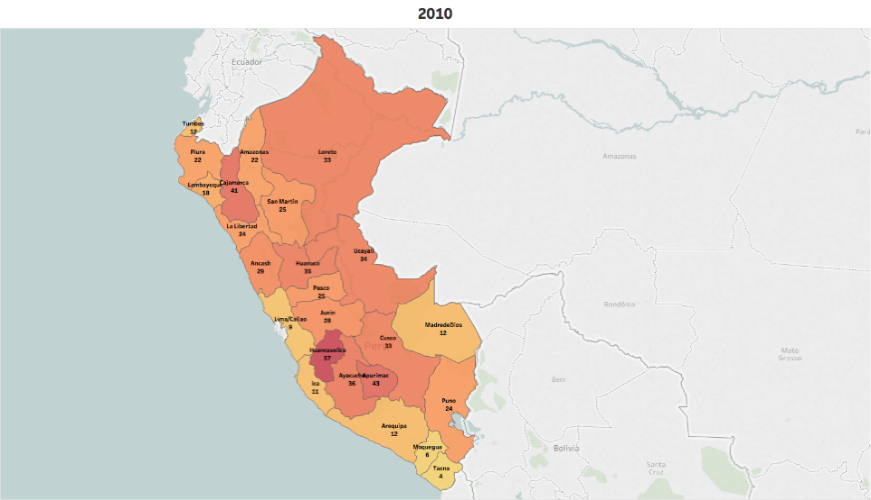

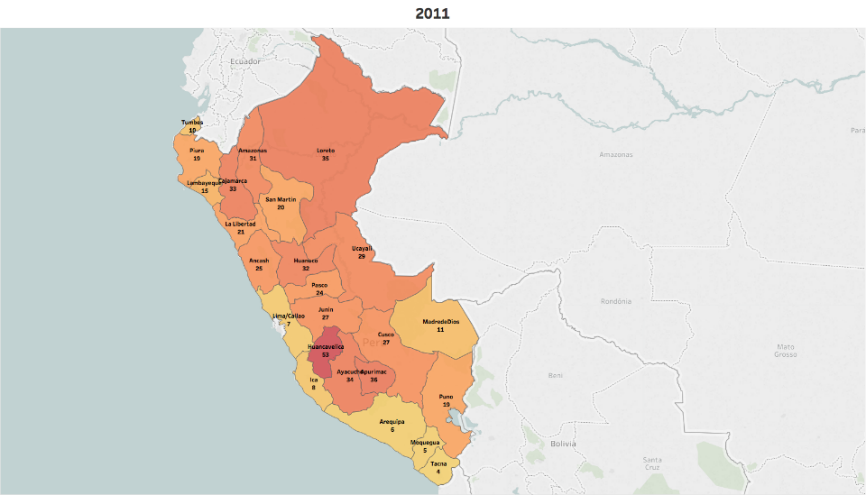

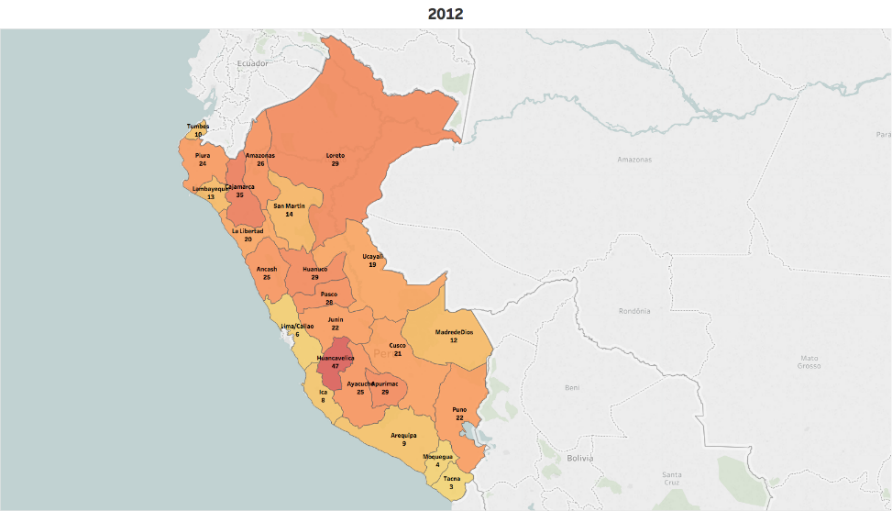

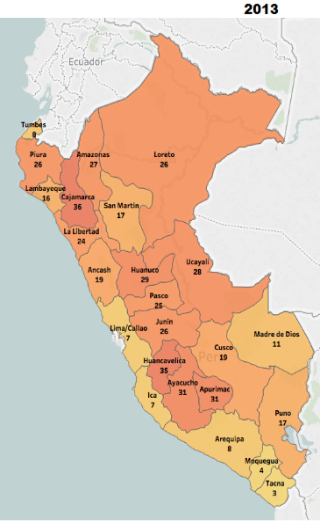

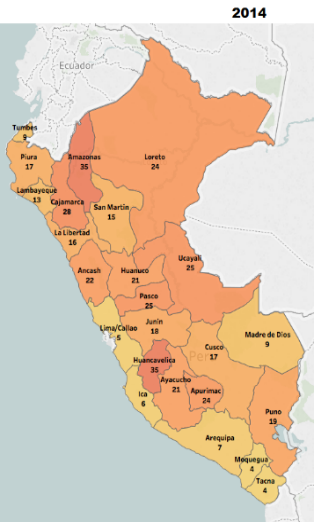

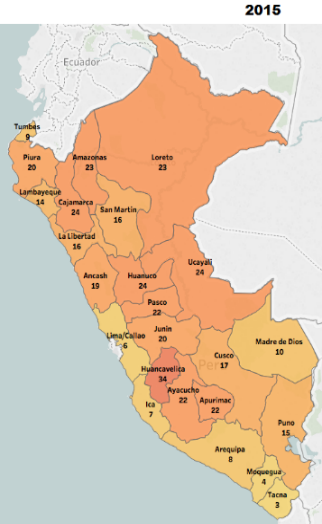

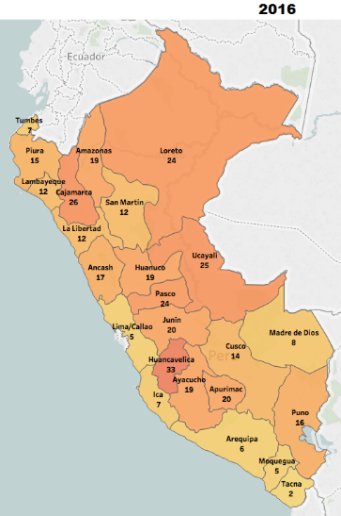


zzz

## **Supplementary Appendix Figure 6A**: Change in absolute SII by year in Peru

## **Supplementary Appendix Figure 6B**: Change in relative CIX by year in Peru

## **Supplementary Appendix Figure 7A**: Stunting prevalence by gender, 1992/93 – 2017


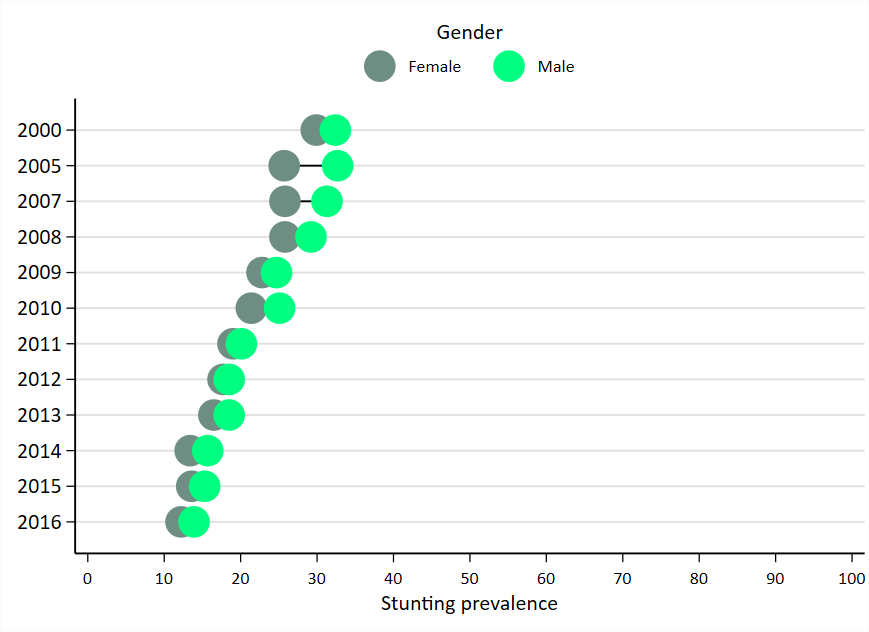


## **Supplementary Appendix Figure 7B**: Stunting prevalence by geographic región, 2000 – 2016


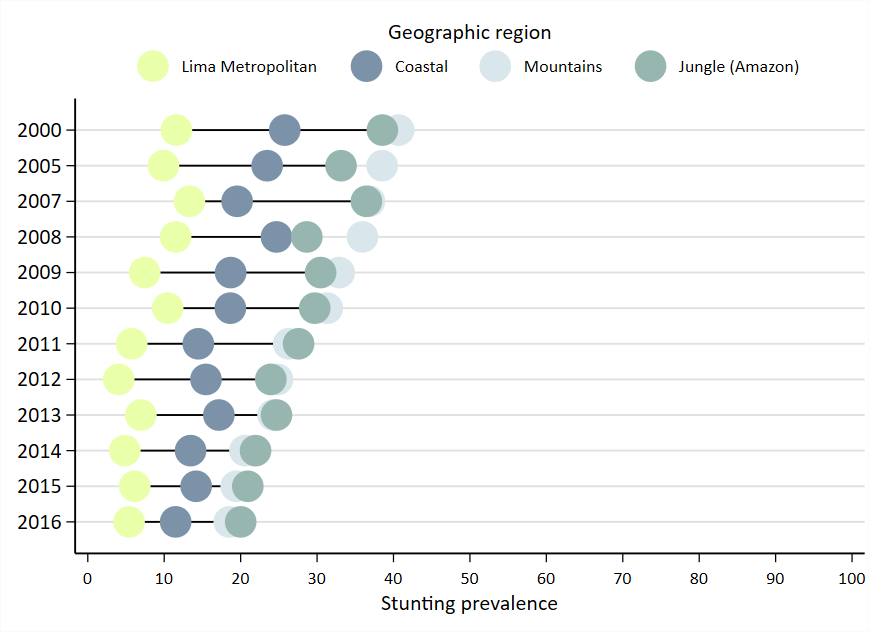


## **Supplementary Appendix Table 3:** Descriptive trends in stunting determinants in children <5 years 2000, 2007/08, 2016

| **Domain/Indicator** | **DHS Survey Year** | | | | | | | | |
| --- | --- | --- | --- | --- | --- | --- | --- | --- | --- |
|  | **2000** | **2007-08** | **2016** | **Earlier time period (2007-08 - 2000)** | | **Later time period (2016 - 2007-08 )** | | **Full time period (2016 - 2000)** | |
|  | **(n = 8848)** | **(n = 8289)** | **(n =16999)** | **Change** | **p-value** | **Change** | **p-value** | **Change** | **p-value** |
| **Outcome** | | | | | | | | | |
| Height for age z-score | -1.24 | -1.21 | -0.84 | 0.03 | 0.283 | 0.37 | <0.001 | 0.40 | <0.001 |
|  |  |  |  |  |  |  |  |  |  |
|  |  |  |  |  |  |  |  |  |  |
| Stunting | 27.59 | 25.51 | 11.90 | -2.08 | 0.035 | -13.65 | <0.001 | -15.73 | <0.001 |
| % of children below -2 SD |  |  |  |  |  |  |  |  |  |
|  |  |  |  |  |  |  |  |  |  |
| **Child Demographic** | | | | | | | | | |
|  | | | | | | | | | |
| Child sex (male) | 51.06 | 50.17 | 51.70 | -0.89 | 0.374 | 1.53 | 0.088 | 0.64 | 0.458 |
| % of males |  |  |  |  |  |  |  |  |  |
|  |  |  |  |  |  |  |  |  |  |
| Child age | 26.91 | 27.09 | 28.16 | 0.18 | 0.610 | 1.07 | 0.001 | 1.25 | <0.001 |
| (in months) |  |  |  |  |  |  |  |  |  |
| **Distal level** | | | | | | | | | |
| **Basic causes & Income poverty** | | | | | | | | | |
| Wealth Index | 5.70 | 5.72 | 5.47 | 0.02 | 0.856 | -0.25 | 0.010 | -0.23 | 0.007 |
| (0 - 10) |  |  |  |  |  |  |  |  |  |
|  |  |  |  |  |  |  |  |  |  |
| Log GDP, per capita | 6.90 | 8.02 | 8.33 | 1.12 | <0.001 | 0.31 | <0.001 | 1.43 | <0.001 |
| (USD/person) |  |  |  |  |  |  |  |  |  |
|  |  |  |  |  |  |  |  |  |  |
| Poverty line | 49.64 | 40.32 | 21.19 | -9.32 | <0.001 | -19.13 | <0.001 | -28.45 | <0.001 |
| (% families below) |  |  |  |  |  |  |  |  |  |
|  |  |  |  |  |  |  |  |  |  |
| Gini coefficient (for income) | 62.97 | 60.42 | 53.73 | -2.55 | <0.001 | -6.69 | <0.001 | -9.24 | <0.001 |
| 0 (perfect equality) – 1 (perfect inequality) |  |  |  |  |  |  |  |  |  |
|  |  |  |  |  |  |  |  |  |  |
| Maternal years of schooling | 8.02 | 8.66 | 10.14 | 0.64 | <0.001 | 1.48 | <0.001 | 2.13 | <0.001 |
|  |  |  |  |  |  |  |  |  |  |
|  |  |  |  |  |  |  |  |  |  |
| Paternal years of schooling | 9.27 | 9.68 | 9.84 | 0.41 | <0.001 | 0.16 | 0.100 | 0.57 | <0.001 |
|  |  |  |  |  |  |  |  |  |  |
|  |  |  |  |  |  |  |  |  |  |
| **Intermediate level - I** | | | | | | | | | |
| **Nutrition Specific and Sensitive Programs** | | | | | | | | | |
| CCT “Juntos” program | 0.00 | 36.82 | 60.21 | 36.82 | <0.001 | 23.40 | <0.001 | 60.21 | <0.001 |
| (No. families/1000 rural population) |  |  |  |  |  |  |  |  |  |
|  |  |  |  |  |  |  |  |  |  |
| Comprehensive Health Insurance (SIS coverage) | 0.00 | 68.98 | 73.73 | 68.98 | <0.001 | 4.75 | <0.001 | 73.73 | <0.001 |
| (No. under-5 attendances/under-5 population) |  |  |  |  |  |  |  |  |  |
|  |  |  |  |  |  |  |  |  |  |
| Articulated Nutrition Program (PANlog) expenditure | 0.00 | 0.00 | 5.29 | 0.00 | <0.001 | 5.29 | <0.001 | 5.29 | <0.001 |
| (USD/person) |  |  |  |  |  |  |  |  |  |
|  |  |  |  |  |  |  |  |  |  |
| Strategic Program of Maternal and Newborn Health (PSMN log expenditure), per capita | 0.00 | 0.00 | 6.96 | 0.00 | <0.001 | 6.96 | <0.001 | 6.96 | <0.001 |
| (USD/person) |  |  |  |  |  |  |  |  |  |
|  |  |  |  |  |  |  |  |  |  |
| **Intermediate level - II** | | | | | | | | | |
| **Inadequate care and feeding practices** | | | | | | | | | |
| Duration of breast feeding | 15.12 | 15.04 | 15.67 | -0.09 | 0.668 | 0.63 | <0.001 | 0.55 | 0.001 |
| (in months) |  |  |  |  |  |  |  |  |  |
|  |  |  |  |  |  |  |  |  |  |
| **Food insecurity** | | | | | | | | | |
| Altitude  (m) | 1389.84 | 1406.35 | 1080.14 | 16.51 | 0.760 | -326.21 | <0.001 | -309.70 | <0.001 |
|  |  |  |  |  |  |  |  |  |  |
|  |  |  |  |  |  |  |  |  |  |
| **Childhood vaccines** | | | | | | | | | |
| DPT vaccine | 91.98 | 93.04 | 92.80 | 1.06 | 0.060 | -0.26 | 0.61 | 0.80 | 0.097 |
| (% infants with 3 doses)  (index child) |  |  |  |  |  |  |  |  |  |
|  |  |  |  |  |  |  |  |  |  |
| Measles vaccine | 67.32 | 65.33 | 70.50 | -1.99 | 0.042 | 5.17 | <0.001 | 3.19 | <0.001 |
| (% infants)  (index child) |  |  |  |  |  |  |  |  |  |
|  |  |  |  |  |  |  |  |  |  |
| Pneumococcal vaccine | 0.00 | 0.00 | 82.90 | 0.00 | <0.001 | 82.90 | <0.001 | 82.90 | <0.001 |
| (% infants with vaccine during first 1 year) |  |  |  |  |  |  |  |  |  |
|  |  |  |  |  |  |  |  |  |  |
| Rotavirus vaccine | 0.00 | 0.00 | 81.27 | 0.00 | <0.001 | 81.27 | <0.001 | 81.27 | <0.001 |
| (% infants with vaccine during first 1 year) |  |  |  |  |  |  |  |  |  |
|  |  |  |  |  |  |  |  |  |  |
| **Reproductive and maternal care** | | | | | | | | | |
| Skilled attendant at birth | 51.45 | 80.35 | 93.80 | 28.90 | <0.001 | 13.49 | <0.001 | 42.39 | <0.001 |
| (index child) |  |  |  |  |  |  |  |  |  |
|  |  |  |  |  |  |  |  |  |  |
| 4+ antenatal care visits | 69.67 | 91.28 | 96.50 | 21.61 | <0.001 | 5.18 | <0.001 | 26.79 | <0.001 |
| (index child) |  |  |  |  |  |  |  |  |  |
|  |  |  |  |  |  |  |  |  |  |
| Family planning services | 87.06 | 89.65 | 92.38 | 2.59 | <0.001 | 2.73 | <0.001 | 5.32 | <0.001 |
| (% women with demand for FP satisfied) |  |  |  |  |  |  |  |  |  |
|  |  |  |  |  |  |  |  |  |  |
| **Human resources for health** | | | | | | | | | |
| Density of human resources for health | 9.04 | 11.78 | 28.23 | 2.74 | <0.001 | 16.45 | <0.001 | 19.19 | <0.001 |
| (Doctors, nurses and midwives per 10,000 |  |  |  |  |  |  |  |  |  |
| population) |  |  |  |  |  |  |  |  |  |
| **Care seeking interventions for childhood illness & child micronutrient intake** | | | | | | | | | |
| Oral rehydration therapy | 46.18 | 60.14 | 61.64 | 13.95 | <0.001 | 1.50 | <0.001 | 15.45 | <0.001 |
| (% children with diarrhea receiving ORT) |  |  |  |  |  |  |  |  |  |
|  |  |  |  |  |  |  |  |  |  |
| Care seeking for pneumonia | 58.01 | 73.22 | 68.12 | 15.21 | <0.001 | -5.10 | <0.001 | 10.11 | <0.001 |
| (% children with pneumonia seeking care) |  |  |  |  |  |  |  |  |  |
|  |  |  |  |  |  |  |  |  |  |
| Vitamin A supplementation | 3.65 | 5.25 | 4.98 | 1.60 | <0.001 | -0.27 | 0.07 | 1.33 | <0.001 |
| (index child) |  |  |  |  |  |  |  |  |  |
|  |  |  |  |  |  |  |  |  |  |
| **Unhealthy household environment** | | | | | | | | | |
| Urban locality | 57.92 | 60.59 | 73.60 | 2.67 | 0.055 | 13.05 | <0.001 | 15.73 | <0.001 |
| (index household) |  |  |  |  |  |  |  |  |  |
|  |  |  |  |  |  |  |  |  |  |
| Open defecation | 25.55 | 14.98 | 8.10 | -10.57 | <0.001 | -6.85 | <0.001 | -17.42 | <0.001 |
| (index household) |  |  |  |  |  |  |  |  |  |
|  |  |  |  |  |  |  |  |  |  |
| Access to improved drinking water sources | 82.02 | 83.52 | 89.70 | 1.50 | 0.251 | 6.18 | <0.001 | 7.67 | <0.001 |
| (index household) |  |  |  |  |  |  |  |  |  |
|  |  |  |  |  |  |  |  |  |  |
| Number of household members  (index household) | 5.94 | 5.50 | 5.07 | -0.44 | <0.001 | -0.43 | <0.001 | -0.87 | <0.001 |
|  |  |  |  |  |  |  |  |  |  |
|  |  |  |  |  |  |  |  |  |  |
| **Proximal level** | | | | | | | | | |
| **Infections** | | | | | | | | | |
| ARI infection | 45.56 | 42.80 | 36.40 | -2.75 | 0.016 | -6.43 | <0.001 | -9.18 | <0.001 |
| (index child within last 2 weeks) |  |  |  |  |  |  |  |  |  |
|  |  |  |  |  |  |  |  |  |  |
| Diarrhea infection | 16.87 | 15.53 | 12.20 | -1.34 | 0.088 | -3.30 | <0.001 | -4.64 | <0.001 |
| (index child within last 2 weeks) |  |  |  |  |  |  |  |  |  |
|  |  |  |  |  |  |  |  |  |  |
| **Child characteristics** | | | | | | | | | |
| Low birthweight | 8.20 | 6.93 | 6.30 | -1.27 | 0.036 | -0.63 | 0.192 | -1.90 | <0.001 |
| (index child) |  |  |  |  |  |  |  |  |  |
|  |  |  |  |  |  |  |  |  |  |
| **Maternal characteristics** | | | | | | | | | |
| Maternal age | 29.39 | 29.52 | 30.19 | 0.13 | 0.415 | 0.67 | <0.001 | 0.80 | <0.001 |
| (years) |  |  |  |  |  |  |  |  |  |
|  |  |  |  |  |  |  |  |  |  |
| Adolescent birth (<18 years of age) | 5.52 | 6.11 | 5.20 | 0.59 | 0.212 | -0.92 | 0.040 | -0.33 | 0.343 |
| (current birth for index mother) |  |  |  |  |  |  |  |  |  |
|  |  |  |  |  |  |  |  |  |  |
| Older mother birth (≥35 years) | 16.34 | 17.44 | 19.00 | 1.10 | 0.171 | 1.60 | 0.032 | 2.69 | <0.001 |
| (current birth for index mother) |  |  |  |  |  |  |  |  |  |
|  |  |  |  |  |  |  |  |  |  |
| Anemia during pregnancy | 35.07 | 26.55 | 21.50 | -8.52 | <0.001 | -5.02 | <0.001 | -13.54 | <0.001 |
| (current birth for index mother) |  |  |  |  |  |  |  |  |  |
|  |  |  |  |  |  |  |  |  |  |
| Body mass index (kg/m^2^) | 25.48 | 25.57 | 26.98 | 0.09 | 0.336 | 1.41 | <0.001 | 1.51 | <0.001 |
| (index mother) |  |  |  |  |  |  |  |  |  |
|  |  |  |  |  |  |  |  |  |  |
| Height (cm) | 150.47 | 151.18 | 152.26 | 0.72 | <0.001 | 1.08 | <0.001 | 1.79 | <0.001 |
| (index mother) |  |  |  |  |  |  |  |  |  |
|  |  |  |  |  |  |  |  |  |  |
| Parity (No. children) | 3.04 | 2.73 | 2.39 | -0.31 | <0.001 | -0.34 | <0.001 | -0.65 | <0.001 |
| (index mother) |  |  |  |  |  |  |  |  |  |
|  |  |  |  |  |  |  |  |  |  |
| Interpregnancy interval (months) | 50.18 | 58.49 | 67.99 | 8.31 | <0.001 | 9.50 | <0.001 | 17.81 | <0.001 |
| (index mother) |  |  |  |  |  |  |  |  |  |
|  |  |  |  |  |  |  |  |  |  |

## **Supplementary Appendix Table 4:** Decomposition analysis for children <5 months from 2000-2016

| **Factors** | **Estimated**  **coefficient** | **Mean difference (2016-2000)** | **Predicted change in HAZ** | **Share of predicted change in (%)** |
| --- | --- | --- | --- | --- |
|  |  | 0.400 | 0.435 | 108.8% |
| Wealth Index | 0.082 | -0.230 | -0.019 | -4.3% |
| Mother years of schooling | 0.036 | 2.125 | 0.077 | 17.8% |
| Father years of schooling | 0.020 | 0.572 | 0.012 | 2.7% |
| Duration of Breastfeed (months) | -0.013 | 0.547 | -0.007 | -1.6% |
| Altitude | 0.000 | -309.703 | 0.047 | 10.8% |
| DPT vaccine | -0.129 | 0.008 | -0.001 | -0.2% |
| Measles vaccine | -0.119 | 0.032 | -0.004 | -0.9% |
| Skilled birth attendants | 0.197 | 0.424 | 0.084 | 19.2% |
| Antenatal care visit | 0.147 | 0.268 | 0.039 | 9.0% |
| Number of household members | -0.021 | -0.871 | 0.018 | 4.2% |
| Diarrhea | -0.058 | -0.046 | 0.003 | 0.6% |
| Low birthweight | -0.423 | -0.019 | 0.008 | 1.8% |
| Older age pregnancy | 0.082 | 0.027 | 0.002 | 0.5% |
| Maternal BMI | 0.012 | 1.506 | 0.018 | 4.0% |
| Maternal Height | 0.054 | 1.794 | 0.098 | 22.4% |
| Parity | -0.046 | -0.649 | 0.030 | 6.8% |
| Interpregnancy interval | 0.002 | 17.814 | 0.036 | 8.3% |
| Others |  |  | -0.01 | -1.2% |

##

## **Supplementary Appendix Figure 8:** Decomposing predicted changes in HAZ among children <5 (i.e. relative ranking of product coefficients for determinant domains) from 2000-2016

## **Supplementary Appendix Table 5:** Decomposition analysis for children 6-23 months from 2000-2016

| **Factors** | **Estimated**  **coefficient** | **Mean difference (2016-2000)** | **Predicted change in HAZ** | **Share of predicted change in (%)** |
| --- | --- | --- | --- | --- |
|  |  | 0.267 | 0.500 | 187.3% |
| Wealth Index (0-10) | 0.071 | 0.022 | 0.002 | 0.3% |
| Mother years of schooling | 0.037 | 2.361 | 0.087 | 17.5% |
| Father years of schooling | 0.015 | 0.770 | 0.011 | 2.2% |
| Complementary feeding | -0.068 | -0.002 | 0.000 | 0.0% |
| Altitude | 0.000 | -396.760 | 0.062 | 12.3% |
| Measles vaccine | 0.111 | 0.020 | 0.002 | 0.4% |
| Skilled birth attendants | 0.271 | 0.438 | 0.119 | 23.7% |
| Care seeking for pneumonia | -0.004 | 0.103 | 0.000 | -0.1% |
| Number of household members | -0.024 | -0.889 | 0.021 | 4.2% |
| Low birthweight | -0.400 | -0.022 | 0.009 | 1.8% |
| Maternal BMI (kg/m^2^) | 0.010 | 1.339 | 0.013 | 2.6% |
| Maternal Height (cm) | 0.058 | 1.875 | 0.109 | 21.8% |
| Parity | -0.041 | -0.586 | 0.024 | 4.8% |
| Interpregnancy interval (months) | 0.002 | 19.160 | 0.031 | 6.2% |
| Others |  |  | 0.01 | 2.2% |

## **Supplementary Appendix Figure 9:** Decomposing predicted changes in HAZ among children 6-23 months (i.e. relative ranking of product coefficients for determinant domains) from 2000-2016

## **Supplementary Appendix Table 6:** Decomposition analysis for children 24-59 months from 2000-2016

| **Factors** | **Estimated**  **coefficient** | **Mean difference (2016-2000)** | **Predicted change in HAZ** | **Share of predicted change in (%)** |
| --- | --- | --- | --- | --- |
|  |  | 0.603 | 0.480 | 79.6% |
| Wealth Index (0-10) | 0.094 | -0.415 | -0.039 | -8.1% |
| Mother years of schooling | 0.040 | 1.956 | 0.079 | 16.5% |
| Father years of schooling | 0.026 | 0.455 | 0.012 | 2.4% |
| Conditional Cash Transfer Program | 0.001 | 60.061 | 0.074 | 15.4% |
| Altitude | 0.000 | -237.110 | 0.033 | 6.9% |
| Skilled birth attendant | 0.171 | 0.412 | 0.070 | 14.7% |
| Antenatal care visit 4 and more | 0.137 | 0.284 | 0.039 | 8.1% |
| Care seeking for pneumonia | -0.005 | 0.101 | 0.000 | -0.1% |
| Urban | -0.059 | 0.135 | -0.008 | -1.7% |
| Number of household member | -0.027 | -0.836 | 0.023 | 4.8% |
| Low birthweight | -0.305 | -0.017 | 0.005 | 1.1% |
| Early pregnancy (<18 years of age) | -0.292 | 0.002 | -0.001 | -0.1% |
| Maternal BMI (kg/m^2^) | 0.012 | 1.542 | 0.019 | 3.9% |
| Maternal Height (cm) | 0.055 | 1.751 | 0.096 | 19.9% |
| Parity | -0.029 | -0.713 | 0.020 | 4.3% |
| Inter pregnancy interval (months) | 0.003 | 18.156 | 0.050 | 10.5% |
| Others |  |  | 0.007 | 1.5% |

## **Supplementary Appendix Figure 10:** Decomposing predicted changes in HAZ among children 24-59 months (i.e. relative ranking of product coefficients for determinant domains) from 2000-2016

## **Supplementary Appendix Table 7:** Decomposition analysis for children <6 months from 2000-2016

| **Factors** | **Estimated**  **coefficient** | **Mean difference (2016-2000)** | **Predicted change in HAZ** | **Share of predicted change in (%)** |
| --- | --- | --- | --- | --- |
|  |  | -0.102 | 0.090 | -88.3% |
| Wealth Index (0-10) | 0.065 | -0.292 | -0.019 | -21.2% |
| Mother years of schooling | 0.022 | 2.149 | 0.048 | 53.5% |
| Altitude | -0.0002 | 18.992 | -0.005 | -5.5% |
| Open defecation | -0.157 | -0.148 | 0.023 | 25.7% |
| Low birthweight | -1.394 | -0.017 | 0.024 | 27.1% |
| Maternal Height (cm) | 0.0392 | 1.676 | 0.066 | 72.9% |
| Others |  |  | -0.05 | -52.4% |

## **Supplementary Appendix Figure 11:** Decomposing predicted changes in HAZ among children <6 months (i.e. relative ranking of product coefficients for determinant domains) from 2000-2016

## **Supplementary Appendix Table 8:** Hierarchical linear regression models for children <6 months using individual DHS datasets, period 2000-2016

| **Domain/Indicator** | **Outcome = HAZ** | | | |
| --- | --- | --- | --- | --- |
|  | **(Height for age z-score under 6 months children)** | | | |
|  | **Period 2000 and 2016** | | | |
|  | **Time-adjusted regression coefficient** | | **Time- and confounder-adjusted  regression coefficient^1^** | |
|  |  | b estimate (95% CI) |  | b estimate (95% CI) |
|  |  | *p*-value |  | *p*-value |
|  |  |  |  |  |
| **Distal level** | | | | |
| **Basic causes & Income poverty** | | | | |
| Wealth Index |  | 0.092 (0.072; 0.112) |  | 0.065 (0.039; 0.092) |
| (0 - 10) |  | <0.001 |  | <0.001 |
|  |  |  |  |  |
| Log GDP, per capita |  | 0.341 (0.192; 0.491) |  |  |
| (USD/person) |  | <0.001 |  | -- |
|  |  |  |  |  |
| Poverty line |  | -0.012 (-0.017; -0.007) |  |  |
| (% families below) |  | <0.001 |  | -- |
|  |  |  |  |  |
| Gini coefficient for income |  | -0.024 (-0.043; -0.004) |  |  |
| 0 (perfect equality) – 1 (perfect inequality) |  | 0.019 |  | -- |
|  |  |  |  |  |
| Maternal years of schooling |  | 0.062 (0.042; 0.083) |  | 0.022 (-0.001; 0.046) |
|  |  | <0.001 |  | 0.061 |
|  |  |  |  |  |
| Paternal years of schooling |  | 0.057 (0.032; 0.081) |  |  |
|  |  | <0.001 |  | -- |
|  |  |  |  |  |
| **% of residual variance explained by covariables^2^** |  |  |  | **9.6%** |
| **Intermediate level - I** | | | | |
| **Nutrition sensitive and specific programs** | | | | |
| CCT “Juntos” program |  | -0.003 (-0.004; -0.002) |  |  |
| (No. families/1000 rural population |  | <0.001 |  | -- |
|  |  |  |  |  |
| Comprehensive Health Insurance (SIS coverage) |  | -0.009 (-0.013; -0.005) |  |  |
| (No. under-5 attendances/under-5 population) |  | <0.001 |  | -- |
|  |  |  |  |  |
| Articulated Nutrition Program (PAN log expenditure, per capita) |  | -0.411 (-0.586; -0.236) |  |  |
| (USD/person) |  | <0.001 |  | -- |
|  |  |  |  |  |
| Strategic Program of Maternal and Newborn Health (PSMN log expenditure, per capita) |  | -0.373 (-0.568; -0.177) |  |  |
| (USD/person) |  | <0.001 |  | -- |
|  |  |  |  |  |
| **% of residual variance explained by covariables^2^** |  |  |  | **9.6%** |
| **Intermediate level - II** | | | | |
| **Inadequate care and feeding practices** | | | | |
| Duration of breast feeding |  | 0.018 (-0.024; 0.061) |  |  |
| (in months) |  | 0.402 |  | -- |
|  |  |  |  |  |
| Exclusively breastfeed |  | -0.139 (-0.287; 0.01) |  |  |
| (% children) |  | 0.068 |  | -- |
|  |  |  |  |  |
| **Food insecurity** | | | | |
| Altitude  (m) |  | -0.00018 (-0.00022; -0.00013) |  | -0.0003 (-0.0003; -0.0002) |
|  |  | <0.001 |  | <0.001 |
|  |  |  |  |  |
| **Inadequate health services** | | | | |
| DPT vaccine |  | 0.08 (-0.059; 0.219) |  |  |
| (index child) |  | 0.260 |  | -- |
|  |  |  |  |  |
| Pneumococcal vaccine |  | -0.01 (-0.021; 0.001) |  |  |
| (% infants with vaccine during first 1 year) |  | 0.064 |  | -- |
|  |  |  |  |  |
| Rotavirus vaccine |  | -0.008 (-0.019; 0.003) |  |  |
| (% infants with vaccine during first 1 year) |  | 0.138 |  | -- |
|  |  |  |  |  |
| **Reproductive and maternal care** | | | | |
| Skilled attendant at birth |  | 0.461 (0.283; 0.64) |  |  |
| (index child) |  | <0.001 |  | -- |
|  |  |  |  |  |
| 4+ antenatal care visits |  | 0.343 (0.134; 0.553) |  |  |
| (index child) |  | 0.001 |  | -- |
|  |  |  |  |  |
| **Human resources for health** | | | | |
| Density of human resources for health |  | 0.028 (0.017; 0.038) |  |  |
| (Doctors, nurses and midwives per 10,000 |  | <0.001 |  | -- |
| population) |  |  |  |  |
| **Care seeking interventions for childhood illness & child micronutrient intake** | | | | |
| Oral rehydration therapy |  | -0.003 (-0.014; 0.007) |  |  |
| (% children with diarrhea receiving ORT) |  | 0.543 |  | -- |
|  |  |  |  |  |
| Care seeking for pneumonia |  | 0.018 (0.012; 0.024) |  |  |
| (% children with pneumonia seeking care) |  | <0.001 |  | -- |
|  |  |  |  |  |
| **Unhealthy household environment** | | | | |
| Urban locality |  | 0.533 (0.391; 0.676) |  |  |
| (index household) |  | <0.001 |  | -- |
|  |  |  |  |  |
| Open defecation |  | -0.529 (-0.708; -0.351) |  | -0.157 (-0.338; 0.024) |
| (index household ) |  | <0.001 |  | 0.089 |
|  |  |  |  |  |
| Access to Improved drinking water sources |  | 0.117 (-0.078; 0.312) |  |  |
| (index household) |  | 0.239 |  | -- |
|  |  |  |  |  |
| Number of household members  (index household) |  | -0.011 (-0.042; 0.02) |  |  |
|  |  | 0.497 |  | -- |
|  |  |  |  |  |
| **% of residual variance explained by covariables^2^** |  |  |  | **12.2%** |
| **Proximal level** | | | | |
| **Infections** | | | | |
| ARI infection |  | -0.131 (-0.281; 0.019) |  |  |
| (index child within last 2 weeks) |  | 0.086 |  | -- |
|  |  |  |  |  |
| Diarrhea infection |  | -0.2 (-0.399; -0.002) |  |  |
| (index child within last 2 weeks) |  | 0.048 |  | -- |
|  |  |  |  |  |
| **Child characteristics** | | | | |
| Low birthweight |  | -1.554 (-1.84; -1.267) |  | -1.394 (-1.744; -1.045) |
| (index child) |  | <0.001 |  | <0.001 |
|  |  |  |  |  |
| **Maternal characteristics** |  |  |  |  |
| Maternal age |  | 0.001 (-0.009; 0.012) |  |  |
| (years) |  | 0.775 |  | -- |
|  |  |  |  |  |
| Adolescent birth (<18 years of age) |  | 0.002 (-0.243; 0.247) |  |  |
| (current birth for index mother) |  | 0.985 |  | -- |
|  |  |  |  |  |
| Older mother birth (≥ 35 years)  (current birth for index mother) |  | -0.042 (-0.258; 0.174) |  |  |
|  |  | 0.705 |  | -- |
|  |  |  |  |  |
| Anemia during pregnancy |  | 0.007 (-0.135; 0.148) |  |  |
| (current pregnancy for index mother) |  | 0.928 |  | -- |
|  |  |  |  |  |
| Body mass index (kg/m^2^) |  | 0.017 (0; 0.033) |  |  |
| (index mother) |  | 0.044 |  | -- |
|  |  |  |  |  |
| Height (cm) |  | 0.056 (0.044; 0.068) |  | 0.039 (0.027; 0.051) |
| (index mother) |  | <0.001 |  | <0.001 |
|  |  |  |  |  |
| Parity (No. children) |  | -0.044 (-0.082; -0.007) |  |  |
| (index mother) |  | 0.020 |  | -- |
|  |  |  |  |  |
| Interpregnancy interval (months) |  | 0.002 (0; 0.004) |  |  |
| (index mother) |  | 0.117 |  | -- |
|  |  |  |  |  |
| **% of residual variance explained by covariables^2^** |  |  |  | **20.5%** |
| **Time** |  |  |  |  |
| **Time** |  |  |  |  |
| Year |  | -0.006 (-0.015; 0.002) |  | -0.019 (-0.027; -0.01) |
|  |  | 0.143 |  | <0.001 |
|  |  |  |  |  |

## **Supplementary Appendix Table 9:** Hierarchical linear regression models for children 6 – 23 months using individual DHS datasets, period 2000-2016

| **Domain/Indicator** | **Outcome = HAZ** | | | |
| --- | --- | --- | --- | --- |
|  | **(Height for age z-score among 6 - 23 months children)** | | | |
|  | **Period 2000 and 2016** | | | |
|  | **Time-adjusted regression coefficient** | | **Time- and confounder-adjusted  regression coefficient^1^** | |
|  |  | b estimate (95% CI) |  | b estimate (95% CI) |
|  |  | *p*-value |  | *p*-value |
|  |  |  |  |  |
| **Distal level** | | | | |
| **Basic causes & Income poverty** | | | | |
| Wealth Index |  | 0.137 (0.127;0.147) |  | 0.071 (0.056; 0.086) |
| (0 - 10) |  | <0.001 |  | <0.001 |
|  |  |  |  |  |
| Poverty line |  | -0.022 (-0.024;-0.019) |  |  |
| (% families below) |  | <0.001 |  | -- |
|  |  |  |  |  |
| Gini coefficient (for income) |  | -0.026 (-0.037;-0.015) |  |  |
| 0 (perfect equality) – 1 (perfect inequality) |  | <0.001 |  | -- |
|  |  |  |  |  |
| Maternal years of schooling |  | 0.095 (0.086;0.104) |  | 0.037 (0.026; 0.048) |
|  |  | <0.001 |  | <0.001 |
|  |  |  |  |  |
| Paternal years of schooling |  | 0.088 (0.077;0.099) |  | 0.015 (0.002; 0.027) |
|  |  | <0.001 |  | 0.019 |
|  |  |  |  |  |
| **% of residual variance explained by covariables^2^** |  |  |  | **19.8%** |
| **Intermediate level - I** | | | | |
| **Nutrition sensitive and specific programs** | | | | |
| CCT “Juntos” program |  | -0.004 (-0.004;-0.003) |  |  |
| (No. families/1000 rural population |  | <0.001 |  | -- |
|  |  |  |  |  |
| Comprehensive Health Insurance (SIS coverage) |  | -0.01 (-0.012;-0.007) |  |  |
| (No. under-5 attendances/under-5 population) |  | <0.001 |  | -- |
|  |  |  |  |  |
| Articulated Nutrition Program (PANlog) expenditure |  | -0.573 (-0.67;-0.476) |  |  |
| (USD/person) |  | <0.001 |  | -- |
|  |  |  |  |  |
| Strategic Program of Maternal and Newborn Health (PSMN log expenditure), per capita |  | -0.412 (-0.511;-0.313) |  |  |
| (USD/person) |  | <0.001 |  | -- |
|  |  |  |  |  |
| **% of residual variance explained by covariables^2^** |  |  |  | **19.8%** |
| **Intermediate level - II** | | | | |
| **Inadequate care and feeding practices** | | | | |
| Duration of breast feeding |  | -0.042 (-0.049;-0.035) |  |  |
| (in months) |  | <0.001 |  | -- |
|  |  |  |  |  |
| Complementary feeding |  | -0.076 (-0.16;0.007) |  | -0.068 (-0.147; 0.011) |
| (index child) |  | 0.074 |  | 0.092 |
|  |  |  |  |  |
| **Food insecurity** | | | | |
| Altitude  (m) |  | -0.00022 (-0.00025;-0.0002) |  | -0.00016 (-0.0002; -0.00011) |
|  |  | <0.001 |  | <0.001 |
|  |  |  |  |  |
| **Inadequate health services** | | | | |
| DPT vaccine |  | 0.26 (0.09;0.43) |  |  |
| (% infants with 3 doses)  (index child) |  | 0.003 |  | -- |
|  |  |  |  |  |
| Measles vaccine |  | -0.16 (-0.232;-0.088) |  | 0.111 (0.017; 0.204) |
| (% infants)  (index child) |  | <0.001 |  | 0.02 |
|  |  |  |  |  |
| Pneumococcal vaccine |  | -0.01 (-0.017;-0.003) |  |  |
| (% infants with vaccine during first 1 year) |  | 0.003 |  | -- |
|  |  |  |  |  |
| Rotavirus vaccine |  | -0.0002 (-0.0063;0.0058) |  |  |
| (% infants with vaccine during first 1 year) |  | 0.939 |  | -- |
|  |  |  |  |  |
| **Reproductive and maternal care** | | | | |
| Skilled attendant at birth |  | 0.838 (0.735;0.94) |  | 0.271 (0.171; 0.371) |
| (index child) |  | <0.001 |  | <0.001 |
|  |  |  |  |  |
| 4+ antenatal care visits |  | 0.558 (0.439;0.677) |  |  |
| (index child) |  | <0.001 |  | -- |
|  |  |  |  |  |
| **Human resources for health** | | | | |
| Density of human resources for health |  | 0.043 (0.037;0.049) |  |  |
| (Doctors, nurses and midwives per 10,000 |  | <0.001 |  | -- |
| population) |  |  |  |  |
| **Care seeking interventions for childhood illness & child micronutrient intake** | | | | |
| Oral rehydration therapy |  | 0.0002 (-0.0048;0.0052) |  |  |
| (% children with diarrhea receiving ORT) |  | 0.939 |  | -- |
|  |  |  |  |  |
| Care seeking for pneumonia |  | 0.023 (0.02;0.026) |  | -0.00413 (-0.0084; 0.00015) |
| (% children with pneumonia seeking care) |  | <0.001 |  | 0.058 |
|  |  |  |  |  |
| Vitamin A supplementation |  | -0.354 (-0.471;-0.238) |  |  |
| (index child) |  | <0.001 |  | -- |
|  |  |  |  |  |
| **Unhealthy household environment** | | | | |
| Urban locality |  | 0.793 (0.719;0.868) |  |  |
| (index household) |  | <0.001 |  | -- |
|  |  |  |  |  |
| Open defecation |  | -0.655 (-0.755;-0.555) |  |  |
| (index household) |  | <0.001 |  | -- |
|  |  |  |  |  |
| Access to improved drinking water sources |  | 0.289 (0.18;0.398) |  |  |
| (index household) |  | <0.001 |  | -- |
|  |  |  |  |  |
| Number of household members  (index household) |  | -0.045 (-0.063;-0.028) |  | -0.024 (-0.041; -0.006) |
|  |  | <0.001 |  | 0.008 |
|  |  |  |  |  |
| **% of residual variance explained by covariables^2^** |  |  |  | **21.7%** |
| **Proximal level** |  |  |  |  |
| **Infections** |  |  |  |  |
| ARI infection |  | -0.055 (-0.127;0.016) |  |  |
| (index child within last 2 weeks) |  | 0.13 |  | -- |
|  |  |  |  |  |
| Diarrhea infection |  | -0.178 (-0.258;-0.099) |  |  |
| (index child within last 2 weeks) |  | <0.001 |  | -- |
|  |  |  |  |  |
| **Inadequate dietary intake** |  |  |  |  |
| Infant and young child minimum dietary diversity |  | 0.176 (0.095; 0.257) |  |  |
| (% children consuming at least 4 food groups) |  | <0.001 |  | -- |
|  |  |  |  |  |
| Infant and young child minimum dietary diversity (continuous) |  | 0.037 (0.012; 0.062) |  |  |
|  |  | 0.004 |  | -- |
|  |  |  |  |  |
| Grains, roots, and tubers |  | -0.102 (-0.245; 0.042) |  |  |
| (index child) |  | 0.164 |  | -- |
|  |  |  |  |  |
| Legumes and nuts |  | -0.225 (-0.295;-0.155) |  |  |
| (index child) |  | <0.001 |  | -- |
|  |  |  |  |  |
| Dairy products |  | 0.273 (0.198;0.348) |  |  |
| (index child) |  | <0.001 |  | -- |
|  |  |  |  |  |
| Flesh foods and Eggs |  | 0.07 (-0.032;0.172) |  |  |
| (index child) |  | 0.18 |  | -- |
|  |  |  |  |  |
| Vitamin-A rich fruits and vegetables |  | 0.059 (-0.053;0.171) |  |  |
| (index child) |  | 0.304 |  | -- |
|  |  |  |  |  |
| Other fruits and vegetables |  | 0.103 (0.023;0.183) |  |  |
| (index child) |  | 0.011 |  | -- |
|  |  |  |  |  |
| **Child characteristics** | | | | |
| Low birthweight |  | -0.744 (-0.89;-0.598) |  | -0.4 (-0.548; -0.253) |
| (index child) |  | <0.001 |  | <0.001 |
|  |  |  |  |  |
| **Maternal characteristics** |  |  |  |  |
| Maternal age |  | 0.001 (-0.004;0.006) |  |  |
| (years) |  | 0.605 |  | -- |
|  |  |  |  |  |
| Adolescent birth (<18 years of age) |  | -0.304 (-0.41;-0.198) |  |  |
| (current birth for index mother) |  | <0.001 |  | -- |
|  |  |  |  |  |
| Older mother birth (≥35 years) |  | -0.044 (-0.139;0.051) |  |  |
| (current birth for index mother) |  | 0.364 |  | -- |
|  |  |  |  |  |
| Anemia during pregnancy |  | -0.039 (-0.135;0.058) |  |  |
| (current birth for index mother) |  | 0.433 |  | -- |
|  |  |  |  |  |
| Body mass index (kg/m^2^) |  | 0.021 (0.013;0.029) |  | 0.01 (0.001; 0.019) |
| (index mother) |  | <0.001 |  | 0.033 |
|  |  |  |  |  |
| Height (cm) |  | 0.074 (0.068;0.08) |  | 0.058 (0.051; 0.065) |
| (index mother) |  | <0.001 |  | <0.001 |
|  |  |  |  |  |
| Parity (No. children) |  | -0.149 (-0.168;-0.129) |  | -0.041 (-0.071; -0.011) |
| (index mother) |  | <0.001 |  | 0.008 |
|  |  |  |  |  |
| Interpregnancy interval (months) |  | 0.005 (0.004;0.006) |  | 0.002 (0.001; 0.003) |
| (index mother) |  | <0.001 |  | 0.001 |
|  |  |  |  |  |
| **% of residual variance explained by covariables^2^** |  |  |  | **26.9%** |
| **Time** |  |  |  |  |
| **Time** |  |  |  |  |
| Year |  | 0.017 (0.012;0.021) |  | -0.011 (-0.018; -0.004) |
|  |  | <0.001 |  | 0.002 |
|  |  |  |  |  |

## **Supplementary Appendix Table 10:** Hierarchical linear regression models for children 24 – 59 months using individual DHS datasets, period 2000-2016

| **Domain/Indicator** | **Outcome = HAZ** | | | |
| --- | --- | --- | --- | --- |
|  | **(Height for age z-score among 24 months and above children)** | | | |
|  | **Period 2000 and 2016** | | | |
|  | **Time-adjusted regression coefficient** | | **Time- and confounder-adjusted  regression coefficient^1^** | |
|  |  | b estimate (95% CI) |  | b estimate (95% CI) |
|  |  | *p*-value |  | *p*-value |
|  |  |  |  |  |
| **Distal level** | | | | |
| **Basic causes & Income poverty** | | | | |
| Wealth Index |  | 0.167 (0.16; 0.175) |  | 0.094 (0.084; 0.104) |
| (0 - 10) |  | <0.001 |  | <0.001 |
|  |  |  |  |  |
| Log GDP, per capita |  | 0.72 (0.666; 0.775) |  |  |
| (USD/person) |  | <0.001 |  | -- |
|  |  |  |  |  |
| Poverty line |  | -0.024 (-0.026; -0.022) |  |  |
| (% families below) |  | <0.001 |  | -- |
|  |  |  |  |  |
| Gini coefficient (for income) |  | -0.036 (-0.044; -0.028) |  |  |
| 0 (perfect equality) – 1 (perfect inequality) |  | <0.001 |  | -- |
|  |  |  |  |  |
| Maternal years of schooling |  | 0.112 (0.106; 0.118) |  | 0.04 (0.033; 0.048) |
|  |  | <0.001 |  | <0.001 |
|  |  |  |  |  |
| Paternal years of schooling |  | 0.11 (0.102; 0.117) |  | 0.026 (0.018; 0.033) |
|  |  | <0.001 |  | <0.001 |
|  |  |  |  |  |
| **% of residual variance explained by covariables^2^** |  |  |  | **31.4%** |
| **Intermediate level - I** | | | | |
| **Nutrition sensitive and specific programs** | | | | |
| CCT “Juntos” program |  | -0.004 (-0.004; -0.003) |  | 0.001 (0; 0.002) |
| (No. families/1000 rural population) |  | <0.001 |  | 0.003 |
|  |  |  |  |  |
| Comprehensive Health Insurance (SIS coverage) |  | -0.01 (-0.012; -0.008) |  |  |
| (No. under-5 attendances/under-5 population) |  | <0.001 |  | -- |
|  |  |  |  |  |
| Articulated Nutrition Program (PANlog) expenditure |  | -0.678 (-0.758; -0.598) |  |  |
| (USD/person) |  | <0.001 |  | -- |
|  |  |  |  |  |
| Strategic Program of Maternal and Newborn Health (PSMN log expenditure), per capita |  | -0.547 (-0.633; -0.461) |  |  |
| (USD/person) |  | <0.001 |  | -- |
|  |  |  |  |  |
| **% of residual variance explained by covariables^2^** |  |  |  | **31.5%** |
| **Intermediate level - II** | | | | |
| **Inadequate care and feeding practices** | | | | |
| Duration of breastfeeding |  | -0.014 (-0.017; -0.011) |  |  |
| (in months) |  | <0.001 |  | -- |
|  |  |  |  |  |
| **Food insecurity** | | | | |
| Altitude  (m) |  | -0.00025 (-0.00027; -0.00023) |  | -0.00014 (-0.00017; -0.00011) |
|  |  | <0.001 |  | <0.001 |
|  |  |  |  |  |
| **Inadequate health services** | | | | |
| DPT vaccine |  | 0.188 (0.061; 0.315) |  |  |
| (% infants with 3 doses)  (index child) |  | 0.004 |  | -- |
|  |  |  |  |  |
| Measles vaccine |  | 0.253 (0.157; 0.348) |  |  |
| (% infants with 3 doses) |  | <0.001 |  | -- |
| (index child) |  |  |  |  |
| Pneumococcal vaccine |  | -0.021 (-0.027; -0.015) |  |  |
| (% infants with vaccine during first 1 year) |  | <0.001 |  | -- |
|  |  |  |  |  |
| Rotavirus vaccine |  | -0.011 (-0.017; -0.006) |  |  |
| (% infants with vaccine during first 1 year) |  | <0.001 |  | -- |
|  |  |  |  |  |
| **Reproductive and maternal care** | | | | |
| Skilled attendant at birth |  | 0.873 (0.8; 0.946) |  | 0.171 (0.098; 0.244) |
| (index child) |  | <0.001 |  | <0.001 |
|  |  |  |  |  |
| 4+ antenatal care visits |  | 0.74 (0.65; 0.829) |  | 0.137 (0.054; 0.22) |
| (index child) |  | <0.001 |  | 0.001 |
|  |  |  |  |  |
| **Human resources for health** | | | | |
| Density of human resources for health |  | 0.043 (0.038; 0.047) |  |  |
| (Doctors, nurses and midwives per 10,000 |  | <0.001 |  | -- |
| population) |  |  |  |  |
| **Care seeking interventions for childhood illness & child micronutrient intake** | | | | |
| Oral rehydration therapy |  | -0.001 (-0.006; 0.003) |  |  |
| (% children with diarrhea receiving ORT) |  | 0.494 |  | -- |
|  |  |  |  |  |
| Care seeking for pneumonia |  | 0.024 (0.022; 0.026) |  | -0.005 (-0.008; -0.001) |
| (% children with pneumonia seeking care) |  | <0.001 |  | -- |
|  |  |  |  |  |
| Vitamin A Supplementation |  | -0.416 (-0.522; -0.311) |  |  |
| (index child) |  | <0.001 |  | -- |
|  |  |  |  |  |
| **Unhealthy household environment** | | | | |
| Urban locality |  | 0.875 (0.817; 0.933) |  | -0.059 (-0.127; 0.008) |
| (index household) |  | <0.001 |  | 0.086 |
|  |  |  |  |  |
| Open defecation |  | -0.795 (-0.875; -0.716) |  |  |
| (index household) |  | <0.001 |  | -- |
|  |  |  |  |  |
| Access to improved drinking water sources |  | 0.354 (0.271; 0.438) |  |  |
| (index household) |  | <0.001 |  | -- |
|  |  |  |  |  |
| Number of household members  (index household) |  | -0.059 (-0.073; -0.046) |  | -0.027 (-0.039; -0.015) |
|  |  | <0.001 |  | <0.001 |
|  |  |  |  |  |
| **% of residual variance explained by covariables^2^** |  |  |  | **32.9%** |
| **Proximal level** | | | | |
| **Infections** | | | | |
| ARI infection |  | 0.011 (-0.042; 0.063) |  |  |
| (index child within last 2 weeks) |  | 0.695 |  | -- |
|  |  |  |  |  |
| Diarrhea infection |  | -0.206 (-0.279; -0.133) |  |  |
| (index child within last 2 weeks) |  | <0.001 |  | -- |
|  |  |  |  |  |
| **Child characteristics** | | | | |
| Low birthweight |  | -0.509 (-0.606; -0.412) |  | -0.305 (-0.398; -0.212) |
| (index child) |  | <0.001 |  | <0.001 |
|  |  |  |  |  |
| **Maternal characteristics** |  |  |  |  |
| Maternal age |  | 0.004 (0.001; 0.008) |  |  |
| (years) |  | 0.015 |  | -- |
|  |  |  |  |  |
| Adolescent birth (<18 years of age) |  | -0.319 (-0.415; -0.224) |  | -0.292 (-0.626; 0.041) |
| (current birth for index mother) |  | <0.001 |  | 0.086 |
|  |  |  |  |  |
| Older mother birth (≥35 years) |  | -0.044 (-0.115; 0.027) |  |  |
| (current birth for index mother) |  | 0.225 |  | -- |
|  |  |  |  |  |
| Anemia during pregnancy |  | -0.042 (-0.11; 0.026) |  |  |
| (current birth for index mother) |  | 0.223 |  | -- |
|  |  |  |  |  |
| Body mass index (kg/m^2^) |  | 0.033 (0.027; 0.038) |  | 0.012 (0.006; 0.018) |
| (index mother) |  | <0.001 |  | <0.001 |
|  |  |  |  |  |
| Height (cm) |  | 0.081 (0.076; 0.086) |  | 0.055 (0.048; 0.061) |
| (index mother) |  | <0.001 |  | <0.001 |
|  |  |  |  |  |
| Parity (No. children) |  | -0.161 (-0.174; -0.149) |  | -0.029 (-0.05; -0.008) |
| (index mother) |  | <0.001 |  | 0.007 |
|  |  |  |  |  |
| Interpregnancy interval (months) |  | 0.006 (0.005; 0.006) |  | 0.003 (0.002; 0.003) |
| (index mother) |  | <0.001 |  | <0.001 |
|  |  |  |  |  |
| **% of residual variance explained by covariables^2^** |  |  |  | **35.3%** |
| **Time** |  |  |  |  |
| **Time** |  |  |  |  |
| Year |  | 0.038 (0.034; 0.041) |  | 0.015 (0.009; 0.021) |
|  |  | <0.001 |  | <0.001 |
|  |  |  |  |  |

## **Supplementary Appendix Table 11:** Multilevel linear models for under-5 stunting prevalence (2 years’ time-lag), period 2000-2016

**factors statistically significant at p<0.05 are highlighted in red*

| **Domain/Indicator** | **Outcome = Stunting** | | |
| --- | --- | --- | --- |
|  | **(Percentage of stunting among under-5 children^1^)** | | |
|  | **Period 2000-2016^a^** | | |
|  | **Time-adjusted regression coefficient** | |  |
|  |  |  |  |
|  |  | b estimate (95% CI) | b estimate (95% CI) |
|  |  | *p*-value | *p*-value |
|  |  |  | Effect size^2^ |
| **Level 4 Model - Distal: National level & Macro variables^b^** | | | |
| **Basic causes & Income poverty** | | | |
| Log Gross domestic product (GDP) per capita |  | -1.379 (-5.893;2.136) |  |
| (USD/person) |  | 0.55 | - - |
|  |  |  |  |
| Poverty line |  | 0.195 (0.106;0.285) | 0.182 (0.111;0.254) |
| (% families below) |  | <0.0001 | <0.0001 |
|  |  |  | 0.08 |
| Gini coefficient for income  0 (perfect equality) – 1 (perfect inequality) |  | 0.038 (-0.201;0.276) |  |
|  |  | 0.758 | - - |
|  |  |  |  |
| Median years of schooling among |  | -2.218 (-3.266;-1.170) | -1.937 (-2.816;-1.057) |
| women 15 years or older |  | <0.0001 | <0.0001 |
|  |  |  | 0.03 |
| **% of residual variance explained by covariables^3^** |  |  | **64%** |
| **Level 3 Model - Intermediate I: Nutrition sensitive and specific programs^c^** | | | |
| **Nutrition specific and sensitive programs** | | | |
| Conditional cash transfer “Juntos” program |  | -0.052 (-0.079;-0.026) | -0.046 (-0.072;-0.021) |
| (No. families/1000 rural population |  | <0.0001 | <0.0001 |
|  |  |  | 1.64 |
| SIS^4^ coverage |  | 0.021 (0.010;0.032) | -0.012 (-0.025;-0.001) |
| (No. under-5 attendances/under-5 population) |  | <0.0001 | 0.08 |
|  |  |  | 1.64 |
| Articulated Nutrition Program (PAN^5^ log expenditure, per capita) |  | -0.768 (-1.161;-0.375) | -0.348 (-0.700;0.005) |
| (USD/person) |  | <0.0001 | 0.05 |
|  |  |  | 1.64 |
| Strategic Program of Maternal and Newborn Health (PSMN^6^ log expenditure, per capita |  | -0.595 (-0.951;-0.239) |  |
| (USD/person) |  | 0.001 | - - |
|  |  |  |  |
| **% of residual variance explained by covariables^3^** |  |  | **68%** |
| **Level 2 Model - Intermediate II: Community and Household-level variables^d^** | | | |
| **Inadequate health services** | | | |
| DPT vaccine |  | -0.048 (-0.133;0.038) |  |
| (% infants with 3 doses) |  | 0.277 | - - |
|  |  |  |  |
| Measles vaccine |  | -0.085 (-0.169;-0.001) |  |
| (% infants) |  | 0.047 | - - |
|  |  |  |  |
| Pneumococcal vaccine |  | -0.064 (-0.098;-0.029) | -0.051 (-0.096;-0.006) |
| (% infants with vaccine during first 1 year) |  | <0.0001 | 0.072 |
|  |  |  | 0.02 |
| Rotavirus vaccine |  | -0.027 (-0.096;-0.029) |  |
| (% infants with vaccine during first 1 year) |  | <0.0001 | - - |
|  |  |  |  |
| **Composite Coverage** | | | |
| Composite coverage index^6^ |  | -0.185 (-0.388;0.018) | 0.178 (-0.009;0.364) |
|  |  | 0.075 | 0.058 |
|  |  |  | 0.02 |
| **Reproductive and maternal care** | | | |
| Skilled attendant for last birth |  | -0.223 (-0.319;-0.127) | -0.15 (-0.242;-0.057) |
| (% mothers) |  | <0.0001 | 0.004 |
|  |  |  | 0.07 |
| 4+ antenatal care visits for last birth |  | 0.016 (-0.132;0.163) |  |
| (% mothers) |  | 0.835 | - - |
|  |  |  |  |
| **Human resources for health** | | | |
| Density of human resources for health |  | -0.197 (-0.376;-0.018) |  |
| (Doctors, nurses and midwives per 10,000 |  | 0.031 | - - |
| population) |  |  |  |
| **Care seeking interventions for childhood illness & child micronutrient intake** | | | |
| Care seeking for pneumonia |  | 0.014 (-0.027;0.054) |  |
| (% children with pneumonia seeking care) |  | 0.507 | - - |
|  |  |  |  |
| Oral rehydration therapy |  | 4.77e-6 (-0.056;0.056) |  |
| (% children with diarrhea receiving ORT) |  | 1 | - - |
|  |  |  |  |
| ARI receiving antibiotic treatment |  | 0.183 (-0.179;0.545) |  |
| (% children with ARI receiving antibiotics) |  | 0.322 | - - |
|  |  |  |  |
| Vitamin A supplementation |  | -0.145 (-0.264;-0.027) |  |
| (% children) |  | 0.016 | - - |
|  |  |  |  |
| **Unhealthy household environment** | | | |
| Urbanization |  | -0.492 (-0.586;-0.398) | -0.284 (-0.397;-0.172) |
| (% of urban population) |  | <0.0001 | <0.0001 |
|  |  |  | 0.04 |
| Open defecation |  | 0.451 (0.256;0.646) | 0.283 (0.159;0.407) |
| (% of population ) |  | <0.0001 | 0.002 |
|  |  |  | 0.03 |
| Improved drinking water source |  | -0.141 (-0.241;-0.041) |  |
| (% of population) |  | 0.006 | - - |
|  |  |  |  |
| **% of residual variance explained by covariables^3^** |  |  | **75%** |
| **Level 1 Model - Proximal: Individual-level variables^e^** |  |  |  |
| **Infections** | | | |
| ARI infection |  | 0.244 (0.027;0.460) | 0.165 (0.053;0.277) |
| (% children within last 2 weeks) |  | 0.028 | 0.004 |
|  |  |  | 0.03 |
| Diarrhoea infection |  | 0.284 (0.107;0.462) |  |
| (% children within last 2 weeks) |  | 0.002 | - - |
|  |  |  |  |
| **Child characteristics** | | | |
| Low birthweight |  | -0.214 (-0.526;0.099) |  |
| (% children) |  | 0.181 | - - |
|  |  |  |  |
| **Maternal characteristics** | | | |
| Anemia |  | 0.133 (0.017;0.249) |  |
| (% women 15-49 years) |  | 0.024 | - - |
|  |  |  |  |
| Young mother births (<18 years) |  | 0.072 (-0.686;0.829) |  |
| (% births within last 5 years) |  | 0.853 | - - |
|  |  |  |  |
| BMI |  | -1.837 (-3.513;-0.160) |  |
| (mean, mothers 15-49 years) |  | 0.032 | - - |
|  |  |  |  |
| Height |  | -1.491 (-3.354;0.372) |  |
| (mean, mothers 15-49 years) |  | 0.117 | - - |
|  |  |  |  |
| Interpregnancy interval |  | -0.089 (-0.354;0.177) |  |
| (median, mothers 15-49 years) |  | 0.512 | - - |
|  |  |  |  |
| Age |  | -1.758 (-2.726;-0.790) | -0.965 (-1.853;-0.077) |
| (mean, mothers 15-49) |  | <0.0001 | 0.033 |
|  |  |  | 0.02 |
| Total fertility rate |  | 4.539 (2.732;6.347) |  |
| (mean, mothers 15-49 years) |  | <0.0001 | - - |
|  |  |  |  |
| **% of residual variance explained by covariables^3^** |  |  | **75%** |
| **Time** | | | |
| **Time** | | | |
| Year |  | -1.283 (-1.549;-1.016) | 0.187 (-0.177;0.552) |
|  |  | <0.0001 | 0.313 |
|  |  |  | 0.15 |

*factors statistically significant at p<0.05 are highlighted in red

| *Variables significant at p<0.20 in bivariate analysis were entered into linear multilevel mixed-effects models. | |
| --- | --- |
| ^1^ Variables in each group are adjusted for all other variables in the same group or above. |  |
| ^2^ Calculated using Cohen’s F^2^ to estimate effect size as ([R^2^ of the full model – R^2^ of the restricted model]/[1 - R^2^ of the full model]) with the restricted model excluding the variable to be evaluated (without the effect of interest) and with the random intercept variance constrained to be the same as the full model, and defined as: small effect with F^2^ ≥ 0.02, medium effect with F^2^ ≥ 0.15, and large effect with F^2^ ≥ 0.35 (Cohen J.E., 1988). | |
| ^3^ Calculated as ([residual variance component of null model - residual variance covariable model]/[residual variance null model]), with the null model being (outcome and department). | |
| ^4^Comprehensive Health Insurance “Seguro Integral de Salud” (SIS, acronym in Spanish). | |
| ^5^Articulated Nutrition Program (PAN, acronym in Spanish) | |
| ^6^Strategic Program of Maternal and Neonatal Health (PSMN, acronym in Spanish) | |
| ^7^Composite coverage index: Summary measure of intervention coverage along the reproductive, maternal, newborn, and child health continuum. It is a weighted mean of the coverage for interventions from 4 domains: i) reproductive services (family planning coverage), ii) maternal and newborn care (antenatal care and skilled birth attendant), iii) immunization (BCG, three doses of DPT3, and measles vaccines), and iv) management of illness (ORT and care seeking for pneumonia). | |
| ^a^ Given that we considered a two-year time lag between predictors and outcome, our units of analysis are: 360 for the full time period (24 departments x 15 years, 2000 to 2016), 192 for the pre-PAN time period (24 departments x 8 years, 2000 to 2007), and 168 for the post-PAN time period (24 departments x 7 years, 2008 to 2016). | |
| ^b^ Level 4 multivariable model includes all statistically significant (p<0.1) distal variables as listed. | |
| ^c^ Level 3 multivariable model includes level 4 model + all statistically significant (p<0.1) intermediate I variables as listed. | |
| ^d^ Level 2 multivariable model includes level 4 model + 3 model + all statistically significant (p<0.1) intermediate II variables as listed. | |
| ^e^ Level 1 multivariable model includes level 4 model + 3 model + 2 model + all statistically significant (p<0.1) proximal variables as listed. | |

## **Supplementary Appendix Table 12:** Multilevel linear models for under-5 stunting prevalence (2 years’ time-lag), period 2000-2007

**factors statistically significant at p<0.05 are highlighted in red*

| **Domain/Indicator** | **Outcome = Stunting** | | | |
| --- | --- | --- | --- | --- |
|  | **(Percentage of stunting among under-5 children^1^)** | | | |
|  | **Period 2000-2007^a^** | | | |
|  | **Time-adjusted regression coefficient** | | **Time- and confounder-adjusted^1^** | |
|  |  |  | **regression coefficient** | |
|  |  | b estimate (95% CI) |  | b estimate (95% CI) |
|  |  | *p*-value |  | *p*-value |
|  |  |  |  | Effect size^2^ |
| **Level 4 Model - Distal: National level & Macro variables^b^** | | | | |
| **Basic causes & Income poverty** | | | | |
| Log Gross domestic product (GDP) per capita |  | -2.861 (-5.336;-0.385) |  | -3.173 (-6.210;-0.136) |
| (USD/person) |  | 0.024 |  | 0.04 |
|  |  |  |  | 0.02 |
| Poverty line |  | 0.129 (0.031;0.226) |  | 0.158 (0.056;0.260) |
| (% families below) |  | 0.01 |  | 0.002 |
|  |  |  |  | 0.05 |
| Gini coefficient for income  0 (perfect equality) – 1 (perfect inequality) |  | -0.046 (-0.414;0.322) |  |  |
|  |  | 0.807 |  | - - |
|  |  |  |  |  |
| Median years of schooling among |  | -2.201 (-3.233;-1.169) |  | -2.082 (-3.141;-1.022) |
| women 15 years or older |  | <0.0001 |  | <0.0001 |
|  |  |  |  | -0.01 |
| **% of residual variance explained by covariables^3^** |  |  |  | **10%** |
| **Level 3 Model - Intermediate I: Nutrition sensitive and specific programs^c^** | | | | |
| **Nutrition specific and sensitive programs** | | | | |
| Conditional cash transfer “Juntos” program |  | -0.002 (-0.037;0.033) |  |  |
| (No. families/1000 rural population |  | 0.927 |  | - - |
|  |  |  |  |  |
| SIS^4^ coverage |  | 0.006 (-0.002;0.013) |  |  |
| (No. under-5 attendances/under-5 population) |  | 0.138 |  | - - |
|  |  |  |  |  |
| Articulated Nutrition Program (PAN^5^ log expenditure, per capita) |  |  |  |  |
| (USD/person) |  | - - |  | - - |
|  |  |  |  |  |
| Strategic Program of Maternal and Newborn Health (PSMN^6^ log expenditure, per capita |  |  |  |  |
| (USD/person) |  | - - |  | - - |
|  |  |  |  |  |
| **% of residual variance explained by covariables^3^** |  |  |  | **- -** |
| **Level 2 Model - Intermediate II: Community and Household-level variables^d^** | | | | |
| **Inadequate health services** | | | | |
| DPT vaccine |  | 0.041 (-0.082;0.164) |  |  |
| (% infants with 3 doses) |  | 0.515 |  | - - |
|  |  |  |  |  |
| Measles vaccine |  | 0.005 (-0.136;0.147) |  |  |
| (% infants) |  | 0.944 |  | - - |
|  |  |  |  |  |
| Pneumococcal vaccine |  |  |  |  |
| (% infants with vaccine during first 1 year) |  | - - |  | - - |
|  |  |  |  |  |
| Rotavirus vaccine |  |  |  |  |
| (% infants with vaccine during first 1 year) |  | - - |  | - - |
|  |  |  |  |  |
| **Composite Coverage** | | | | |
| Composite coverage index^6^ |  | 0.163 (-0.035;0.361) |  | 0.197 (0.025;0.369) |
|  |  | 0.107 |  | 0.024 |
|  |  |  |  | 0.04 |
| **Reproductive and maternal care** | | | | |
| Skilled attendant for last birth |  | -0.053 (-0.176;0.070) |  |  |
| (% mothers) |  | 0.401 |  | - - |
|  |  |  |  |  |
| 4+ antenatal care visits for last birth |  | 0.049 (-0.126;0.225) |  |  |
| (% mothers) |  | 0.581 |  | - - |
|  |  |  |  |  |
| **Human resources for health** | | | | |
| Density of human resources for health |  | -0.564 (-1.024;-0.104) |  | -0.47 (-0.921;-0.018) |
| (Doctors, nurses and midwives per 10,000 |  | 0.016 |  | 0.042 |
| population) |  |  |  | 0.03 |
| **Care seeking interventions for childhood illness & child micronutrient intake** | | | | |
| Care seeking for pneumonia |  | 0.047 (-0.009;0.103) |  |  |
| (% children with pneumonia seeking care) |  | 0.1 |  | - - |
|  |  |  |  |  |
| Oral rehydration therapy |  | 0.009 (-0.053;0.071) |  |  |
| (% children with diarrhea receiving ORT) |  | 0.776 |  | - - |
|  |  |  |  |  |
| ARI receiving antibiotic treatment |  | 0.020 (-0.702;0.742) |  |  |
| (% children with ARI receiving antibiotics) |  | 0.957 |  | - - |
|  |  |  |  |  |
| Vitamin A supplementation |  | -0.016 (-0.179;0.146) |  |  |
| (% children) |  | 0.843 |  | - - |
|  |  |  |  |  |
| **Unhealthy household environment** | | | | |
| Urbanization |  | -0.548 (-0.635;-0.459) |  | -0.441 (-0.621;-0.26) |
| (% of urban population) |  | <0.0001 |  | <0.0001 |
|  |  |  |  | 0.14 |
| Open defecation |  | 0.338 (0.065;0.610) |  | 0.236 (0.032;0.44) |
| (% of population ) |  | 0.015 |  | 0.024 |
|  |  |  |  | 0.003 |
| Improved drinking water source |  | -0.031 (-0.131;0.069) |  |  |
| (% of population) |  | 0.551 |  | - - |
|  |  |  |  |  |
| **% of residual variance explained by covariables^3^** |  |  |  | **42%** |
| **Level 1 Model - Proximal: Individual-level variables^e^** | | | | |
| **Infections** | | | | |
| ARI infection |  | -0.012 (-0.225;0.201) |  |  |
| (% children within last 2 weeks) |  | 0.909 |  | - - |
|  |  |  |  |  |
| Diarrhoea infection |  | 0.132 (-0.139;0.403) |  |  |
| (% children within last 2 weeks) |  | 0.341 |  | - - |
|  |  |  |  |  |
| **Child characteristics** | | | | |
| Low birthweight |  | -0.121 (-0.583;0.341) |  |  |
| (% children) |  | 0.608 |  | - - |
|  |  |  |  |  |
| **Maternal characteristics** | | | | |
| Anemia |  | 0.022 (-0.174;0.218) |  |  |
| (% women 15-49 years) |  | 0.829 |  | - - |
|  |  |  |  |  |
| Young mother births (<18 years) |  | 0.221 (-0.543;0.985) |  |  |
| (% births within last 5 years) |  | 0.571 |  | - - |
|  |  |  |  |  |
| BMI |  | -2.129 (-5.179;0.919) |  |  |
| (mean, mothers 15-49 years) |  | 0.171 |  | - - |
|  |  |  |  |  |
| Height |  | -1.285 (-3.030;0.460) |  |  |
| (mean, mothers 15-49 years) |  | 0.149 |  | - - |
|  |  |  |  |  |
| Interpregnancy interval |  | 0.161 (-0.083;0.404) |  |  |
| (median, mothers 15-49 years) |  | 0.196 |  | - - |
|  |  |  |  |  |
| Age |  | -1.617 (-2.795;-0.439) |  | -1.552 (-2.702;-0.402) |
| (mean, mothers 15-49) |  | 0.007 |  | 0.008 |
|  |  |  |  | 0.08 |
| Total fertility rate |  | 2.291 (-0.429;5.011) |  |  |
| (mean, mothers 15-49 years) |  | 0.099 |  | - - |
|  |  |  |  |  |
| **% of residual variance explained by covariables^3^** |  |  |  | **42%** |
| **Time** | | | | |
| **Time** | | | | |
| Year |  | -0.722 (-1.112;-0.328) |  | 0.077 (-0.509;0.662) |
|  |  | <0.0001 |  | 0.798 |
|  |  |  |  | 0.001 |

| Note: Variables significant at p<0.20 in bivariate analysis were entered into linear multilevel mixed-effects models. | |
| --- | --- |
| ^1^ Variables in each group are adjusted for all other variables in the same group or above. |  |
| ^2^ Calculated using Cohen’s F^2^ to estimate effect size as ([R^2^ of the full model – R^2^ of the restricted model]/[1 - R^2^ of the full model]) with the restricted model excluding the variable to be evaluated (without the effect of interest) and with the random intercept variance constrained to be the same as the full model, and defined as: small effect with F^2^ ≥ 0.02, medium effect with F^2^ ≥ 0.15, and large effect with F^2^ ≥ 0.35 (Cohen J.E., 1988). | |
| ^3^ Calculated as ([residual variance component of null model - residual variance covariable model]/[residual variance null model]), with the null model being (outcome and department). | |
| ^4^Comprehensive Health Insurance “Seguro Integral de Salud” (SIS, acronym in Spanish). | |
| ^5^Articulated Nutrition Program (PAN, acronym in Spanish) | |
| ^6^Strategic Program of Maternal and Neonatal Health (PSMN, acronym in Spanish) | |
| ^7^Composite coverage index: Summary measure of intervention coverage along the reproductive, maternal, newborn, and child health continuum. It is a weighted mean of the coverage for interventions from 4 domains: i) reproductive services (family planning coverage), ii) maternal and newborn care (antenatal care and skilled birth attendant), iii) immunization (BCG, three doses of DPT3, and measles vaccines), and iv) management of illness (ORT and care seeking for pneumonia). | |
| ^a^ Given that we considered a two-year time lag between predictors and outcome, our units of analysis are: 360 for the full time period (24 departments x 15 years, 2000 to 2016), 192 for the pre-PAN time period (24 departments x 8 years, 2000 to 2007), and 168 for the post-PAN time period (24 departments x 7 years, 2008 to 2016). | |
| ^b^ Level 4 multivariable model includes all statistically significant (p<0.1) distal variables as listed. | |
| ^c^ Level 3 multivariable model includes level 4 model + all statistically significant (p<0.1) intermediate I variables as listed. | |
| ^d^ Level 2 multivariable model includes level 4 model + 3 model + all statistically significant (p<0.1) intermediate II variables as listed. | |
| ^e^ Level 1 multivariable model includes level 4 model + 3 model + 2 model + all statistically significant (p<0.1) proximal variables as listed. | |

## **Supplementary Appendix Table 13:** Multilevel linear models for under-5 stunting prevalence (2 years’ time-lag), period 2008-2016

**factors statistically significant at p<0.05 are highlighted in red*

| **Domain/Indicator** | **Outcome = Stunting** | | | |
| --- | --- | --- | --- | --- |
|  | **(Percentage of stunting among under-5 children^1^)** | | | |
|  | **Period 2008-2016^a^** | | | |
|  | **Time-adjusted regression coefficient** | | **Time- and confounder-adjusted^1^** | |
|  |  |  | **regression coefficient** | |
|  |  | b estimate (95% CI) |  | b estimate (95% CI) |
|  |  | *p*-value |  | *p*-value |
|  |  |  |  | Effect size^2^ |
| **Level 4 Model - Distal: National level & Macro variables^b^** | | | | |
| **Basic causes & Income poverty** | | | | |
| Log Gross domestic product (GDP) per capita |  | -10.139 (-14.156;-6.612) |  |  |
| (USD/person) |  | <0.0001 |  | - - |
|  |  |  |  |  |
| Poverty line |  | 0.394 (0.242;0.546) |  | 0.394 (0.242;0.546) |
| (% families below) |  | <0.0001 |  | <0.0001 |
|  |  |  |  | 0.15 |
| Gini coefficient for income  0 (perfect equality) – 1 (perfect inequality) |  | 0.043 (-0.267;0.354) |  |  |
|  |  | 0.785 |  | - - |
|  |  |  |  |  |
| Median years of schooling among |  | -1.450 (-2.501;-0.399) |  |  |
| women 15 years or older |  | 0.007 |  | - - |
|  |  |  |  |  |
| **% of residual variance explained by covariables^3^** |  |  |  | **55%** |
| **Level 3 Model - Intermediate I: Nutrition sensitive and specific programs^c^** | | | | |
| **Nutrition specific and sensitive programs** | | | | |
| Conditional cash transfer “Juntos” program |  | -0.002 (-0.027;0.022) |  |  |
| (No. families/1000 rural population |  | 0.854 |  | - - |
|  |  |  |  |  |
| SIS^4^ coverage |  | 0.055 (-0.019;0.129) |  |  |
| (No. under-5 attendances/under-5 population) |  | 0.147 |  | - - |
|  |  |  |  |  |
| Articulated Nutrition Program (PAN^5^ log expenditure, per capita) |  | -0.243 (-0.544;0.058) |  |  |
| (USD/person) |  | 0.113 |  | - - |
|  |  |  |  |  |
| Strategic Program of Maternal and Newborn Health (PSMN^6^ log expenditure, per capita |  | -0.252 (-0.449;0.054) |  |  |
| (USD/person) |  | 0.107 |  | - - |
|  |  |  |  |  |
| **% of residual variance explained by covariables^3^** |  |  |  | **- -** |
| **Level 2 Model - Intermediate II: Community and Household-level variables^d^** | | | | |
| **Inadequate health services** | | | | |
| DPT vaccine |  | 0.004 (-0.085;0.093) |  |  |
| (% infants with 3 doses) |  | 0.929 |  | - - |
|  |  |  |  |  |
| Measles vaccine |  | -0.005 (-0.078;0.068) |  |  |
| (% infants) |  | 0.894 |  | - - |
|  |  |  |  |  |
| Pneumococcal vaccine |  | -0.033 (-0.062;-0.003) |  |  |
| (% infants with vaccine during first 1 year) |  | 0.029 |  | - - |
|  |  |  |  |  |
| Rotavirus vaccine |  | -0.023 (-0.051;0.004) |  |  |
| (% infants with vaccine during first 1 year) |  | 0.095 |  | - - |
|  |  |  |  |  |
| **Composite Coverage** | | | | |
| Composite coverage index^6^ |  | -0.167 (-0.354;0.019) |  |  |
|  |  | 0.079 |  | - - |
|  |  |  |  |  |
| **Reproductive and maternal care** | | | | |
| Skilled attendant for last birth |  | -0.268 (-0.407;-0.129) |  | -0.178 (-0.288;-0.068) |
| (% mothers) |  | <0.0001 |  | 0.002 |
|  |  |  |  | 0.06 |
| 4+ antenatal care visits for last birth |  | 0.021 (-0.301;0.343) |  |  |
| (% mothers) |  | 0.897 |  | - - |
|  |  |  |  |  |
| **Human resources for health** | | | | |
| Density of human resources for health |  | 0.140 (0.040;0.240) |  |  |
| (Doctors, nurses and midwives per 10,000 |  | 0.006 |  | - - |
| population) |  |  |  |  |
| **Care seeking interventions for childhood illness & child micronutrient intake** | | | | |
| Care seeking for pneumonia |  | 0.041 (-0.008;0.091) |  | 0.04 (-0.002;0.082) |
| (% children with pneumonia seeking care) |  | 0.099 |  | 0.062 |
|  |  |  |  | 0.03 |
| Oral rehydration therapy |  | -0.061 (-0.130;0.007) |  |  |
| (% children with diarrhea receiving ORT) |  | 0.08 |  | - - |
|  |  |  |  |  |
| ARI receiving antibiotic treatment |  | 0.001 (-0.221;0.223) |  |  |
| (% children with ARI receiving antibiotics) |  | 0.993 |  | - - |
|  |  |  |  |  |
| Vitamin A supplementation |  | -0.002 (-0.121;0.117) |  |  |
| (% children) |  | 0.977 |  | - - |
|  |  |  |  |  |
| **Unhealthy household environment** | | | | |
| Urbanization |  | -0.401 (-0.481;-0.321) |  | -0.202 (-0.274;-0.131) |
| (% of urban population) |  | <0.0001 |  | <0.0001 |
|  |  |  |  | 0.04 |
| Open defecation |  | 0.463 (-0.050;0.976) |  |  |
| (% of population ) |  | 0.077 |  | - - |
|  |  |  |  |  |
| Improved drinking water source |  | -0.191 (-0.344;-0.038) |  |  |
| (% of population) |  | 0.014 |  | - - |
|  |  |  |  |  |
| **% of residual variance explained by covariables^3^** |  |  |  | **63%** |
| **Level 1 Model - Proximal: Individual-level variables^e^** | | | | |
| **Infections** | | | | |
| ARI infection |  | 0.193 (0.049;0.337) |  | 0.165 (0.064;0.266) |
| (% children within last 2 weeks) |  | 0.009 |  | 0.001 |
|  |  |  |  | 0.10 |
| Diarrhoea infection |  | 0.121 (-0.044;0.286) |  |  |
| (% children within last 2 weeks) |  | 0.151 |  | - - |
|  |  |  |  |  |
| **Child characteristics** | | | | |
| Low birthweight |  | -0.043 (-0.547;0.460) |  |  |
| (% children) |  | 0.866 |  | - - |
|  |  |  |  |  |
| **Maternal characteristics** | | | | |
| Anemia |  | 0.013 (-0.092;0.118) |  |  |
| (% women 15-49 years) |  | 0.804 |  | - - |
|  |  |  |  |  |
| Young mother births (<18 years) |  | 0.037 (-0.309;0.382) |  |  |
| (% births within last 5 years) |  | 0.835 |  | - - |
|  |  |  |  |  |
| BMI |  | -1.241 (-2.581;0.099) |  |  |
| (mean, mothers 15-49 years) |  | 0.07 |  | - - |
|  |  |  |  |  |
| Height |  | -2.649 (-4.749;-0.549) |  |  |
| (mean, mothers 15-49 years) |  | 0.013 |  | - - |
|  |  |  |  |  |
| Interpregnancy interval |  | -0.187 (-0.341;-0.032) |  |  |
| (median, mothers 15-49 years) |  | 0.018 |  | - - |
|  |  |  |  |  |
| Age |  | -1.008 (-2.395;0.379) |  |  |
| (mean, mothers 15-49) |  | 0.154 |  | - - |
|  |  |  |  |  |
| Total fertility rate |  | 6.876 (3.696;10.055) |  | 4.093 (1.095;7.092) |
| (mean, mothers 15-49 years) |  | <0.0001 |  | 0.007 |
|  |  |  |  | 0.05 |
| **% of residual variance explained by covariables^3^** |  |  |  | **71%** |
| **Time** | | | | |
| **Time** | | | | |
| Year |  | -1.470 (-1.923;-1.017) |  | -0.46 (-0.777;-0.143) |
|  |  | <0.0001 |  | 0.004 |
|  |  |  |  | 0.99 |

| Note: Variables significant at p<0.20 in bivariate analysis were entered into linear multilevel mixed-effects models. | |
| --- | --- |
| ^1^ Variables in each group are adjusted for all other variables in the same group or above. |  |
| ^2^ Calculated using Cohen’s F^2^ to estimate effect size as ([R^2^ of the full model – R^2^ of the restricted model]/[1 - R^2^ of the full model]) with the restricted model excluding the variable to be evaluated (without the effect of interest) and with the random intercept variance constrained to be the same as the full model, and defined as: small effect with F^2^ ≥ 0.02, medium effect with F^2^ ≥ 0.15, and large effect with F^2^ ≥ 0.35 (Cohen J.E., 1988). | |
| ^3^ Calculated as ([residual variance component of null model - residual variance covariable model]/[residual variance null model]), with the null model being (outcome and department). | |
| ^4^Comprehensive Health Insurance “Seguro Integral de Salud” (SIS, acronym in Spanish). | |
| ^5^Articulated Nutrition Program (PAN, acronym in Spanish) | |
| ^6^Strategic Program of Maternal and Neonatal Health (PSMN, acronym in Spanish) | |
| ^7^Composite coverage index: Summary measure of intervention coverage along the reproductive, maternal, newborn, and child health continuum. It is a weighted mean of the coverage for interventions from 4 domains: i) reproductive services (family planning coverage), ii) maternal and newborn care (antenatal care and skilled birth attendant), iii) immunization (BCG, three doses of DPT3, and measles vaccines), and iv) management of illness (ORT and care seeking for pneumonia). | |
| ^a^ Given that we considered a two-year time lag between predictors and outcome, our units of analysis are: 360 for the full time period (24 departments x 15 years, 2000 to 2016), 192 for the pre-PAN time period (24 departments x 8 years, 2000 to 2007), and 168 for the post-PAN time period (24 departments x 7 years, 2008 to 2016). | |
| ^b^ Level 4 multivariable model includes all statistically significant (p<0.1) distal variables as listed. | |
| ^c^ Level 3 multivariable model includes level 4 model + all statistically significant (p<0.1) intermediate I variables as listed. | |
| ^d^ Level 2 multivariable model includes level 4 model + 3 model + all statistically significant (p<0.1) intermediate II variables as listed. | |
| ^e^ Level 1 multivariable model includes level 4 model + 3 model + 2 model + all statistically significant (p<0.1) proximal variables as listed. | |

# **Supplementary Appendix 6:** Programs and Policies

## **Supplementary Appendix Table 14:** Description of Acts/Law/Regulations, Policies, and Programs from 1995-2016

| **POLICIES/STRATEGIES/PLANS** | | |
| --- | --- | --- |
| Social assistance policies  (1980 – 2005) | Description | Social assistance policies began in the 1980s as an attempt by the Government of Peru to reduce food insecurity and malnutrition through food supplementation programs. These programs included *Comedores populares*/ “Community kitchens” and *Vaso de Leche/ “*Glass of Milk.” By 2000, over half (59%) of Peru’s food assistance budget was going towards these two programs [6]. In 2001, social assistance policies were reformed when President Alejandro Toledo took office and prioritized the reduction of poverty and the improvement of social justice. Literature indicates that many of these social assistance policies did not have a positive impact on reducing child malnutrition or stunting rates [6,12,93]. Among the reasons given are that they were poorly designed with little collaboration between government sectors, had insufficient funds to reach everyone, and that the programs did not focus on many of the communities with the highest prevalence of stunting [12]. Despite this, social assistance polices did lead to the establishment of other programs that were effective in reducing wealth inequalities and strengthening the health system, which have been linked to declines in stunting in Peru. |
|  | Importance | Likely important |
| Health sector reform policies  (1995 – 2004) | Description | These policies were developed to eliminate barriers and to improve the accessibility of healthcare in Peru. The policies resulted in two health reform programs being developed, PARSALUD I and II. These two programs are described in detail below [93,111]. USAID, PRISMA and the Government of Peru collaborated to reform Peru’s health sector to reduce maternal and infant mortality rates [93]. Primary-level health service reform included the introduction of two major competing reform strategies (the Basic Health For All program and the Local Committees for Health Administration/CLAS program). Following this, there was a shift to focusing on the social security health system [111]. |
|  | Importance | Likely important |
| Strengthened civil society and stakeholder advocacy  (2001 – present) | Description | Beginning in 2001 through interim President Valetin Paniagua’s creation of the Roundtable for Poverty Reduction, the state and civil society began working together on poverty reduction initiatives with the aim of reforming social policy, improving service delivery, and including civil society to a greater degree in the design, decision making and financial planning of social policies. In early 2006, civil society organizations played an important role in the formation of the Child Malnutrition Initiative. In addition, civil society groups played a key role in designing and implementing both CRECER and JUNTOS [6,12]. As many of the programs that resulted from this period of strengthened civil society and stakeholder advocacy played a role in the reduction of stunting in Peru, this period can be seen as having an indirect role in stunting reduction. |
|  | Importance | Very important |
| National agreement and political agenda  (2002 – present) | Description | The National Agreement is a long-term forum for national dialogue that was first signed in 2002 by government representatives, political parties, and civil society organizations. The National Agreement is committed to democracy, and reducing inequalities and poverty in Peru [93]. This was successful because it effectively reduced the number of acting government members involved in policy and decision-making. The establishment of the National Agreement was instrumental to the reaching of a consensus between diverse political parties. It established long-term policies that were implemented across different governments [10,93,111,112]. The Agreement led to the formation of the Child Malnutrition Initiative. In 2005, the National Agreement included objectives of the Roundtable Against Poverty [111]. These were 11 priority actions to support child development and reduce stunting. The roundtable against poverty also focuses on equity and gender, better efficiency in the implementation of programs for the fight against poverty, improving transparency, and involve citizen in all stages of state social policies [111]. |
|  | Importance | Very important as precursor to CMI initiative |
| Strengthened antipoverty policies  (2005 – present) | Description | These policies were proposed by the Toledo government in 2005 to provide cash disbursements to families living in extreme poverty, with the condition that children attend school and pregnant women regularly access health services [113]. Initial policies were rejected by congress and state officials and improved upon, eventually leading to the implementation of JUNTOS. This antipoverty program is described in further detail below [14]. |
|  | Importance | Very important |
| Child Malnutrition Initiative  (2005/6 – present) | Description | The Child Malnutrition Initiative (CMI) is a civil society platform that was created to advocate for effective nutrition action during a period of time when funding for nutrition was decreasing [12]. The CMI was effective because it went beyond feeding programs and recognized the need for broader interventions to reduce stunting. These included interventions related to improved hygiene practices, improving micronutrient supplies, promoting community health, boosting income, and improving food availability. The CMI built on previous examples from UNICEF and CARE on successful reductions in stunting [12]. Data from the qualitative analysis indicates that the CMI was instrumental in achieving the commitment of political candidates in the fight against stunting [12,32,33]. |
|  | Importance | Very important |
| Results and performance-based policies in education and health (2007 – present) | Description | This is a national initiative established in 2007 and operated by the Ministry of Finance to fight malnutrition through Results Based Budgeting. The government developed budgets based on the relationship between program funding levels and expected results. The aim of this strategy was to ensure appropriate allocation of funds and effective of public spending [10,114,115]. These policies were coordinated with government ministries and social programs involving health and education to provide public services and attain complementary goals [33]. |
|  | Importance | Very important |
| Cross-cutting antipoverty and social inclusion policies  (2009 – present) | Description | This is a group of policies aimed at reducing poverty and improving equality in Peru. These policies were placed under the Ministry of Development and Social Inclusion, which coordinated the reduction of stunting throughout the country [10]. Cross-cutting antipoverty strategies and social inclusion policies evolved over time and led to progress in child health and nutritional status. These programs focus on economic growth, welfare, and the health of the poor. Programs such as Incluir para crecer, described in further detail below, were included under these policies [10,32,93,116]. |
|  | Importance | Very important |
| Universal Healthcare Commitment (2009 – present) | Description | The Universal Healthcare Commitment (UHC) was created with the goal of expanding Seguro Integral de Salud (SIS), also known as Comprehensive Health Insurance, throughout Peru. This regulatory framework aims to achieve universal healthcare coverage by means of a three tiered system of fully subsidized health insurance for the poor, semi-contributory insurance for informal and small-business workers and contributory insurance [117]. This is in line with the goal of Universal Health Insurance (AUS) for all Peruvians so that they may access quality health services. The Universal Healthcare Commitment was created from coordinated institutional efforts between the Peruvian Health Social Security Office (EsSalud), the Comprehensive Health Insurance scheme (Seguro Integral de Salud/SIS), the Ministry of Health (MINSA), and regional governments [117]. |
|  | Importance | Likely important |

| **PROGRAMS** | | |
| --- | --- | --- |
| Vaso de Leche (VdL)  (1980s – 2000) | Description | Vaso de Leche, or the “Glass of Milk” program, was a food supplementation program that aimed to provide milk and milk substitutes to children under 6 years of age in targeted at-risk households in Peru [118]. Initially the program sought to reach children <6 years of age as well as pregnant and lactating women, however by 1998 the program had reached 44% of households with children ages 3-11 years [4]. Data from our qualitative synthesis indicates that food distribution programs in Peru were unsustainable, and that drivers of stunting went beyond lack of food. Similarly, the literature indicates that feeding programs were largely ineffective within the Peruvian context. Further information on school feeding programs is in appendix 15. However, findings also indicate that these programs led to greater investments in areas with high rates of stunting, with a particular focus on results and targeting the basic and underlying causes of malnutrition. While the Vaso de Leche program itself may not have contributed to an immediate decline in stunting, it led to the establishment of other programs that were effective in reducing wealth inequalities and strengthening the health system, both of which led to declines in stunting. |
|  | Importance | Unclear but could have had some impact |
| National family planning programs (1985 – 2000) | Description | These were a series of family planning programs and policies beginning in 1985, initiated when the Government of Peru passed the National Population Policy giving individuals the right to freely determine the number and spacing of their children [119]. Beyond the National Population Policy, these initiatives included: 1) the first National Family Planning Program (1987-1990), aimed at decreasing the fertility rate to 2.5 births per woman by 2000; 2) the second National Family Planning Program (1991-1995), aimed at expanding access to health services in rural areas; 3) the 1995 Ministry of Health policy making contraception free through government institutions; 4) the National Program for Reproductive Health and Family Planning (1996-2000) which included voluntary surgical contraception, and 5) the ReproSalud project (1996-2005) which was funded by USAID and consisted of a communication campaign that developed information, education, and improved program delivery for reproductive health [119]. Available data from this time period shows an increase in the use of modern family planning methods among married women between 1986 and 2000, as well as a reduction in the total fertility rate in Peru between 1990 and 2011 [111]. |
|  | Importance | Insufficient evidence but could have had some impact |
| Reprosalud  (1996– 2005) | Description | ReproSalud was a reproductive health project funded by USAID and run by women’s advocacy organization Movimiento Manuela Ramos (MMR). ReproSalud focused on women’s rights and empowerment through a participatory approach, working with women’s groups at the community level to build and execute projects based on identified reproductive health needs within the target community. The project focused on poor rural and peri-urban areas within Peru and typically covered three themes: reproductive health, income generation and advocacy, though the primary focus of all projects was reproductive health [120]. Data collected from some of the ReproSalud project sites indicated an increased uptake of family planning services between 2 and 4 times that of the baseline, as well as a reduction in instances of domestic violence. The impact of this program in beneficiary communities compared to non-beneficiary communities was not reported. |
|  | Importance | Insufficient evidence but could have had some impact |
| Child vertical programs  (1990 – 1995) | Description | These are a group of vertical intervention programs that targeted reproductive, maternal, neonatal, and child health [93]. These programs were focused on acute respiratory infection (ARI), acute diarrheal disease and immunizations. Recently, child vertical programs were consolidated, leading to the creation of Integrated Management of Childhood Illness (IMCI). These child vertical programs marked the beginning of a shift in focus to child survival that included mothers and newborns [93,115,121]. The impact of these programs on stunting were not reported on in either the qualitative report or in the literature. |
|  | Importance | Insufficient evidence to comment on relation to stunting but likely important to overall child survival |
| Friends of the Mother and Child Hospital Initiative  (1995 – 2002) | Description | Friends of the Mother and Child Hospital Initiative is based on the Baby Friendly Hospital Initiative, which has shown to be effective at improving breastfeeding rates and practices while reducing infant morbidity and mortality. Created in 1991 by WHO and UNICEF, the Baby Friendly Hospital Initiative (BFHI) aims to protect, promote and support breastfeeding in facilities where maternity services are provided by ensuring that the facilities follow the WHO/UNICEF “Ten Steps to Successful Breastfeeding” and adhere to the 1981 International Code of Marketing of Breast-milk Substitutes. According to PAHO/WHO data, only 8 of the 509 eligible health facilities in Peru have become Baby Friendly Hospital Initiative certified, all of which occurred after 2008. |
|  | Importance | Not important to national stunting decline given scale of program |
| Integrated Management of Childhood Illness  (1996 – present) | Description | The Integrated Management of Childhood Illness (IMCI) program in Peru is based on the World Health Organization’s IMCI strategy, which is an approach to child health that targets children <5 and aims to reduce death, illness, and disability while improving both growth and development. The program aims to improve infrastructure, train community health workers, and deliver necessary health interventions, with education being an important component [122]. Constraints to scaling up IMCI in Peru have been linked to low female literacy rates, lack of an official IMCI policy, no specific budget for the program, and high staff turnover [123]. Though IMCI was introduced nationally in Peru, its coverage has not been adequate which has limited the potential impact of this program. In addition, issues have arisen with health service accessibility, preventing access to IMCI in some areas [65]. This program was not mentioned in qualitative report. |
|  | Importance | Insufficient evidence |
| Maternal and Child Health Insurance (1998-2001) | Description | Maternal and Child Health Insurance was a program that was initiated in 1998, targeting the Peru’s poorest pregnant women and children under 5 in order to improve their health outcomes [93]. No comprehensive evaluations of this insurance program were found in the literature, though one external evaluation showed that mothers with this insurance were twice as likely as those without the insurance to have delivered their last child in an Emergency Obstetric Care facility. This evaluation also found that coverage was an issue, as was health care infrastructure necessary to meet beneficiary demand [124]. In 2002, this program evolved into the more broad-based Comprehensive Health Insurance (SIS) program [93]. |
|  | Importance | Insufficient evidence—may be important as it lay the groundwork for SIS |
| Good Start in Life  (1999 – 2008) | Description | The main objectives of the Good Start in Life program were to prevent stunting in children <3 years of age and to improve healthcare for pregnant and/or lactating women [111]. The program aimed to accomplish this through education and behaviour change among caregivers [125]. In 2004, the program had reached approximately 75,000 children <3 years and 35,000 pregnant and/or lactating women [111]. It has been reported that the rate of stunting declined from 54% to 37%, iron deficiency decreased from 76% to 52%, and vitamin A deficiency decreased from 30% to 5% among children in the regions were the program was implemented[111]. In the qualitative report, participants mentioned that the Articulated Nutrition Program (PAN) was built from experience gained through the implementation of the Good Start in Life Program. |
|  | Importance | Likely important |
| Health sector reform programs (PARSALUD I/II)  (2000 – 2015) | Description | PARSALUD I was initially created in 2000 to improve health services in general, but was then redesigned in 2003 with an aim to improve maternal care in Peru through improvements in infrastructure, equipment and human resources for health [126]. The PARSALUD II project was launched in 2009, with the same design as the reformed PARSALUD I program but was focused on reducing chronic undernutrition in children in the nine poorest regions in Peru [111]. Based on existing literature, in-country interviews and focus group discussions, the impact of PARSALUD I/II on stunting is unclear, though it could be hypothesized it had downstream effects on child growth. |
|  | Importance | Likely important |
| Comprehensive health insurance system (SIS)  (2002 – present) | Description | The Comprehensive Health Insurance System (SIS) evolved from the Maternal and Child Health Insurance program that ran from 1998-2001, targeting mothers and children under 5. Launched in 2002, the main objective of SIS is to increase utilization of health services by removing barriers to accessing care for poor people/vulnerable populations, especially women and children under 5. The program involves a targeting procedure whereby potential beneficiaries are entitled to either fully or partially subsidized access depending on their income [93]. Evidence from the literature shows that SIS improved access to health care for the poor and those living in rural areas [93]. Quantitative data analysis also indicates SIS was associated with a small decrease in stunting during from 2000 to 2016, and qualitative data supports that this program was important to reducing stunting in Peru. |
|  | Importance | Very important |
| The Sustainable Network for Food Security Program (REDESA)  (2002 – 2006) | Description | The main objective of the REDESA program was to improve food security for families in the rural Andes by increasing family income by 20% and improving the health and nutrition conditions of beneficiaries through a 30% reduction in diarrhea. The these program objectives were measured by the reduction of chronic malnutrition in children <3 years of age [127]. The REDESA program was implemented by CARE in the communities of Ancash, Apurímac, Ayacucho, Cajamarca, Huancavelica and Puno. An evaluation of this food security program indicated a stunting decline of 9.9% over the study period [128]. REDESA seems to be an important program based on the Peru literature and interviews with key stakeholders. It was shown to have positively influenced rates of exclusive breastfeeding, proper feeding practices during instances of diarrhea, and aimed to reduce diarrhea and illness overall [128] . Additionally, the program was only piloted in six departments so likely did not have national level impact. |
|  | Importance | Likely important |
| National Strategy on Sexual and Reproductive Health  (2004 – present) | Description | This strategy targets all stages of the lifespan (intrauterine through older adulthood) to improve the sexual and reproductive health of the population of Peru. Initiated in 1994 when Peru signed the Program of Action following the International Conference on Population and Development, it was reformed into the current National Strategy in 2004. This strategy is focused on improving gender equality and cultural sensitivity in care delivery, improving response to obstetric and neonatal emergencies, preventing unwanted pregnancies, and reducing transmission of HIV/STIs [129]. |
|  | Importance | Insufficient information |
| Rural Retention Program (SERUMS) (1985 – present) | Description | SERUMS is a decentralized human resources for health (HRH) plan aimed at distributing and retaining health workers providing care in remote areas of Peru. The program was developed with a guide outlining best practices and procedures for implementing a retention policy [130]. SERUMS consists of recent graduates of health care programs (e.g. doctors, nurses, midwives, dentists), who perform one year of rural service delivery after graduation. In terms of retention, the goal is to keep SERUMS participants working in rural areas after their initial placement. Recently, the Ministry of Health increased the salary of health workers in rural areas to promote this retention [129]. The literature reports that SERUMS has improved the number of health professionals working in Apurimac, Huancavelica, and other poor areas of the country. There is a larger number of physician graduates (~100%) that enter SERUMS compared to midwives (85%) and nurses (62%) [131,132]. Despite this, the literature reports that following the completion of SERUMS there is a low number of healthcare professionals that enter the Ministry of Health system [133]. Results from our quantitative analysis show that the density of HRH increased from 9.04 to 11.78 to 28.24 doctors, midwives and nurses per 10,000 population from 2000-2016. Our analysis suggests that strengthened HRH may have contributed to stunting decline in the earlier period, though during the latter period of rapid change, its effect did not push through as significant, meaning that other factors were more important than HRH in the stunting decline. |
|  | Importance | Likely important |
| JUNTOS  (2005 – present) | Description | JUNTOS (“Together”) is a conditional cash transfer program aimed at alleviating poverty by positively influencing health seeking behaviors and education among women. Mothers enrolled in JUNTOS are required to bring their children under age 2 years to health centers to monitor their growth and overall health, and they are also required to enroll their children (6 – 19 years) in school. In return, these mothers receive 200 Peruvian Soles every 2 months [6,7,12,93]. The evidence of the impact of JUNTOS on stunting prevalence in Peru has been mixed [16,134]. However, the program was effective in targeting poor households with children <12 and was shown to have increased the utilization of health services [6,93,135,136]. |
|  | Importance | Likely very important but longer follow-up duration required |
| Food security policies  (2006 – 2008) | Description | These were a set of policies implemented by the Ministry of Agriculture to reduce food insecurity in Peru. The aim of these policies was to reduce micronutrient deficiencies, malnutrition, and to promote healthy food consumption. These food security policies eventually led to the implementation of CRECER and a improvements in food security [137] . These policies were also focused on preventing the risk of nutritional deficiencies and reducing the levels of undernourishment among families with children under five years of age, expectant mothers and other vulnerable segments of the population [138] . |
|  | Importance | Likely important |
| 5 x 5 x 5 campaign  (2006 – 2011) | Description | The 5 x 5 x 5 campaign was a pledge signed by presidential candidates during the 2006 election to reduce chronic malnutrition by 5% in children under 5 years over the course of 5 years. President Alan Garcia won the election and pledged to reduce child malnutrition by 9% by focusing on children under-3 years and closing the urban-rural gap [6,12]. The 5 x 5 x 5 campaign also led to the engagement of the *Presidentes Regionales*, which included presidents of all 25 regions of the country, who signed the Lima Declaration on Child Malnutrition which committed them all to reducing chronic malnutrition in children by 5% by 2011 [12]. |
|  | Importance | Likely very important but difficult to measure |
| CRECER  (2007 – 2011) | Description | CRECER (“Grow”) was a comprehensive national intervention strategy for promoting, facilitating, and executing a set of poverty reduction and social inclusion programs, combining several initiatives to be managed under the guidance of the Prime Minister’s Office. The program was focused on spending in the poorest communities [10]. Initially, CRECER began in just 200 of the 1800 districts in Peru. However by 2011, CRECER existed in approximately 900 districts [33]. This program was not explicitly mentioned in our qualitative analyses, but findings from the literature show that the program was successful as it resulted in a shift in government thinking towards nutrition, food security, and chronic malnutrition [12]. In addition, it led to the establishment of Incluir para crecer which is still in place today and focuses on improved health and nutrition across the lifespan [111]. Therefore, Given CRECER's function as a consolidation of programs and re-prioritization of funding (including JUNTOS), it was likely effective at streamlining focus to important target areas and thus reduced poverty and in turn, may have improved nutrition. |
|  | Importance | Important as consolidation of several nutrition-focused initiatives |
| Maternal-Neonatal Strategic Program (2007 – present) | Description | The Maternal-Neonatal Strategic Program was created with the intention of addressing the need for universal attention to child and adolescent health [139]. This program focuses on providing family planning services, offering quality prenatal care, identifying risk factors in expectant mothers and ensuring delivery and care of newborns is performed by qualified health personnel[139]. The program strengthened the basic obstetric care services as well as access and availability of safe blood for delivery [139]. This program also provided funding towards Comprehensive Health Insurance (SIS) to increase coverage for women and children and increased the budget for the operation and equipment of neonatal intensive care units across the country [139]. . |
|  | Importance | Very important |
| Articulated Nutrition Program  (2007 – present) | Description | The Articulated Nutrition Program (PAN)—also known as the Joint Nutritional Strategic Program—was created by the Ministry of Finance in 2008. As a part of the Results Based Budgeting, PAN uses a multi-factorial framework that targets the drivers of stunting. In 2010, PAN’s budget was increased and funds allocated towards the regions of Peru with the highest rates of malnutrition. Specifically, funds were directed towards pneumococcus and rotavirus vaccinations for children; reducing iron deficiency among children and pregnant women; and preventative health and nutrition services to ensure adequate child growth and prevention of growth faltering [10,33]. PAN also seeks to improve operational capacity (e.g. human resources, supplies, medicines and equipment) in order to address diarrhea and respiratory infections in children, and to increase the allocation of funds for children’s healthcare coverage [140]. Additionally, the program implemented the dispersion of education, information and communication services aimed at families, schools and communities in order to improve hygiene practices in food preparation [140].There was also training for mothers (e.g. counseling, demonstration sessions and educational sessions) on exclusive breastfeeding, child feeding and care for sick children, all through the Growth and Development Control (CRED) program [140].Surveillance and improvements to the quality of drinking water through chlorification were undertaken as well as improvements in waste removal and development of water and basic sanitation projects for rural áreas [140]. This program also included the delivery of materials to reduce intra-household pollution as well as provide supplementation of particular micronutrients (i.e. ferrous sulfate and Vitamin A) [140]. |
|  | Importance | Very important |
| Implementation of the Universal Healthcare Commitment  (2009 – present) | Description | This program refers to a set of actions that took place to fulfill the commitment of Universal Health Insurance (AUS) in Peru. In 2009, the Universal Health Insurance Framework Law was approved, guaranteeing all Peruvians the ability to access health and disease interventions regardless of their wealth status [93,141] . More information on the Universal Health Commitment can be found above. |
|  | Importance | Likely important |
| Incluir para crecer (CRECER II)  (2011 – present) | Description | Incluir para crecer evolved from the earlier CRECER program (2007–2011) with an ambitious goal of reducing chronic malnutrition by 13% over a 5 year period through a lifecycle approach [10]. The new program aims to reduce health gaps and provide interventions for at-risk and vulnerable populations in Peru. It differs from the original CRECER program by targeting the entire lifespan (early infancy through old age), and by harmonizing various government programs. Another difference between Incluir para crecer and CRECER is that the new program includes a focus on five additional health outcomes [111]. It is a national-level program that targets 4.8 million people living in mostly urban or marginalized areas [142]*.* Unfortunately data on the association between stunting and Incluir para crecer was unavailable in both the literature review and in the qualitative report. |
|  | Importance | Likely important |

# **Supplementary Appendix 7:** Qualitative Results

We present separately in the following sections the results obtained through the FGD and the in-depth interviews. The aim is to extract any differential findings emerging from each activity, as well as to identify common and consistent messages. Results of the FGD are presented first to provide a wide perspective of determinants of stunting reduction as well as a description of the context. We then present the results of the in-depth interviews.

**Focus Group Discussion**

A main message that emerged from the FGD was that the rapid decline in stunting began in 2007, but efforts to reduce stunting at a central, regional and local levels were in place long before. These efforts can be placed into 4 categories: a) contextual factors, b) development of a new causal conceptual framework, c) political commitment at central, regional and local level, and civil society participation, and d) implementation of PPR programs, specifically PAN.

**Contextual Factors**

Various contextual factors characterizing the country in the 1990s and early 2000s played an essential role in setting the scene. First, sustained economic growth since the early 1990s paved the way for a long-term investment in health, particularly in reproductive, maternal and child health. Second, there was a significant reduction in under-5 mortality, from 80.4/1000 live births in 1990 to 38.5/1000 in 2000, which led to an emphasis on a heathy start in health, growth and development [143]. Third, the defeat of terrorism in the 1990s and return of democracy. Fourth, the consolidation of the democracy (and the corresponding discussion for a definition of a country agenda and the corresponding roadmap) in the early 2000s. There was an active advocacy role of society initiatives such as The National Agreement and The Roundtable Against Poverty, with the inclusion of the fight against poverty and against child stunting as top priorities, along with setting of specific goals followed by monitoring and accountability. In addition, the health reform efforts aimed at strengthening the health system to expand health services related to other sectors for providing other basic services across the country focusing on the poorest areas. This coupled with regional initiatives comprised of the implementation of health intervention packages focused on prevention rather than treatment to gather experience and evidence on preventive and multi-sectorial interventions to reduce child stunting. For example, the Good Start in Life Program (Programa Buen Inicio) led by UNICEF. Finally, the adoption of international commitments such as the Millennium Development Goals (MDGs) by Peru boosted the efforts to achieve measurable improvements in child health and nutrition.

*“An important aspect was the economic improvement in our country, which is not enough however; I mean, let´s remember that the improvements started to be consolidated since 2002, 2003. And this is a good starting point for two things. First, as contributors to other interventions, but also to give sustainability to the interventions as well”.* Participant 6. Former Technical Officer at UNICEF.

**Important Drivers**

According to participants, there were key drivers in the reduction of stunting in Peru, in addition to the contextual factors.

First, the development of a new causal conceptual framework for child stunting, based on the UNICEF conceptual framework for malnutrition was useful for policy action (Figure 4). The conceptual framework was built on experiences from different NGOs in diverse regions of the country implementing programs aimed at reducing childhood stunting through multi-sectorial and preventive interventions. This transformed the perspective of stunting to a problem that goes beyond a lack of food and poor feeding practices. Therefore, this emphasized the need for the participation of different sectors, a focus on prevention rather than treatment, and a call for the reduction of poverty and improvement of other social determinants of malnutrition.

*“When stunting reduction started, in 2005, 2006, there were experiences of different institutions, not only public experiences, which allowed to generate sufficient evidence to know what to do” -* Participant 2. Member of MIDIS.

An additional key driver of stunting reduction was effective and continued political leadership and commitment to reduce childhood stunting at national, regional and local levels, coupled with strong civil society participation. In 2005, an intense advocacy effort with the presidential candidates led to a formal commitment to include child stunting reduction as a top national priority. This leadership resulted in the implementation of the PPR programs to secure a budget line for the actions. A strong civil society advocacy ensured the positioning of stunting within the political agenda, as well as an effective monitoring process of the programs and policies subsequently implemented.

*“I believe that an important factor in stunting reduction is that it was included in the political agenda as a priority. That made the difference when compared with the approach to other public health problems (…). The fact we had a President that talked about child health and its link with the country development, was crucial”* – GFD. Participant 5. Coordinator, Nutrition and Child Development, MIDIS

*“(Stunting) was included in the political agenda as a multisectoral problem, needing commitment of regional and local governments… they assumed a lot of responsibility”-* Participant 3. Member, Technical Team of the Roundtable of Fight Against Poverty.

*“The National Agreement was set up, and priorities were established, such as children (…) The Roundtable Against Poverty was established, and building on it, within the context of the elections in 2005, political candidates signed commitments.”-* Participant 6. Former Technical Officer at UNICEF.

**Specific Policies and Programs: The Articulated Nutrition Program**

The PPR programs were led by the MoF. These programs systematized previous experiences and evidence from various institutions. Through this approach, the budget was allocated to different regions on the basis of results obtained. For maternal and child health, in 2007, the PAN and the PSMN were first to be introduced.

With implementation beginning in 2008, PAN utilized several interventions supported by scientific evidence as effective to reduce child stunting. Along with coordinating the implementation of out-of-health interventions such as water and sanitation, PAN was focused on the implementation of key interventions related to child health and nutrition, including the introduction of pneumococcal vaccine and the rotavirus vaccine as part of the national immunization scheme and the promotion of key healthy practices (exclusive breastfeeding, complementary feeding, promotion of handwashing). To strengthen healthy practices, the child growth and development monitoring component of the program was improved, increasing the number of child attendances at a health center and changing the emphasis on mere weight and height control to counseling on key practices to the mother (For more details on PAN, see Appendix 14).

The results of these programs were referred to as the accomplishment of specific progress achieved in terms of intervention coverage and impact indicators, including maternal, neonatal and child mortality, as well as stunting reduction at regional (departmental) level. They established goals and specific interventions and indicators to focus on, with particular emphasis on prevention and on the multi-causal nature of stunting.

*“To me, the design of the results-based budgeting programs was very important. We started with the Articulated Nutrition Program and the Strategic Maternal-Neonatal Program, in 2007. I felt that this was the beginning of a big change… because we knew where to reach, how to achieve our goals” -* Participant 1. Technical Officer, Ministry of Health.

PAN’s package of interventions were coordinated between the Ministry of Health, the MIDIS, the Presidency of the Council of Ministers, the Comprehensive Health Insurance (SIS), the Regional Governments and the Local Governments [144]. This program specifically established coordinated goals, interventions and indicators, with articulated strategies, while ensuring the efficient flow of processes and the avoidance of duplicate efforts and expenses.

A distinctive characteristic of the Articulated Nutrition Program is its coordination with programs implemented by other sectors. Thus, it was closely coordinated with JUNTOS, a conditional cash transfer program launched by the Peruvian government in 2005 and led by the Ministry of Development and Social Inclusion. To remain beneficiaries of JUNTOS and receive the cash transfer, families had to comply with certain conditionalities that included the use of maternal and child health preventive and curative services. According to participants, JUNTOS served as an effective incentive for families to use child health and education services.

The PAN was built on the experience gained by the implementation of the Good Start in Life Program (*Programa Buen Inicio*) led by UNICEF and by the experience of other NGOs, which demonstrated that working closely with local managers would lead to successful results. The implementation of the PAN was strengthened both financially and technically by the Budget Support Program for the Structured Nutritional Program (EUROPAN), to accelerate the reduction of childhood stunting and the fight against poverty [145].

*“We built on the experience of UNICEF and worked with diverse NGOs at local level. We linked this to EUROPAN experience… and we saw that working closely with the municipalities, with the local managers, we could make things happen”-* Participant 2. Member of the MIDIS.

Just as they stated when discussing the role of decentralization, participants insisted that technical support was key to make the PPR programs fully operational at the regional level. In regions where technical support was absent or insufficient, the programs failed.

Among other the facilitators for the implementation of the Articulated Nutrition Program, participants identified performance-based budget allocation/increased budget allocation for key interventions and health system strengthening as prominent ones.

Performance-based budget allocation meant that budget allocation to the regions was dependent on the compliance with the coverage and impact goals established by each region. This allowed investing in a more efficient process of personnel hiring, of equipment and supplies acquisition, and of better training of human resources. Additionally, investment in activities related to growth and development monitoring and to vaccines was prioritized with increased budget allocation. This was accompanied by technical support to the regions on how to spend the budget. In terms of health system strengthening, particular emphasis was put to increase substantially the number of health facilities at regional level, as well as to attract human resources for health and to retain them, particularly in rural areas.

There were some limitations in the implementation of the PAN and the PSMN. One of them is the quality of expenditure. Various activities were reported as part of the programs that in fact, did not belong to them. Another issue was that the budget was re-oriented to interventions that were part of the PPR, but neglected the budget line of other areas such as the health sector.

An additional challenge was the lack of health workforce to provide the growth and development monitoring, as well as logistic problems in putting interventions into place, such as the vaccines. These problems forced the regions to additionally invest in hiring human resources for health. There were also managerial barriers, mainly in terms of programming the distribution of supplies. Thus, one challenge at that level was to ensure a real commitment from decision makers to reach the established goals. One way to face this challenge was to implement performance-based incentives for managers.

Participants also acknowledged some drawbacks of JUNTOS that hampered a better coordination with PAN. For instance, the impact evaluations have been weak, and the results are not necessarily in line with the coverage information. Also, JUNTOS struggled to communicate appropriately to the families about the specific conditionalities and as a consequence sometimes families were removed from the program but did not understand the reasons of their exclusion. Additionally, it has not been clearly marketed as a program aimed at improving child health, but as a program targeted to mothers instead. Finally, it has not been clear for how long the families would remain as beneficiaries of JUNTOS.

Finally, the participants acknowledged the improvements in data generation, including the DHS and other administrative datasets, and how their increased use added evidence to strengthen advocacy, measured progress, and strengthened regional motivation. However, participants mentioned that some administrative datasets are limited to certain segments of the population, such as those attending the health facilities, but do not necessarily provide community level information.

**In Depth-Interviews**

Participants agreed that stunting reduction was more apparent following 2007. They concurred that the substantial reduction observed since 2007 was preceded by the experience gained through the independent implementation of different programs, in areas with the highest stunting prevalence. All participants stated that these experiences laid the foundation on which later multi-sectorial policies and programs led by the government were designed and implemented, which were based on a PPR approach, specifically the PAN. In line with the messages that emerged from the FGD, the interviews revealed the importance of contextual factors, they emphasized the role of the civil society advocacy as a separate and key driver, and also acknowledged the importance of the PAN. The interviewees also mentioned the importance of additional policies and programs.

**Contextual Factors**

Similar to the perceptions of the FGD participants, interviewees also pointed out the role of the same contextual factors that facilitated the implementation of policies and programs aimed at reducing childhood stunting. Namely: a) the sustained economic growth, b) the reduction of child and infant mortality, c) the terrorism defeat in 1991 and the subsequent consolidation of the democracy in the early 2000s that allowed an agenda of country reconstruction, d) the adoption of MDGs by the Peruvian government and specifically those related to maternal and child health and nutrition, and e) the support provided by international organizations like the International Development Bank to sustained governmental design and implementation of interventions that could be monitored in terms of coverage and impact indicators.

*“Before in Peru we were more concerned on avoiding deaths. That was in the 90s. Now we are more concerned on avoiding malnutrition and anemia”.* Participant 1. Technical Officer, Ministry of Economy and Finances.

*“We were leaving a profound country crisis (referring to terrorism), and also a break of democratic institutionality represented by the last period of Fujimori. Then there was a profound reflection by people with decision power, by the media. It was an important moment. It was a period when the need of having clear objectives, of building a country and making real changes for the people and particularly for the most vulnerable segments was discussed and defined. The need of privileging children and of building sustained policies across governments was clearly defined”.* Participant 5. Pediatrician. Former Technical Officer at UNICEF.

**Important Drivers**

According to interviewees, a main driver of stunting reduction was the change in paradigm of the determinants of stunting, from a feeding problem, to a multi-sectorial problem that needed to be addressed by different stakeholders and sectors. Albeit the health aspect continued to be considered, other factors were incorporated such as: a) childhood disease prevention (namely diarrheal diseases and ARI) that was reflected by emphasizing vaccine implementation, b) growth and development monitoring (once per month) focused on the promotion of key practices at home, such as, healthy raising practices and improved hygiene, c) the role played by political champions, d) the role of different ministries besides the Ministry of Health, e) the role of regional and local governments, f) the role of health personnel, and g) the role of families and mothers. There were various success stories led by different organizations that we highlight below. These stories were instrumental in providing evidence and advocacy at all governmental levels, and paved the way for designing and implementing effective national level interventions to reduce stunting. There were diverse stakeholders involved, in particular, the NGOs played a prominent role.

*“You stop looking at stunting as a feeding problem, and you start considering it as a multifactorial problem, although you don´t forget that infections have a role (in the origin of stunting). Then you start linking the different interventions”-* Participant 8. Technical Advisor, MoF

Political leadership and commitment at all levels was another key ingredient acknowledged by interviewees. In particular, the inclusion of stunting reduction as a top priority in the national and local agendas. The establishment of the National Agreement in 2001 was instrumental to reach the agreement between the diverse political parties, and to establish long-term policies to be implemented across different governments. One of these policies was the acknowledgement of the rights of children to fulfill their full potential and to achieve a healthy and productive life.

Participants also acknowledged that the signature of a commitment to reduce under-5 stunting by 5 percent points during a period of 5 years (Strategy 5x5x5) by all the presidential candidates in 2005 was monumental. Diverse NGOs lectured the candidates on the importance of reducing stunting for the country and on the available evidence, based on the success stories they experienced in Andean and Amazon areas, and to obtain their formal commitment. All participants insisted that the political commitment at regional and local levels was crucial, through transfer of political, financial and technical power to regional and local governments, a process that is, still unfinished and has faced many problems that go beyond the scope of this analysis. This transfer process was facilitated by the President of the Republic and the relationship established by the central government with the local actors. To achieve the engagement of regional and local governments with the implementation of interventions to reduce stunting, diverse strategies were implemented, including performance-based financial and non-financial incentives. One of them was the “Municipal Seal” (*Sello Municipal*), which was an award that acknowledged the achievement of a particular municipality.

*“I think that a central factor was the continued dialogue with the political parties, initially through the National Agreement. That occurred at about 2004, 2005. Besides, one of the priority themes within the National Agreement was to work on the rights of children”* Participant 6. Member, Roundtable Against Poverty.

*“There were specific goals (for municipalities to achieve), such as ID for all children and citizens. Contests involving local governments were conducted, and the winner municipalities received the Municipal Seal, along with a President photograph and a plaque”.* Participant 2. Independent Consultant. Former Member, MIDIS.

According to interviewees, civil society advocacy was important and aligned the population needs with the budget. That is, they identified the priorities and allocated the budget according to those needs. There was an intense advocacy at the MoF. Two organizational components critical to channel the civil society advocacy role were the Roundtable Against Poverty and the Child Malnutrition Initiative (CMI). Their members included NGO affiliates whose experiences to reduce stunting were considered successful and thus useful to extend the evidence-based interventions at national level. The Roundtable Against Poverty was previously mentioned. As for the CMI, it was a collective organization promoted initially in 2006 by ADRA, CARE, Caritas, and PRISMA. USAID was incorporated shortly afterwards. This included a technical group and a communication group. In 2006, the Roundtable Against Poverty, FAO, PAHO, WFP, UNICEF and UNFPA were incorporated, and The World Bank was invited. Generaciones Futuras and the Instituto de Investigaciones Nutricionales were incorporated in 2007 and 2008, respectively. Acción Contra el Hambre (2010), Plan Internacional (2010), World Vision (2011) and Management Sciences for Health (2011) were later additions. Currently, the CMI has 17 organizations as active members [146].

The CMI was instrumental in achieving the commitment of political candidates in the fight against stunting in the general elections of 2006 and 2011. In addition, it provided technical support to the central and regional governments in the design and implementation of interventions to reduce stunting. The CMI collaborated with the MoF to establish the PPR programs to improve budget allocation at regional and local levels. Its advocacy was decisive for the preparation of annual reports on stunting and its determinants at regional level by the National Institute of Statistics and Computing (INEI). It contributes to the monitoring of the progress achieved in stunting reduction at national and regional levels. Finally, it has provided support to the national and regional governments to contact with national and international organizations [146].

*“The civil society advocacy on childhood issue was very important… they talked directly to the Minister of Economy and Finances … Then we worked together all the process (of policies and programs implementation) with all civil society members”.* Participant 2. Independent Consultant. Former Member, MIDIS.

The implementation of a cross-cutting PPR program, namely the PAN, was mentioned by all participants as the key factor in the accelerated stunting reduction in Peru from 2008 onward, and we elaborate on it in a later section.

**Pre-2007 Landmark Policies and Programs**

While acknowledging the key role of PPR programs and specifically of the PAN in the reduction of childhood stunting, interviewees pointed out that there were pre-2007 landmark programs that laid out the foundation for later implementation of cross-cutting multidisciplinary and prevention-centered policies and programs to reduce stunting. We elaborate on them following a chronological sequence in the forthcoming sections.

First, the Social Development Cooperation Fund-*(Fondo de Cooperación para el Desarrollo Social, FONCODES)*, was a key factor in the design and implementation of antipoverty strategies during the 1990s, it started in 1991. The main objectives included generation of employment, poverty alleviation, and improving the accessibility of social services through direct investment in infrastructure construction in rural areas and shanty towns with high poverty levels [147]. After a decade of implementation, FONCODES incorporated a training component and participation at local level, keeping the executive core team, and further focusing on rural areas. It was part of the Ministry of Woman and Social Development. FONCODES strategies included the generation of sustainable economic opportunities for extremely poor rural families, and facilitating the coordination between private actors and the community. FONCODES was therefore conceived as a contributor to the reduction of social exclusion and as a facilitator of the incorporation of poor families to the formal market. FONCODES also led to an increase in the accessibility to basic services such as water and sanitation which may have also contributed to stunting reduction.

Second, the Good Start in Life Program (*Buen Inicio)*, led by UNICEF and was implemented in 2000 in 4 departments such as: Andes (Cusco, Cajamarca, Apurimac) and the Amazon (Loreto). This program required active and coordinated participation of diverse NGOs, health personnel, diverse ministries and communities. *Buen Inicio* included two main components: a) management processes, including strengthening of institutional and individual capacities, and strengthening of financial, human and organizational resources, which crossed all operational levels and programmatic areas, and built on a participatory approach and b) a package of interventions focused on young children that included a preventative and promotional approach in nutrition, hygiene, health, and psychological stimulus. This intervention included five main aspects: research, training, social communication, information and advocacy, growth and development monitoring, and community surveillance. The actions encompassed the continuum of care, including antenatal care, nutrition of pregnant women, infants and children, exclusive breastfeeding, iron and vitamin A supplementation, growth and developing monitoring, child stimulus, iodine-supplemented salt consumption, and personal and familial hygiene.

Third, the Program of Support to Health Sector Reform (PARSALUD 1) implemented since 2003 by the Ministry of Health, which aimed at increasing the coverage of essential interventions with emphasis on mothers and newborns of poor areas. PARSALUD established this by strengthening infrastructure, improving the supply of equipment and drugs, by training personnel in health management and clinical capabilities, and by addressing cultural adaptation of health services to increase the demand for health services [148].

Fourth, the Sustainable Networks for Food Security Program-REDESA, (*Programa Redes Sostenibles para la Seguridad Alimentaria, REDESA*) was implemented by CARE from 2002 to 2006, in the rural communities of Ancash, Apurímac, Ayacucho, Cajamarca, Huancavelica and Puno. The main goal of the program was the reduction of stunting in children younger than 3 years old. Of note, it did not include food distribution as one of its actions. The program intervention relied “on a combination of sustainable income generation and improvement of health conditions, with a cross-cutting focus on building networks of strategic alliances and partnerships with local, public, private sector and civil society organizations” [128].

**PPR Programs: The PAN**

It was introduced and led by the MoF in 2007 and scaled up in 2009. It is part of the PPR approach, which links budget allocation to measurable products and population-relevant results. This required a strong institutional commitment, a clear identification of those responsible for implementing the PPR interventions, clear accountability mechanisms on public spending, as well as a functional information system for monitoring different aspects of the program [10]. The PPR strategy was introduced in Peru in 2007 and continues to date. It was adopted by Peru after a series of analyses showed that the efforts to improve human development indicators including childhood stunting had not led to the expected results. At the same time, various experiences, particularly in certain departments of the Andes and the Amazon, showed that it is possible to reduce poverty, to increase the coverage of different interventions and to improve social indicators such as literacy and child nutrition. Civil society members, communities and organizations participated in analytical efforts and in the pressuring different government levels to be accountable when implementing and evaluating public interventions. Likewise, the need to emphasize the quality of services was acknowledged. Thus, the PPR strategy was implemented with the specific objective to ensure that the population but particularly the most vulnerable populations receive the required public services, to achieve a better quality of life.

*“In 2007 (PPR) started to be designed, during 2008 the design was refined and implementation started. By 2009 there was already a budget line and spending (in interventions implementation) started, such as vaccines. I think that we could see the results by 2010…The program is in line with an evidence-based policy. It was a special, robust program”.* Participant 3. Former Member, Technical Team of the MoF.

According to participants, although a systematic and rigorous evaluation on its impact has not been conducted, PPR implementation has been successful, particularly in giving areas of the country that were able to achieve substantial improvements of indicators like childhood stunting. They concurred that achievements of PPR implementation include the definition of specific goals, the identification of priority interventions and indicators, and emphasizing prevention and multi-causality of the stunting problem. It also allowed the coordination between different sectors such as the Presidency of the Council of Ministers, the Ministry of Health, the Ministry of Education, the Ministry of the Woman and Social Development, the MIDIS, the Comprehensive Health Insurance (SIS), the Regional Governments and the Local Governments. It also endorsed the coordination between programs such as the CCT program JUNTOS, water and sanitation programs, education programs, and health programs such as the Comprehensive Health Insurance System (SIS), which had started earlier.

*“The results-based budgeting programs were not all successful, but one in particular was successful and was sustained, it was the Articulated Nutrition Program. This, yes, it played an important role, it was key, very important (in the fight against stunting)”*. Participant 7. Technical Officer, PAHO.

The program articulated the implementation of several interventions supported by scientific evidence as effective to reduce child stunting. In addition, the PAN was focused on the implementation of key interventions related to child health and nutrition, including the introduction of pneumococcal vaccine and the rotavirus vaccine as part of the national immunization scheme and the promotion of key healthy practices (exclusive breastfeeding, complementary feeding, promotion of handwashing). To strengthen the healthy practices, the growth and development monitoring component was improved, therefore increasing the number of child attendances and shifting the focus from weight and height control to counseling on key practices to the mother.

The PPR monitoring, specifically monitoring of PAN also required a more thorough monitoring, and thus it was agreed that the DHS had to report on its progress twice a year, an initiative that complemented the previous decision to conduct annually the DHS from 2004 onward, thus strengthening monitoring at national and departmental level. Likewise, PPR led to implementation of additional information systems to monitor health services provision, availability of equipment, supplies, human resources, and other administrative information not provided by the DHS.

Participants identified three additional prominent facilitating factors for the implementation of the PAN prior to 2007. Firstly, performance-based budget allocation was dependent on the compliance with the coverage and impact goals established by each region. This allowed a more efficient process of personnel hiring, of equipment and supplies acquisition, and of training of human resources. Secondly, increased budget allocation for key interventions in all regions, with the highest priority given to activities related to growth and development monitoring, and to vaccines, was accompanied by technical support to the regions on how to spend the budget. Thirdly, health system strengthening, with a particular emphasis on increasing the number of health facilities at the regional level, and to attract HRH and to retain them, in rural areas.

Some drawbacks highlighted by participants in the implementation of the PAN was the inability of healthcare force to provide growth and development monitoring. In addition, there were logistic issues making intervention products and vaccines unavailable. These problems forced the regions to further invest in HRH. There were also managerial challenges, such as organizing the distribution of supplies, and a lack of decision power of nurses on logistics. One way to face this challenge was to implement performance-based incentives for managers. There was also poor compliance with technical guidelines, although it was not a generalized problem, and it was probably due to limited training of health personnel at the local level.

Of note, along with the PAN and the PSMN, other PPR programs focused on infants and children were also implemented. These programs include the identity program to provide identification for all citizens and in particular children, the education program to monitor learning achievements, and the road program, an expanded initiative aimed at improving access to basic services and to market opportunities through road infrastructure construction.

# **Supplementary Appendix References**

1 World Bank. World Bank Indicators. 2018.https://data.worldbank.org/indicator

2 JMP. Joint Monitoring Programme for Water, Supply, Sanitation and Hygiene: Peru. 2017.https://washdata.org/data/household#!/

3 Black RE, Allen LH, Bhutta ZA, *et al.* Maternal and Child Undernutrition: Maternal and child undernutrition: global and regional exposures and health consequences. *Lancet* 2008;**371**:243–60. doi:10.1016/S0140

4 Stifel D, Alderman H. The ‘Glass of Milk’ subsidy program and malnutrition in Peru. *World Bank Econ Rev* 2006;**20**:421–48. doi:10.1093/wber/lhl002

5 Gross R, Lechtig A, Lopez de Romana D. Baseline evaluation of nutritional status and government feeding programs in Chiclayo, Peru. *Food Nutr Bull* 2006;**27**:S115–21.

6 Acosta AM. Analysing success in the fight against malnutrition in Peru. IDS Work. Pap. - Inst. Dev. Stud. 2011.

7 World Bank. *Peru: Country Program Evaluation for the world Bank Group, 2003-09*. Washington, D.C.: : The International Bank for Reconstruction and Development 2011.

8 Galasso E, Wagstaff A, Naudeau S, *et al.* The Economic Costs of Stunting and How to Reduce Them. 2017. http://pubdocs.worldbank.org/en/536661487971403516/PRN05-March2017-Economic-Costs-of-Stunting.pdf (accessed 14 Dec 2017).

9 Krishna A, Oh J, Lee JK, *et al.* Short-term and long-term associations between household wealth and physical growth: a cross-comparative analysis of children from four low- and middle-income countries. *Glob Heal Action* 2015;**8**:26523.

10 Marini A, Rokx C, Gallagher P. *Standing Tall Peru’s Success in Overcoming its Stunting Crisis*. Washington, DC: : International Bank for Reconstruction and Development / The World Bank Group 2017.

11 Urke HB, Mittelmark MB, Valdivia M. Trends in stunting and overweight in Peruvian pre-schoolers from 1991 to 2011: Findings from the Demographic and Health Surveys. *Public Health Nutr* 2013;**17**:2407–18. doi:10.1017/S1368980014000275

12 Acosta AM, Haddad L. The politics of success in the fight against malnutrition in Peru. *Food Policy* 2014;**44**:26–35.

13 Escobal J, Benites S. Algunos impactos del programa JUNTOS en el bienestar de los niños: Evidencia basada en el estudio Niños del Milenio. *Boletín 7 Políticas Públicas del Estud Niños del Milen* 2012.

14 Huicho L, Huayanay-Espinoza CA, Herrera-Perez E, *et al.* Factors behind the success story of under-five stunting in Peru: a district ecological multilevel analysis. *BMC Pediatr* 2017;**17**:29. doi:10.1186/s12887-017-0790-3

15 Marini, Alessandra, Arias O. The three factors to halving childhood stunting in Peru over just a decade | Investing in Health. World Bank. 2016.

16 Sanchez A, Melendez G, Behrman J. The Impact of the Juntos Conditional Cash Transfer Programme in Peru on Nutritional and Cognitive Outcomes: Does the Age of Exposure Matter? Oxford, UK: 2016.

17 Sánchez A, Jaramillo M. Impacto del programa Juntos sobre nutrición temprana. *Work Pap Ser* Published Online First: 2012.http://www2.juntos.gob.pe/storage/ckeditor/hjMwhziLC2TXCSy15luSbHINWKTuwO.pdf

18 Hernandez-Vasquez A, Tapia-Lopez E. [Chronic Malnutrition among Children under Five in Peru: A Spatial Analysis of Nutritional Data, 2010-2016]. *Rev Esp Salud Publica* 2017;:19.

19 Huicho L, Huayanay-Espinoza CA, Herrera-Perez E, *et al.* Examining national and district-level trends in neonatal health in Peru through an equity lens: a success story driven by political will and societal advocacy. *BMC Public Health* 2016;**16**. doi:10.1186/s12889-016-3405-2

20 Yamada Fukusaki G, Castro JF, Yamada Fukusaki G, *et al.* Poverty, inequality, and social policies in Peru : as poor as it gets. *Univ del Pacífico* 2012.

21 Lutter C, Chaparro C. *Malnutrition in infants and young children in Latin America and the Caribbean : Achieving the and Millennium Development Goals*. 2008.

22 Larrea C, Freire W. Social inequality and child malnutrition in four Andean countries. *Rev Panam Salud Publica/Pan Am J Public Heal* 2002;**11**:356–64.

23 Anticona C, San Sebastian M. Anemia and malnutrition in indigenous children and adolescents of the Peruvian Amazon in a context of lead exposure: a cross-sectional study. *Glob Health Action* 2014;**7**:22888.

24 Nolan LB. Rural-urban child height for age trajectories and their heterogeneous determinants in four developing countries. *Popul Res Policy Rev* 2016;**35**:599–629.

25 Aramburú La Torre AM. Diversidad alimentaria y su asociación con el retraso del crecimiento en niños de 6-23 meses. Perú, 2008-2010. 2014;:viii,62-viii,62.http://bvssp.icict.fiocruz.br/lildbi/docsonline/get.php?id=3890

26 Loret De Mola C, Quispe R, Valle GA, *et al.* Nutritional Transition in Children under Five Years and Women of Reproductive Age: A 15-Years Trend Analysis in Peru. *PLoS One* 2014;**9**:e92550. doi:10.1371/journal.pone.0092550

27 UNICEF. *Improving child nutrition: The achievable imperative for global progress.* 2013. doi:978-92-806-4686-3

28 Pawson IG, Huicho L. Persistence of growth stunting in a Peruvian high altitude community, 1964-1999. *Am J Hum Biol* 2010;**22**:367–74.

29 De Meer K, Bergman R, Kusner JS, *et al.* Differences in physical growth of Aymara and Quechua children living at high altitude in Peru. *Am J Phys Anthropol* 1993;**90**:59–75.

30 Sobrino M, Gutiérrez C, Cunha AJ, *et al.* Child malnutrition in children under 5 years of age in Peru: Trends and determinants. *Rev Panam Salud Publica/Pan Am J Public Heal* 2014;**35**:104–12.

31 Gragnolati M, Marini A. Nonlinear effects of altitude on child growth in Peru a multilevel analysis. *Policy Res Work Pap 3823* 2006;:1–21.

32 Yosef S, Goulden J. Commitments and accountability: Peru’s unique nutrition journey. In: Gillespie S, Hodge J, Yosef S, *et al.*, eds. *Nourishing millions: Stories of Change in Nutrition*. Washington, D.C.: : International Food Policy Research Institute (IFPRI) 2016. 125–32. doi:10.2499/9780896295889_14

33 Levinson FJ, Balarajan Y. Adressing Malnutrition Multisectorally: What have we learned from recent international experience? New York: 2013.

34 Perova E, Vakis R. 5 Years in Juntos: New Evidence on the Program’s Short and Long-Term Impacts. *Economía* 2012;**35**:53–82.

35 Gajate-Garrido G. Excluding the Rural Population The Impact of Public Expenditure on Child Malnutrition in Peru. *Policy Res Work Pap 6666* 2013.

36 Huicho L, Davila M, Gonzales F, *et al.* Implementation of the Integrated Management of Childhood Illness strategy in Peru and its association with health indicators: an ecological analysis. *Health Policy Plan* 2005;**20**:i32–41.

37 Forero-Ramirez N, Gamboa LF, Bedi A, *et al.* Child malnutrition and prenatal care: evidence from three Latin American countries. *Rev Panam Salud Publica/Pan Am J Public Heal* 2014;**35**:163–71.

38 Robert RC, Gittelsohn J, Creed-Kanashiro HM, *et al.* Implementation examined in a health center-delivered, educational intervention that improved infant growth in Trujillo, Peru: successes and challenges. *Health Educ Res* 2006;**22**:318–31. doi:10.1093/her/cyl078

39 Lutter CK, Iannotti L, Creed-Kanashiro H, *et al.* Key principles to improve programmes and interventions in complementary feeding. *Matern Child Nutr* 2013;**9 Suppl 2**:101–15.

40 Lechtig A, Cornale G, Ugaz ME, *et al.* Nutritional impact of the good start in life program (Buen Inicio) in Peru: decreased prevalence of stunting, anemia and vitamin A deficiency in children less than three years of age, from the poorest indigenous populations in the Andean highlands and the. *Sight Life Mag* 2009;**2**:16–9.http://ovidsp.ovid.com/ovidweb.cgi?T=JS&CSC=Y&NEWS=N&PAGE=fulltext&D=caba6&AN=20093251211

41 Santos AP, Oliart-Guzman H, Branco LFCC, *et al.* Prevalence of nutritional deficiencies and morbidity by infectious diseases in children 0-5 years of age in Inapari in the Peruvian Amazon. *Rev Patol Trop* 2016;**45**:305–21.

42 Humphries D, Dearden KA, Schott W, *et al.* Children with access to improved sanitation but not improved water are at lower risk of stunting compared to children without access: a cohort study in Ethiopia, India, Peru, and Vietnam. *Am J Trop Med Hyg* 2014;**1)**:180.

43 Hartinger S, Lanata C, Hattendorf J, *et al.* A community randomized controlled trial of an integrated home-based intervention improving household-air pollution, drinking water quality and hygiene in rural peru. Am. J. Trop. Med. Hyg. 2012;**87**:285.

44 Gyorkos TW, Maheu-Giroux M, Casap, *et al.* Stunting and helminth infection in early preschool-age children in a resource-poor community in the Amazon lowlands of Peru. *Trans R Soc Trop Med Hyg* 2011;**105**:204–8. doi:10.1016/j.trstmh.2010.12.003

45 Casapía M, Joseph SA, Núñez C, *et al.* Parasite and maternal risk factors for malnutrition in preschool-age children in Belen, Peru using the new WHO child growth standards. *Br J Nutr* 2007;**98**:1259–66. doi:10.1017/S0007114507795272

46 Georgiadis A, Benny L, Duc LT, *et al.* Growth recovery and faltering through early adolescence in low- and middle-income countries: Determinants and implications for cognitive development. *Soc Sci Med* 2017;**179**:81–90.http://libaccess.mcmaster.ca/login?url=http://ovidsp.ovid.com/ovidweb.cgi?T=JS&CSC=Y&NEWS=N&PAGE=fulltext&D=prem&AN=28260638

47 Cabada MM, Goodrich MR, Graham B, *et al.* Prevalence of intestinal helminths, anemia, and malnutrition in Paucartambo, Peru. *Rev Panam Salud Publica/Pan Am J Public Heal* 2015;**37**:69–75.

48 Huicho L, Vila MD, Campos M, *et al.* Scaling up Integrated Management of Childhood Illness to the national level: achievements and challenges in Peru. *Health Policy Plan* 2005;**20**:14–24. doi:10.1093/heapol/czi002

49 Valdivia M. Poverty, health infrastructure and the nutrition of Peruvian children. *Econ Hum Biol* 2004;**2**:489–510. doi:10.1016/j.ehb.2004.10.008

50 Shin H. Child health in Peru: Importance of regional variation and community effects on children’s height and weight. *J Health Soc Behav* 2007;**48**:418–33.

51 Urke HB, Bull T, Mittelmark MB. Socioeconomic status and chronic child malnutrition: wealth and maternal education matter more in the Peruvian Andes than nationally. *Nutr Res* 2011;**31**:741–7.

52 Alderman H, Hentschel J, Sabates R. With the help of one’s neighbors: externalities in the production of nutrition in Peru. *Soc Sci Med* 2003;**56**:2019–31.

53 Saito M, Goel-Apaza S, Espetia S, *et al.* Multiple norovirus infections in a birth cohort in a peruvian periurban community. *Clin Infect Dis* 2014;**58**:483–91.

54 Iannotti LL, Zavaleta N, Leon Z, *et al.* Growth and body composition of Peruvian infants in a peri urban setting. *Food Nutr Bull* 2009;**30**:245–53.

55 Schott WB, Crookston BT, Lundeen EA, *et al.* Periods of child growth up to age 8 years in Ethiopia, India, Peru and Vietnam: Key distal household and community factors. *Soc Sci Med* 2013;**97**:278–87.

56 Mariños-Anticona C, Chaña-Toledo R, Medina-Osis J, *et al.* Determinantes sociales de la desnutrición crónica infantil en el Perú. *Rev peru epidemiol* 2014;**18**:1–7.

57 PAHO. Peru. *Heal Am* 2012.

58 Ruel MT, Menon P. Child feeding practices are associated with child nutritional status in Latin America: innovative uses of the Demographic and Health Surveys. *J Nutr* 2002;**132**:1180–7.

59 Favara M. ‘United we stand divided we fall’: maternal social participation and children’s nutritional status in Peru. Policy Res. Work. Pap. - World Bank; 2012. (6264)38 pp. 78 ref. 2012.

60 De Silva MJ, Harpham T. Maternal social capital and child nutritional status in four developing countries. *Heal Place* 2007;**13**:341–55.

61 Bennett I, Schott W, Krutikova S, *et al.* Maternal Mental Health and Child Growth and Developmental Outcomes in Four Developing Countries. 2014. doi:10.1136/jech.2005.039180

62 Harpham T, Huttly S, De Silva MJ, *et al.* Maternal mental health and child nutritional status in four developing countries. *J Epidemiol Community Health* 2005;**59**:1060–4.

63 Penny ME, Creed-Kanashiro HM, Robert RC, *et al.* Effectiveness of an educational intervention delivered through the health services to improve nutrition in young children: a cluster-randomised controlled trial. *Lancet* 2005;**365**:1863–72. doi:10.1016/S0140-6736(05)66426-4

64 Waters HR, Penny ME, Creed-Kanashiro HM, *et al.* The cost-effectiveness of a child nutrition education programme in Peru. Published Online First: 2006. doi:10.1093/heapol/czl010

65 Hamad R, Fernald LCH, Karlan DS. Health education for microcredit clients in Peru: a randomized controlled trial. *BMC Public Health* 2011;**11**.

66 Marin CM, Segura JL, Bern C, *et al.* Seasonal change in nutritional status among young children in an urban shanty town in Peru. *Trans R Soc Trop Med Hyg* 1996;**90**:442–5.

67 Moseson H, Hamad R, Fernald L. Microcredit participation and child health: results from a cross-sectional study in Peru. *J Epidemiol Community Heal* 2014;**68**:1175–81.

68 Roche ML, Creed-Kanashiro HM, Tuesta I, *et al.* Traditional Food System Provides Dietary Quality for the Awajún in the Peruvian Amazon. *Ecol Food Nutr* 2007;**46**:377–99.

69 Flores-Bendezu J, Calderon J, Rojas B, *et al.* Chronic malnutrition and anemia in children under 5 years of indigenous households of Peru - analysis of Demographic and Health Survey 2013. *An la Fac Med* 2015;**76**:135–40.

70 Dornan P, Woodhead M. How Inequalities Develop through Childhood. 2015.

71 Humphries DL, Dearden KA, Crookston BT, *et al.* Household food group expenditure patterns are associated with child anthropometry at ages 5, 8 and 12 years in Ethiopia, India, Peru and Vietnam. *Econ Hum Biol* 2017;**26**:30–41.

72 Humphries DL, Dearden KA, Crookston BT, *et al.* Cross-sectional and longitudinal associations between household food security and child anthropometry at ages 5 and 8 years in Ethiopia, India, Peru, and Vietnam. *J Nutr* 2015;**145**:1924–33.

73 Checkley W, Epstein LD, Gilman RH, *et al.* Effects of acute diarrhea on linear growth in Peruvian children. *Am J Epidemiol* 2003;**157**:166–75.

74 Lee GO, Paredes Olortegui M, Salmón-Mulanovich G, *et al.* Early child health in an informal settlement in the Peruvian Amazon. *BMC Int Health Hum Rights* 2016;**16**:1–9. doi:10.1186/s12914-016-0099-6

75 Lee G, Yori P, Olortegui MP, *et al.* Comparative effects of vivax malaria, fever and diarrhoea on child growth. *Int J Epidemiol* 2012;**41**:531–9.

76 Acosta GJ, Vigo NI, Durand D, *et al.* Diarrheagenic Escherichia coli: prevalence and pathotype distribution in children from Peruvian rural communities. *Am J Trop Med Hyg* 2016;**95**:574–9.

77 Jaganath D, Saito M, Gilman RH, *et al.* First Detected Helicobacter pylori Infection in Infancy Modifies the Association Between Diarrheal Disease and Childhood Growth in Peru. *Helicobacter* 2014;**19**:272–9.

78 Passaro DJ, Taylor DN, Gilman RH, *et al.* Growth slowing after acute Helicobacter pylori infection is age-dependent. *J Pediatr Gastroenterol Nutr* 2002;**35**:522–6.

79 Joseph SA, Casapia M, Montresor A, *et al.* The Effect of Deworming on Growth in One-Year-Old Children Living in a Soil-Transmitted Helminth-Endemic Area of Peru: A Randomized Controlled Trial.[Erratum appears in PLoS Negl Trop Dis. 2015 Dec;9(12):e0004288; PMID: 26636967]. *PLoS Neglected Trop Dis [electronic Resour* 2015;**9**:e0004020.

80 Hollm-Delgado M, Gilman RH, Bern C, *et al.* Lack of an adverse effect of Giardia intestinalis infection on the health of Peruvian children. *Am J Epidemiol* 2008;**168**:647–55.

81 Mofid LS, Casapía M, Aguilar E, *et al.* A Double-Blind Randomized Controlled Trial of Maternal Postpartum Deworming to Improve Infant Weight Gain in the Peruvian Amazon. *PLoS Negl Trop Dis* 2017;**11**. doi:10.1371/journal.pntd.0005098

82 Creed-Kanashiro HM, Carrasco M, Abad M, *et al.* Promotion of traditional foods to improve the nutrition and health of the Awajun of the Cenepa River in Peru. Rome: 2013.

83 Iannotti L, Zavaleta N, León Z, *et al.* Maternal zinc supplementation and growth in Peruvian infants. Am. J. Clin. Nutr. 2008;**88**:154–60.

84 Penny M, Marin R, Duran A, *et al.* Randomized controlled trial of the effect of daily supplementation with zinc or multiple micronutrients on the morbidity, growth, and micronutrient status of young Peruvian children. Am. J. Clin. Nutr. 2004;**79**:457–65.

85 Arsenault JE, Havel PJ, de Romaña DL, *et al.* Longitudinal measures of circulating leptin and ghrelin concentrations are associated with the growth of young Peruvian children but are not affected by zinc supplementation. *Am J Clin Nutr* 2007;**86**:1111–9.

86 Brown KH, de Romaña DL, Arsenault JE, *et al.* Comparison of the effects of zinc delivered in a fortified food or a liquid supplement on the growth, morbidity, and plasma zinc concentrations of young Peruvian children. *Am J Clin Nutr* 2007;**85**:538–47.

87 Lopez de Romana G, Cusirramos S, Lopez de Romana D, *et al.* Efficacy of multiple micronutrient supplementation for improving anemia, micronutrient status, growth, and morbidity of Peruvian infants. *J Nutr* 2005;**135**:646S-652S.

88 López de Romaña D, Verona S, Aquino Vivanco O, *et al.* Protective effect of multimicronutrient supplementation against anemia among children, women, and adolescent girls in lower-income areas of Chiclayo, Peru. *Food Nutr Bull* 2006;**27**:S143–50.

89 Smuts CM, Lombard CJ, Spinnler Benadé AJ, *et al.* Efficacy of a foodlet-based multiple micronutrient supplement for preventing growth faltering, anemia, and micronutrient deficiency of infants: The four country IRIS trial pooled data analysis. *J Nutr* 2005;**135**:631S-638S.

90 De Romania GL. Experience with complementary feeding in the FONCODES Project. *Food Nutr Bull* 2000;**21**:43–8.

91 Duggan C, Penny ME, Hibberd P, *et al.* Oligofructose-supplemented infant cereal: 2 randomized, blinded, community-based trials in Peruvian infants. *Am J Clin Nutr* 2003;**77**:937–42.

92 Lechtig A, Cornale G, Ugaz ME, *et al.* Decreasing stunting, anemia, and vitamin A deficiency in Peru: results of the Good Start in Life Program. *Food Nutr Bull* 2009;**30**:37–48.

93 Huicho L, Segura ER, Huayanay-Espinoza CA, *et al.* Child health and nutrition in Peru within an antipoverty political agenda: a Countdown to 2015 country case study. *Lancet Glob Heal* 2016;**4**:e414–26. doi:10.1016/S2214-109X(16)00085-1

94 Rojas D C, Flores M R, Céspedes K R. Resultados de un programa de seguridad alimentaria en la reducción de la desnutrición crónica y sus factores causales en niños peruanos. *Rev Peru Med Exp Salud Publica* 2007;**24**:111–20.

95 Marquis GS, Habicht JP, Lanata CF, *et al.* Association of breastfeeding and stunting in Peruvian toddlers: An example of reverse causality. *Int J Epidemiol* 1997;**26**:349–56.

96 Marquis GS, Habicht JP. Breastfeeding and stunting among toddlers in Peru. *Adv Exp Med Biol* 2000;**478**:163–72.

97 Marquis GS, Habicht J, Lanata CF, *et al.* Breast milk or animal-product foods improve linear growth of Peruvian toddlers consuming marginal diets. *Am J Clin Nutr* 1997;**66**:1102–9.

98 Krishna A, Fink G, Berkman LF, *et al.* Short- and long-run associations between birth weight and children’s height. *Econ Hum Biol* 2016;**21**:156–66.

99 Budge S. Maternal Perceptions of Child Weight and Height and the Double Burden of Malnutrition : MSc Project Report height and the double burden of malnutrition : Young Lives , Peru Supervisors : Paula Sheppard & Lenka Benova. 2015.

100 Joseph SA, Casapia M, Blouin B, *et al.* Risk factors associated with malnutrition in one-year-old children living in the Peruvian Amazon. *PLoS Negl Trop Dis* 2014;**8**.

101 Roche ML, Creed-Kanashiro HM, Tuesta I, *et al.* Infant and young child feeding in the Peruvian Amazon: the need to promote exclusive breastfeeding and nutrient-dense traditional complementary foods. *Matern Child Nutr* 2011;**7**:284–94.

102 Dornan P, Pells K. From Infancy to Adolescence: Growing Up in Poverty Preliminary Findings from Round 4 of Young Lives. 2014.

103 Sobrino M, Gutierrez C, Alarcon J, *et al.* Birth interval and stunting in children under five years of age in Peru (1996-2014). *Child Care, Heal Dev* 2017;**43**:97–103.http://libaccess.mcmaster.ca/login?url=http://ovidsp.ovid.com/ovidweb.cgi?T=JS&CSC=Y&NEWS=N&PAGE=fulltext&D=prem&AN=27804155

104 Victora C G, Huttly, S R, Fuchs, S C, *et al.* The role of conceptual frameworks in epidemiological analysis: a hierarchical approach. *Int J Epidemiol* 1997;**26**:224–7.

105 Jann B. The Blinder–Oaxaca decomposition for linear regression models. *Stata J* 2008;**8**:453–79.

106 Headey D, Hoddinott J, Park S. Accounting for nutritional changes in six success stories: A regression- decomposition approach. *Glob Food Sec* 2017;**13**:12–20. doi:10.1016/j.gfs.2017.02.003

107 Headey D, Hoddinott J, Park S. Drivers of nutritional change in four South Asian countries: A dynamic observational analysis. *Matern Child Nutr* 2016;**12**:210–8. doi:10.1111/mcn.12274

108 Woodruff BA, Wirth JP, Bailes A, *et al.* Determinants of stunting reduction in Ethiopia 2000 – 2011. *Matern Child Nutr* 2017;**13**. doi:10.1111/mcn.12307

109 Headey DD, Hoddinott J. Understanding the Rapid Reduction of Undernutrition in Nepal. *PLoS One* 2015;**10**:e0145738. doi:10.1371/journal.pone.0145738

110 Alderman H, Headey D. The timing of growth faltering has important implications for observational analyses of the underlying determinants of nutrition outcomes. *PLoS One* 2018;**13**:e0195904. doi:10.1371/journal.pone.0195904

111 PAHO/WHO, Partnership for Maternal Newborn & Child Health, World Bank, *et al.* Success Factors for Women’s and Children’s Health: Peru. Geneva: 2015.

112 World Bank. Peru. CDF Profiles. http://siteresources.worldbank.org/CDFINTRANET/Resources/Peru.pdf

113 Jones N, Vargas R, Villar E. Conditional Cash Transfers In Peru: Tackling The Multi-Dimensionality Of Poverty And Vulnerability. Published Online First: 2006.https://www.unicef.org/files/Conditional_Cash_Transfers_In_Peru_-_Tackling_The_Multi-Dimensionality_Of_Poverty_And_Vulnerability.pdf (accessed 30 May 2018).

114 London School of Hygiene & Tropical Medicine. Peru: Countdown to 2015 case study. Futur. Learn. https://www.futurelearn.com/courses/women-children-health/0/steps/8974 (accessed 22 Nov 2017).

115 Cotlear D, Vermeersch C. Peruvian lessons for the transition from MDGs to SDGs. *Lancet Glob Heal* 2016;**4**:e353–4. doi:10.1016/S2214-109X(16)30069-9

116 Ministerio de Desarrollo e Inclusion Social. ESTRATEGIA NACIONAL DE DESARROLLO E INCLUSIÓN SOCIAL: INCLUIR PARA CRECER. Lima, Peru: www.midis.gob.pe

117 Vermeersch C, Medici AC, Narvaez R. Sustainable Development Country Summary Report for Peru. 2014.

118 Stifel D, Alderman H. The ‘Glass of Milk’ subsidy program and malnutrition in Peru. *World Bank Econ Rev* 2006;**20**:421–48. doi:10.1093/wber/lhl002

119 USAID. USAID’S PARTNERSHIP WITH PERU ADVANCES FAMILY PLANNING. 2016.

120 Schuler S. Gender and Community Participation in Reproductive Health Projects: Contrasting Models from Peru and Ghana. *Reprod Health Matters* 1999;**7**:144–57.

121 London School of Hygiene & Tropical Medicine. Peru: Countdown to 2015 case study. Futur. Learn.

122 Lutter C, Chaparro C. *Malnutrition in infants and young children in Latin America and the Caribbean : Achieving the and Millennium Development Goals*. 2008. http://www1.paho.org/hq/dmdocuments/2009/MalnutritionEng.pdf

123 Hamad R, Fernald LCH, Karlan DS. Health education for microcredit clients in Peru: a randomized controlled trial. *BMC Public Health* 2011;**11**.http://ovidsp.ovid.com/ovidweb.cgi?T=JS&CSC=Y&NEWS=N&PAGE=fulltext&D=caba6&AN=20113048729

124 McQuestion, M J, Velasquez A. Evaluating program effects on institutional delivery in Peru. *Health Policy (New York)* 2006;**77**:221–32. doi:doi:10.1016/j.healthpol.2005.07.007

125 Kim SS, Habicht JP, Menon P, *et al.* How do programs work to improve child nutrition? Program impact pathways of three nongovernmental organization intervention projects in the Peruvian highlands. IFPRI - Discuss. Pap. 2011. (1105)vii + 44 pp. 39 ref. 2011.http://ovidsp.ovid.com/ovidweb.cgi?T=JS&CSC=Y&NEWS=N&PAGE=fulltext&D=caba6&AN=20123112060

126 Francke P. UNICO Studies Series 11: Peru’s Comprehensive Health Insurance and New Challenges for Universal Coverage. Washington, D.C.: 2013.

127 Flores R, Rojas C. Impact of an Intervention on Food Security, REDESA Program Final Evaluation. Lima, Peru: 2007. http://www.globalbioenergy.org/uploads/media/1203_BEFSCI_Impacts_of_bioenergy_on_food_security.pdf

128 Flores R, Rojas C. Impact of an Intervention on Food Security, REDESA Program Final Evaluation. Lima, Peru: 2007.

129 General Directorate of People’ Health. Sexual and Reproductive Health.

130 Global Health Workforce Alliance. Peru. Heal. Work. all all Heal. Work. 2018.http://www.who.int/workforcealliance/countries/per/en/

131 Global Work Force Alliance. Mid-level health workers country case studies: Annex 12. Peru. Geneva, Switzerland: 2013. http://www.who.int/workforcealliance/knowledge/resources/MLHWCountryCaseStudies_annex12_Peru.pdf

132 Global Health Workforce Alliance. Peru. Heal. Work. all all Heal. Work. 2018.

133 Jimenez MM, Bui AL, Mantilla E, *et al.* Human resources for health in Peru: recent trends (2007–2013) in the labour market for physicians, nurses and midwives. *Hum Resour Health* 2017;**15**. doi:10.1186/s12960-017-0243-y

134 Andersen CT, Reynolds SA, Behrman JR, *et al.* Participation in the Juntos Conditional Cash Transfer Program in Peru Is Associated with Changes in Child Anthropometric Status but Not Language Development or School Achievement. *J Nutr* 2015;**145**:2396–405. doi:10.3945/jn.115.213546

135 Mayta-Tristán P, Poterico JA, Galán-Rodas E, *et al.* EL REQUISITO OBLIGATORIO DEL SERVICIO SOCIAL EN SALUD DEL PERÚ: DISCRIMINATORIO E INCONSTITUCIONAL. *Rev Peru Med Exp Salud Publica* 2014;**31**:781–7.http://www.scielo.org.pe/pdf/rins/v31n4/a26v31n4.pdf (accessed 2 Jan 2018).

136 Global Work Force Alliance. Mid-level health workers country case studies: Annex 12. Peru. Geneva, Switzerland: 2013.

137 Khwaja Y. Bioenergy and Food Security: The BEFS analysis for Peru. 2010. doi:ISSN 2071-0992

138 Khwaja Y. Bioenergy and Food Security: The BEFS analysis for Peru. 2010. doi:ISSN 2071-0992

139 Ministerio de Econmia y Finanzas. Programa Estrategico Salud Materno Neonatal. Lima, Peru:

140 Ministerio de Econmia y Finanzas. Programa articulado de nutrición. Lima, Peru: 2012.

141 PAHO. Peru. *Heal Am* Published Online First: 2012.http://www.paho.org/saludenlasamericas/index.php?option=com_docman&task=doc_view&gid=143&Itemid=

142 Ministerio de Desarrollo e Inclusion Social. ESTRATEGIA NACIONAL DE DESARROLLO E INCLUSIÓN SOCIAL: INCLUIR PARA CRECER. Lima, Peru:

143 The World Bank. Mortality rate, under-5 (per 1,000 live births). 2018.

144 Programma Articulado Nutricional.

145 Cordero L, Salhuana R. Systemisation of the Budget Support Program for the EUROPAN Structured Nutritional Program. Peru: 2015.

146 Iniciativa Contra la Desnutrición. Historia de la Iniciativa Contra la Desnutrición.

147 Schady NR. The political economy of expenditures by the Peruvian Social Fund (FONCODES), 1991-95. *Am Polit Sci Rev* 2000;**94**:289–95.

148 Ministerio de Salud (MINSA). Primera fase del programa de apoyo a la reforma del sector salud PARSALUD I. Lecciones aprendidas.
